# High body energy reserve influences extracellular vesicles miRNA contents within the ovarian follicle

**DOI:** 10.1371/journal.pone.0280195

**Published:** 2023-01-10

**Authors:** Natália Marins Bastos, Rodrigo Silva Goulart, Danilo Brito Bambil, Alessandra Bridi, Rosane Mazzarella, Luana Alves, Paola Maria da Silva Rosa, Adomar Laurindo Neto, Saulo Luz Silva, Miguel Henrique de Almeida Santana, João Alberto Negrão, Guilherme Pugliesi, Flávio Vieira Meirelles, Felipe Perecin, Juliano Coelho da Silveira

**Affiliations:** 1 Department of Veterinary Medicine, College of Animal Science and Food Engineering, University of São Paulo, Pirassununga, São Paulo, Brazil; 2 Department of Animal Science, College of Animal Science and Food Engineering, University of São Paulo, Pirassununga, São Paulo, Brazil; 3 Department of Animal Reproduction, College of Veterinary Medicine and Animal Science, University of São Paulo, Pirassununga, São Paulo, Brazil; 4 Department of Basic Science, College of Animal Science and Food Engineering, University of São Paulo, Pirassununga, São Paulo, Brazil; University of Guelph Ontario Agricultural College, CANADA

## Abstract

Aiming to evaluate the effects of increased body energy reserve (BER) in Nellore cows’ reproductive efficiency, cows were fed with different nutritional plans to obtain animals with high BER (HBER; *Ad libitum* diet) and moderate BER (MBER: cows fed 70% of HBER group ingestion). To evaluate the BER, cows were weekly weighted and evaluated for subcutaneous fat thickness and insulin serum concentration along the experimental period. At the end of the experimental period, animals were submitted to estrous synchronization and artificial insemination. Animals were slaughtered approximately 120 h after ovulation induction and the reproductive tracts were collected for embryo recovery and samples collection. Cumulus-oocyte-complexes (COC) and follicular fluid were collected from 3–6 mm in diameter ovarian follicles to perform miRNA analysis of cumulus cells (CC) and extracellular vesicles from follicular fluid (EV FF). As expected, differences were observed among MBER and HBER groups for body weight, fat thickness, and insulin serum concentration. HBER animals showed lower ovulation and embryo recovery rates compared to MBER animals. Different miRNAs were found among CC and EV FF within groups, suggesting that the BER may influence follicular communication. This suggests that small follicles (3–6 mm diameter) are already under BER effects, which may be greater on later stages of follicular development.

## Introduction

Animal nutrition acts on the development of body tissues, homeostasis, and metabolic system, reflecting on the animal’s body energy reserve (BER) and consequently directly affecting the reproductive performance of beef cows [1]. Animals in extreme conditions (too thin or too fat) have greater risks of metabolic changes, diseases, and reproductive problems such as dystocia, low conception rates, and prenatal/postpartum problems [2–4]. Moderate BER, that is, body condition score (BCS) 5, on a 1–9 scale, or 16% of body fat, proves to be optimal for beef cows pregnancy rate [4]. However, beef females with higher values than those (high BER, BCS up to 6, and body fat more than 20%) can have recurrent reproductive problems [3, 4]. Compared to cows with adequate body conditions during the reproductive season, beef cows with high BER presented pregnancy rate 8.8% lower through fixed-time artificial insemination (FTAI) [5]. Once bulls and cows with high BER represent most of the show cattle and genetic donors, the effects of the altered BER on the reproductive function of these animals must be elucidated.

The plane of nutrition of the herd has a rapid and cumulative effect over time on the reproductive function of cows as it is closely linked to BER, due to its effect on follicular development, oocyte/embryonic quality, and pregnancy rate [5–7]. The reason why this occurs is related to several physiological factors, including modulation of hypothalamic-pituitary-ovarian axis by hormones and metabolic substrates such as insulin [8], which plays important roles in reproductive physiology, acting directly on ovarian follicles, oocytes, and embryo development [1, 9–11]. Changes in insulin serum concentrations can alter ovarian response by modifying the follicular metabolic balance which allows changes in the local microenvironment influencing the gametic and subsequent embryonic quality [7].

The follicular environment, composed by theca cells, granulosa cells, cumulus cells, oocyte, and follicular fluid (FF) is influenced by autocrine, paracrine, and endocrine factors [12] and by bidirectional communication between somatic and gametic cells [13]. FF is an exudate from the capillaries blood flow around the follicles [14] that bathes and supports the communication between follicular cells. This communication process can also be mediated by extracellular vesicles (EVs) [15]. These bioactive nanoparticles are able to intermediate the cell communication by carrying lipids, proteins, mRNAs, and miRNAs that represent the partial secretory cell content to target cells [16–18]. MiRNA are small RNA molecules (approximately 22 nucleotides) that do not encode proteins (non-coding RNA). Its function is related to post-transcriptional regulation [19]. MiRNAs are transcribed in the nucleus and after being transferred to the cytoplasm they are processed and loaded into the RNA-induced silencing complex (RISC) to pair with the target messenger RNA in the 3’-UTR region, repressing its translation [19, 20]. Thus, intrafollicular cell communication mediated by EVs can occur by the transfer of bioactive material [13], such as miRNA, in response to environmental factors as metabolism [21].

Although different studies have described the diet effects [22], nutritional management [23, 24] and metabolism [6, 25] on ovarian response, oocyte and embryonic quality, little is known about these reproductive responses after increasing BER in beef cows. Therefore, the hypothesis for this work is that the increase of BER in Nellore cows negatively influences ovulation and embryo recovery rates by changes in the miRNA profile of extracellular vesicles present within the follicular environment. Thus, the aim of this work was to understand the effects of altered BER on ovulation, embryo recovery and miRNA profile of extracellular vesicles present within the follicular environment in multiparous Nellore cows with different BER.

## Material and methods

All experimental procedures were approved by the College of Animal Science and Food Engineering, University of São Paulo (FZEA/ USP) Ethics Committee (protocol number: 1522231019) and did not involve human subjects. The experiment was performed at Experimental Confinement unit from the Department of Animal Science, and at the Laboratory of Morphophysiology and Molecular Development (LMMD) of the Department of Veterinary Medicine, both located at the University of São Paulo (Campus of Pirassununga, SP, Brazil).

### Animals and nutritional management

Nellore cows from a single herd were previously evaluated by different parameters to select uniform animals. In this way, 40 Nellore cows were housed in four pens (10 x 16 m; 10 cows/pen; 16m^2^/cow) equipped with electronic gates (American Calan Inc., Northwood, NG, USA) that allow individual control of feed intake. The pens had concrete-floors and partial roof coverings. Cows had access to fresh ad libitum water during the entire experimental period.

**Fig 1:**
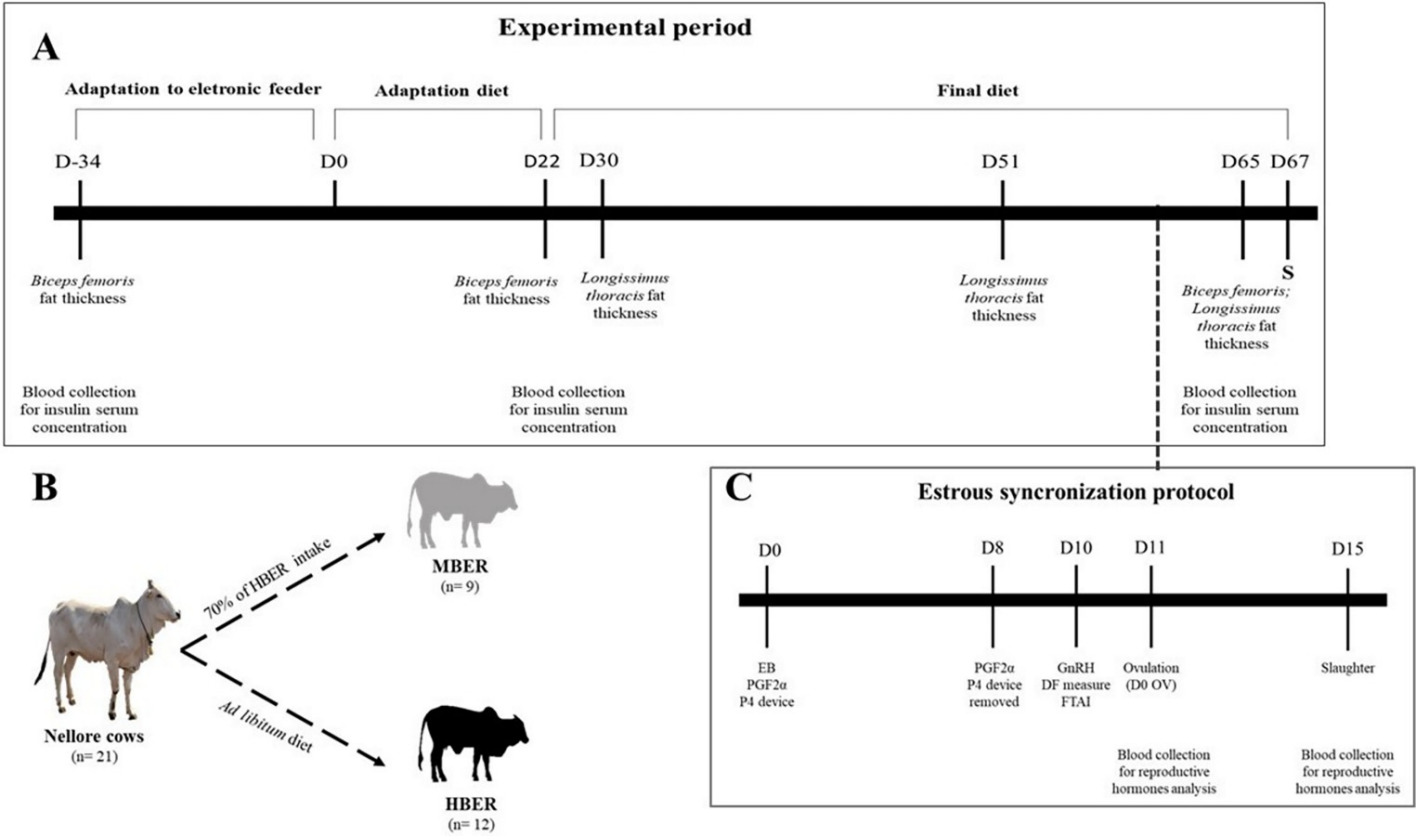
Schematic representation of experimental period. **A.** Timeline of feedlot period showing days of adaptation to electronic feeder (D-34 to D-1); adaptation diet (D0 to D21) and final diet (D22 to Slaughter (D67); S). During the feeding period the animals were submitted to: Subcutaneous fat thickness analysis of *Biceps femoris* (D-34, D22 and D65) and *Longissimus thoracis* (D30, D51 and D65); blood collection for Insulin serum concentration (D-34, D22 and D65); and weekly weighing average daily gain of each animal. **B.** Two nutritional plans were defined for cows with similar body conformations from a same herd in order to obtain cows with moderate body energy reserve (MBER) and cows with high body energy reserve (HBER) at the end of the experimental period. **C.** At the end of the experimental period, the animals were submitted to an estrous synchronization protocol (EB: estradiol benzoate, PGF2α: prostaglandin- 2α; P4: progesterone; GnRH: gonadotropin-releasing hormone), dominant follicle (DF) measure, artificial insemination (FTAI) and blood collection for reproductive hormones concentration (Day scheduled for ovulation; D0 OV and S).

Before beginning the trial, animals received, individually, free corns silage and mineral supplement ad libitum while adapting to the electronic gates (approximately 30 days; Fig 1A). Cows that did not adapt to the feeding system, were removed from the experiment. Thus, 21 Nellore multiparous (3,45 ± 0,54 number of births), non-lactating and non-pregnant cows (6.06 ± 0.54 years old and 1.44 ± 0.0083 m of withers height) were randomly divided in two groups with similar values for body weight (510.67 ± 15.55 kg) and Biceps femoris subcutaneous fat thickness (11.31 ± 1.20 mm) which represents similar values for BER, and submitted to an experimental period in order to became different in BER at the end of the feedlot period.

Thus, two nutritional plans were defined to obtain cows with moderate body energy reserve (MBER; n = 9) and cows with high body energy reserve (HBER; n = 12). For this, the animals belonging to the HBER treatment received ad libitum diet in order to increase body energy reserves during the experimental period that is, increasing body weight (BW) and fat thickness, while the other group (MBER) was fed 70% of the daily feed of HBER treatment (Fig 1B) to keep the BW and energy reserve throughout the experimental period.

The total feeding period had 67 days, including an adaptation period (21 days). The adaptation period consisted of 3 diets of 7 days each one (S1 Table) with increasing proportions of concentrate and corn silage ratio (40:60; 50:50 and 58:42, respectively) on DM basis, totalizing the 21 days as mentioned before. The final diet contained a 62.5:37.5 ratio of concentrate and corn silage (on DM basis; Table 1). The mineral supplement used contained Ca (208 g/kg); Co (148 mg/kg), Cu (2.7 mg/kg), S (64 g/kg), F (1.6 mg/kg), P (160 g/kg), I (141 mg/kg), Mn (2.2 mg/kg) Se (37 mg/kg), Zn (79.92 mg/kg) and sodium monensin (4000 mg/kg). Diets were formulated to meet the nutritional requirements of Nellore cows, according to Cornell Net Carbohydrate and Protein System (CNCPS; [26]).

**Table 1:** Ingredients and chemical composition on a dry matter (DM) basis (g/kg) of final diet.

Ingredients^1^	g/kg of DM
Corn silage	375
Finely ground corn	510
Soybean meal	83.8
Mineral supplement	6
Limestone	9.2
Urea	6.9
Potassium chloride	5.0
Salt	4.3
**Chemical composition**	
Dry matter	523.5
Mineral matter	46.0
Crude protein	151.3
Ether extract	30.9
Acid detergent fiber	207.3
Neutral detergent fiber	296.7
Total digestible nutrients	775.3

^1^Ingredients: Mineral supplement: Ca (208 g/kg); Co (148 mg/kg), Cu (2.7 mg/kg), S (64 g/kg), F (1.6 mg/kg), P (160 g/kg), I (141 mg/kg), Mn (2.2 mg/kg) Se (37 mg/kg), Zn (79.92 mg/kg) and sodium monensin (4000 mg/kg)

The experimental diet was offered individually three times a day, using the following proportion: 30% of the daily offer at 8:00 am, 40% at 2:00 pm and 30% at 6:00 pm. Orts from the HBER group that should not exceed 50 g/kg were removed daily, weighted and used to determine the amount of feed to be offered on the next day based on the dry matter intake (DMI) of the previous day. Samples of the total diet were collected weekly for further chemical analysis.

In order to reach the maintenance and progression of energy reserves in MBER and HBER cows, respectively, the animals were weighted weekly to determine the BW. Analyzes of rump fat thickness and blood collection to serum concentration of insulin were performed throughout the experimental period as the HBER animals increased BW (Fig 1A). The experimental period lasted according to the phenotype of HBER animals, which mean be different from the MBER measured by the different analysis. When the two phenotypes (MBER and HBER) were achieved, the experimental period has come to an end. The amount of weight that the animals gained daily during the entire experimental period was counted, obtaining the average daily gain (ADG), and after slaughter, was obtained the hot carcass weight and carcass yield.

### Feed sample collection and chemical analysis

Diet samples were collected weekly throughout the study and dried at 105°C for 24 h [27] for dietary DM adjustments. Samples were stored at -20°C until the end of experiment when they were grouped into composite samples, dried in a forced-air oven at 55°C for 72 h, and ground with a 1 mm screen (Wiley mill, MA-680 Marconi Ltda, Piracicaba, SP, Brazil) for subsequent analyses. The samples were analyzed for DM in an oven at 105°C for 24 h (AOAC, 1990), mineral matter (MM; [27]), crude protein (CP) by the Kjeldahl method [28], ether extract (EE; [27]), neutral detergent fiber (NDF; non-sequential and ash-free; [29]), acid detergent fiber (AFD) and acidic lignin as method 973.18 [29], and total digestible nutrients (TDN; [30]) (Table 1). The NDF assay used a heat stable source of amylase and urea as recommended by the National Forage Testing Association [31]. Each sample received α-amylase (Sigma A3306; Sigma-Aldrich Brazil Ltda, São Paulo, SP, Brazil) and urea separately for NDF determination. Both NDF and ADF were expressed including residual ash (Table 1).

### Subcutaneous fat thickness analysis

Subcutaneous fat thickness was evaluated by ultrasonography (SSD 500 Micrus, Aloka Co. Ltd.) with a linear transducer of 3.5 mHz and 172 mm in length over the *Biceps femoris* (between the ilium and ischium) and also over the *Longissimus thoracis* (between the 12^th^ and 13^th^ rib) muscles [32] were collected and analyzed using the Lince® software (M&S Consultoria Agropecuária Ltda. Pirassununga, SP, Brazil). Analyzes of subcutaneous fat thickness were performed over *Biceps femoris* on days -34, 22 and 65 of the experimental periods (Fig 1A). The *Longissimus thoracis* ultrasonography analyses were performed after starting the final diet on days 30, 51, 65 (Fig 1A).

### Blood collection and biochemical analysis

Blood samples from all animals were collected from the jugular vein in silica vacutainer tubes (one collection per animal; BD, São Paulo/SP, Brazil). Blood serum was obtained by centrifugation at 1500xg for 30 min in room temperature and was used to quantify insulin, estrogen (E2), progesterone (P4) and the biochemical profile.

To quantify serum insulin concentration, blood samples were collected on days -34, 22 and 65 of the experimental period (Fig 1A). Hormone concentration were performed by enzyme-linked immunosorbent assay (ELISA; Insulin Accubind ELISA, 1936, Monobind), by the Animal Physiology Laboratory of the University of São Paulo (FZEA/USP) [33–35]. Intra and interassay variation coefficients were 4.40% and 6.35%, respectively.

Blood samples to quantify serum concentrations of the reproductive hormones E2 and P4 were collected after the start of the estrous synchronization protocol, on the day scheduled for ovulation (D0 OV) and on the day of slaughter (S1C Fig). Serum hormone measurements were performed using chemiluminescence (ADVIA Centaur ®—Siemens) by the Pasin laboratory (Santa Maria–RS) as previously used by De Ávila et al. [15]. Intra and interassay variation coefficients were 3.30% and 24.20% respectively for E2; 0.21% and 0.20%, respectively for P4.

### Estrous synchronization protocol, artificial insemination, slaughter and embryo recovery

At the end of the experimental period, animals were submitted to estrous synchronization (sync) protocol (Fig 1C). On the first day (D0 sync), cows received 2 ml of estradiol benzoate (2 mg; Sincrodiol®, Ourofino Agronegócio) intramuscularly, 2 ml of PGF2α (0.52 mg; Prostaglandin- 2α; Sincrocio®, Ourofino Agronegócio) intramuscularly and insertion of an intravaginal progesterone device (1g; Sincrogest®), Ourofino Agronegócio) which was withdrawn on the 8^th^ day (D8 sync). Still on D8 sync, animals received 2 mL of intramuscular PGF2α at the time of removal of the intravaginal progesterone device. In D10 syn 2.5 mL of GnRH (0.0010 mg; Gonadotropin-releasing Hormone; Sincroforte®, Ourofino Agronegócio) was administered intramuscularly, the diameter of the dominant follicle (DF) was analyzed by ultrasound (MyLab Delta, Esaote, Italy), and after 12 h of GnRH administration it was performed the artificial insemination. All animals were inseminated with semen from a single bull with previously assessment confirmation of fertility. Confirmation of ovulation was performed 12 h after fixed-time artificial insemination (FTAI) by ultrasound (D0 OV).

Approximately 120 h after ovulation induction, cows were slaughtered (in FZEA/USP abattoir in accordance to the Humanitarian Slaughter Guidelines required by Brazilian laws) and the reproductive tract removed, which was immediately transported to the laboratory to obtain the samples (S1A and S1B Fig). The oviducts ipsilateral (same side) to the corpus luteum were dissected (S1C Fig) and the isthmus portion was flushed in the ovary-uterus direction with 1 mL of phosphate-saline solution free of calcium and magnesium (1xPBS free of Ca^2+^ and Mg^2+^) for embryo recovery with the aid of a magnifying glass (S1D and S1E Fig).

### Follicular aspiration

From the 21 cows used at the experimental period (MBER: n = 9; HBER: n = 12), only animals which presented ovulation and absence anovulatory follicle at D0 OV had the ovaries ipsi and contralateral collected (MBER: n = 8; HBER: n = 4) to obtain cumulus cells (CC) and follicular fluid extracellular vesicles (FF EVs). Ovarian follicles measuring 3–6 mm in diameter (S1F Fig) were punctured with an 18G needle and a 10 mL syringe to obtain cumulus-oocyte-complexes (COC) and follicular fluid (FF).

Groups of five COCs were pooled from ipsi and contralateral ovarian follicles (totalizing 10 COCs per animal) and completely denuded in 1xPBS free of Ca^2+^ and Mg^2+^ in drops by pipetting. The CC obtained from COCs groups (one pool per each ipsi and contralateral ovaries per cow; MBER: n = 8; HBER: n = 4), were centrifuged twice at 300xg for 5 min and after having the supernatant removed, the cells were immediately frozen in liquid nitrogen for further analysis of the miRNA content. FF was collected to obtain EVs (from all aspirated follicles in ipsi and contralateral ovaries; MBER: n = 8; HBER: n = 4) to perform FF EV characterization and miRNA profile.

### Isolation of small extracellular vesicles from follicular fluid

From the FF collected reported above, was obtained the FF EVs as previously described by De Ávila et al. [15]. Briefly, the samples were diluted in 500μl of 1xPBS free of Ca^2+^ and Mg^2+^ and centrifuged at 4°C at 300xg for 10 min to remove cells, 2000xg at 4°C for 10 min to remove cell debris, 16500xg at 4°C for 30 min to remove larger extracellular vesicles. To obtain the pellet enriched in small extracellular vesicles (smaller than 200 nm), 800μl of the resulting supernatant were filtered through a 0.20μm pore filter (Corning, 431229) and ultracentrifuged at 119700xg by 70 min at 4°C (Optima XE-90 Ultracetrifuge; 70 Ti rotor; Beckman Coulter). Then, the obtained pellet was washed in 1xPBS and ultracentrifuged again at 119700xg for 70 min to 4°C. The pellet enriched with small extracellular vesicles was eluted in 50μl of 1xPBS free of Ca^2+^ and Mg^2+^ for further characterization and miRNA content analysis.

### Characterization of small extracellular vesicles from follicular fluid

The EVs FF characterization consists of nanoparticle tracking analysis (NTA), transmission electron microscopy and qualitative protein markers by Western Blot following the Minimal Information for Studies of Extracellular Vesicles 2018 (MISEV 2018) recommendations [36]. To perform the transmission electron microscopy and Western Blot analyses, slaughterhouse samples were collected and processed with the same protocol to avoid the lack experimental samples of EVs FF for tracking nanoparticles and miRNA content analysis.

To perform NTA analysis, after EVs FF isolation, 5μl of the eluted pellet was diluted in 995μl of 1xPBS free of Ca^2+^ and Mg^2+^, of which 10μl were diluted again in 990μl of 1xPBS free of Ca^2+^ and Mg^2+^ for particle size and concentration evaluation using the Nanosight device (NS300; NTA 3.4 Build 3.1.45; Malvern). Five 30-second videos were taken at a controlled temperature of 38,5°C, Camera Level 13 and the analyzes were performed considering threshold 5.

For transmission electron microscopy, after isolation by serial centrifugation, the EVs pellets were diluted in a fixative solution (0.1M Cacodylate; 2.5% Glutaraldehyde; 4% paraformaldehyde; pH 7.2–7.4) for 2 h to be ultracentrifuged again and resuspended in 20 μl of ultrapure milli-Q water. The analyzes were realized at the Multiuser Laboratory of Electronic Microscopy of the Department of Cellular and Molecular Biology, Faculty of Medicine of Ribeirão Preto, using a transmission electron microscope (FEI 200kV, model Tecnai 20, emitter LAB6).

To verify the presence of specific EVs proteins in FF by Western Blot, after EVs isolation protocol, samples were resuspended in 20 uL of lysis buffer (RIPA), stored on ice under constant agitation. The samples were applied on SDS-PAGE polyacrylamine gel 6% and 15% for better representation of the proteins, and the run was performed at 100 V for approximately 120 min prior to the wet transfer to nitrocellulose membrane at 80 V for 120 min. After the transfer, membranes were kept 1 h in blocking solution (3% nonfat dry milk in TBST–Tris buffered saline with Tween-20), prior to the incubation with primary antibodies overnight at 4°C. The specific vesicle proteins studied were ALIX (rabbit, sab4200476, Sigma-Aldrich, St. Louis, MO, USA), CD9 (mouse, sc-13118, Santa Cruz, CA, USA) and the non-vesicular protein control was GRP78 (protein from endoplasmic reticulum; mouse, sc-376768, Santa Cruz) [36]. After the incubation period, the membrane was washed three times in TBST for 5 min and maintained for 1 h in anti-rabbit (A0545sc-2357 Sigma-Aldrich, St. Louis, MO, USA) and anti-mouse (#7076S, Cell Signaling Technology) secondary antibody. Then, the secondary antibodies were removed, the membrane washed and exposed to the developing solution (170–560, Clarity Western ECL), for analyzes carried out by the ChemiDoc MP Image System (Bio-RAd, Hercules, CA, USA).

### Total RNA extraction and miRNA analysis

Total RNA content from CCs and EVs FF (MBER: n = 8; HBER: n = 4) was extracted using Qiazol (QIAGEN) according to the manufacturer’s instructions, adding 1.33μl of GlycoBlue coprecipitator (Thermo Fisher Scientific) to the aqueous phase before RNA precipitation [37]. RNA quantity and quality were analyzed by spectrometry (NanoDrop 2000, Thermo Fisher Scientific; absorbance ratio 260/280nm) and treated with DNaseI (Invitrogen; Carlsbad, CA) according to the manufacturer’s instructions. After extraction, the RNA was stored at -80°C until use.

The total RNA was reverse transcribed into cDNA using the miScript II RT kit (QIAGEN), using the miScript HiSpec Buffer to obtain mature miRNAs in CC and miScript HiFlex Buffer to obtain mature and precursor miRNAs in FF EVs. CC reactions contained 100 ng of total RNA, while for EVs FF reactions were performed with 200 ng of total RNA. Both were performed in a thermocycler (Life Technology) at 37°C for 60 min, followed by 95°C for 5 min. The RT-PCR reactions to quantify the transcripts were performed according to Da Silveira et al. [37], using at least 0.2 ng of cDNA and 1μl of 10 mM forward primers obtained based on the mature bovine miRNAs sequences available in the mirBase software (http://www.mirbase.org). The temperatures and times used were 95°C for 15 min followed by 45 cycles of 94°C for 15 seconds, 55°C for 30 seconds and 70°C for 30 seconds. For each sample (CC and FF EV) and group (MBER: n = 8; HBER: n = 4), the expression analysis of 383 bovine miRNAs was performed using the same forward sequences as published before [37, 38]. The miRNAs were considered present when they presented a cycle threshold (CT) lower than 37 in all biological repetitions and an appropriate melting curve in more than 50% of samples; the miRNAs were considered exclusive when were expressed in all samples versus none samples. CT data generated by amplification were normalized using bta-miR-99b as previously published [39]. The miRNAs differently expressed between groups were evaluated by miRWalk software version 3.0 in order to identify predicted regulated pathways. Predicted pathways were considered significant when adjusted *P*-value < 0.05.

### Statistical analysis

The animal’s BER (BW and fat thickness), and hormone levels (insulin, E2 and P4) data were analyzed using a model including the fixed effects of the BER class (MBER and HBER), the time class (days in experimental period), and the interaction between BER and time. When significant effects were found (*P*<0.05), the least-square means were compared using the Tukey test. The heigh of withers, DMI, ADG, carcass traits (weight and yield), response to the estrous synchronization protocol, EVs size and concentration and miRNA data were analyzed using the fixed effect of the body energy reserve. The means were adjusted by the least-squares method and compared using the probability of difference using the Student’s *t* test. The reproductive analyzes (ovulation, and embryo recovery rate) were analyzed using the chi-square test. All the analyses utilized the program “JMP” (7.01 version, Statistical Analysis Software Institute, SAS® Inc., Cary, NC). A significant difference was declared when *P*<0.05.

## Results

### Feedlot performance and carcass traits

**Fig 2:**
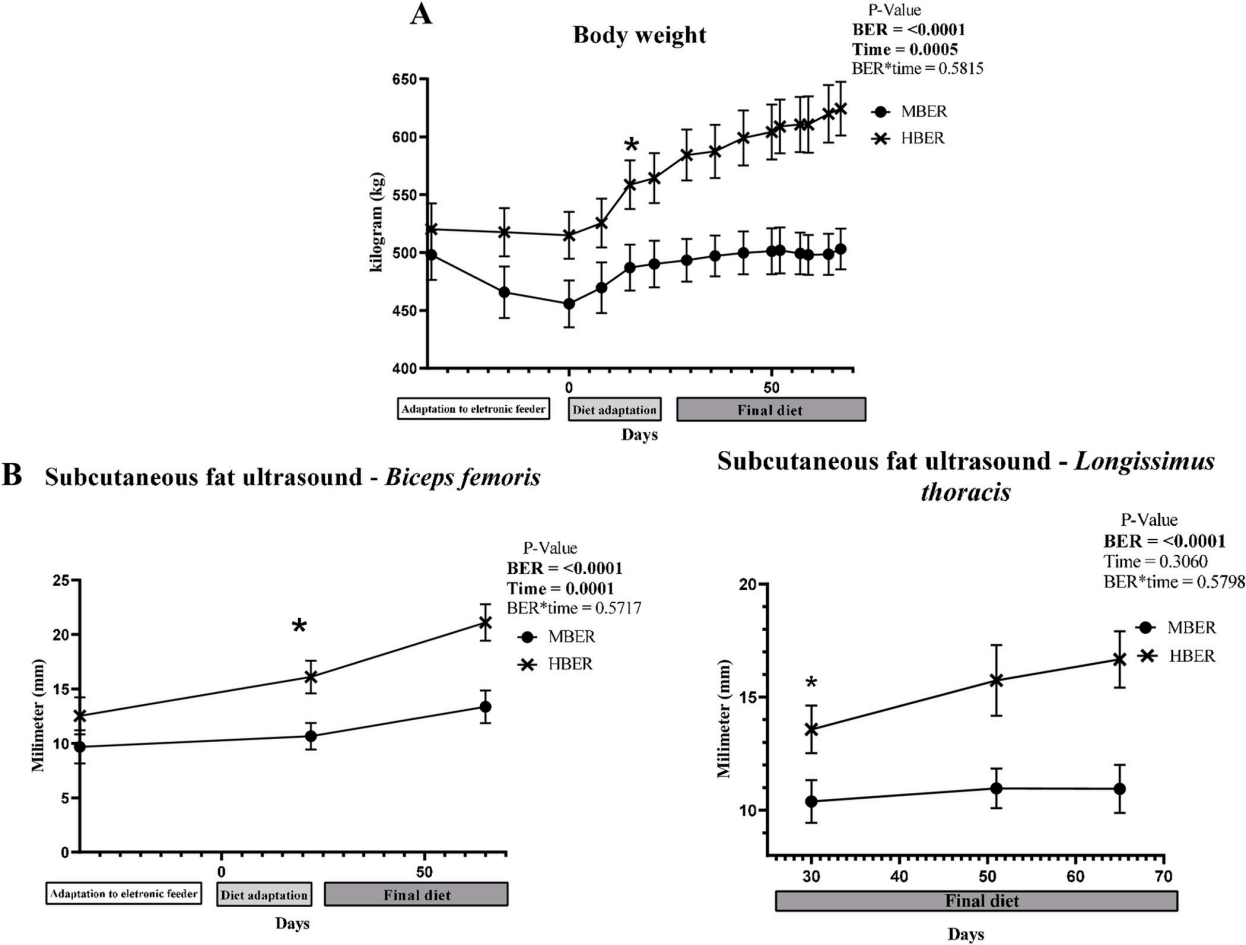
Body weight and subcutaneous fat ultrasound of Nellore cows during the experimental period. **A.** Evolution of body weight of cows with different energy reserves. **B.** Analysis of subcutaneous fat thickness of *Biceps femoris* and *Longissimus thoracis* measured by carcass ultrasound of cows with different energy reserves. Mean standard error for body energy reserve, time and body energy reserve and time interaction. P-values are on the right top of the Figs. *Beginning and prevalence of statistical differences (*P*<0.05).

In order to establish two experimental groups, 21 cows were divided into moderated and high BER (MBER: n = 9; HBER: n = 12), animals with similar BER and wither height, were submitted to different DMI according to the experimental groups (MBER and HBER) presenting different DMI (*P*<0.0001) and ADG (*P*<0.0001) until the end of the experimental period, being HBER with higher values than MBER animals (S2 Table). As expected, similar cow’s BW was observed at the beginning of the study (D-34; MBER: 498.00 kg ± 24.49 kg; HBER: 520.17 ± 22.32 kg, *P* = 0.4947, Fig 2A); however, at the end of the experimental period the HBER group was 121.22 kg heavier than MBER group (at D67: MBER: 503.11 kg ± 17.66; HBER: 624.33 ± 23.25 kg, *P* = 0.0009). At slaughter (D67), animals with different body energy reserves had different hot carcass weight (MBER: *P* = 0.002) and carcass yield (*P* = 0.0331; S2 Table).

*Biceps femoris* fat thickness measurements by ultrasound were similar at the beginning of the study for the two groups of cows (MBER: 9.68 ± 1.53 mm; HBER: 12.53 ± 1.72 mm, *P* = 0.2478). However, at the end of the feedlot period (D67), cows from HBER group had superior *Biceps femoris* fat thickness (21.11 ± 1.68 mm) in comparison with MBER (13.35 ± 1.51 mm, *P* = 0.0037, Fig 2B). For the *Longissimus thoracis*, the analyzes performed by ultrasound after the beginning of the finishing diet (MBER: 10.38 ± 0.94 mm; HBER: 13.57 ± 1.05 mm, *P* = 0.0425) and the end of the experimental period (MBER: 10.94 mm ± 1, 06; HBER: 16.67 ± 1.24 mm, *P* = 0.0034, Fig 2B) demonstrated different values for animals with different body energy reserves (S2 Fig).

### Hormone analysis

**Fig 3:**
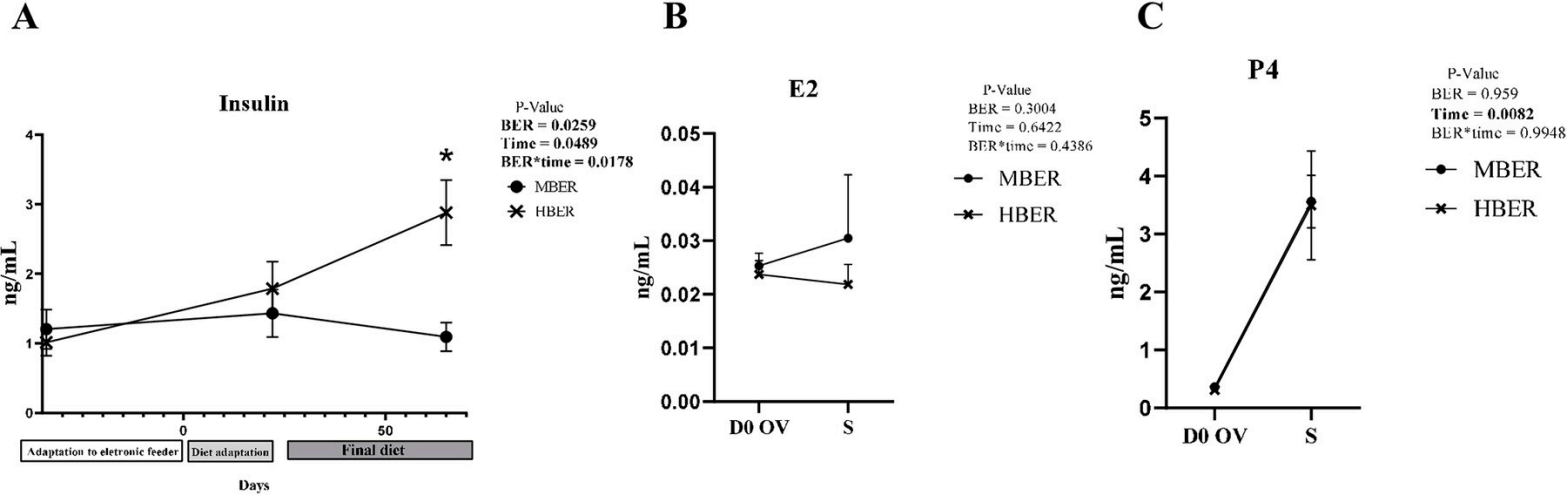
Serum insulin and reproductive hormones levels of Nellore cows during the experimental period. **A.** Insulin concentration. **B.** E2 concentration. **C.** P4 concentration. Standard error of the mean for body energy reserve, time and body energy reserve and time interaction. P-values are on the right top of the Figures. *Statistical difference (*P*<0.05). D0 OV: day scheduled for ovulation. S: day of slaughter.

The serum concentration of Insulin was analyzed in animals with different BER (MBER: n = 9; HBER: n = 12) along the experimental period. There was an interaction between BER and time for serum insulin concentrations (*P* = 0.0178, Fig 3A) indicating that BER across the time was responsible for the increased insulin serum concentration.

There was no difference after the estrous synchronization protocol for estrogen (*P* = 0.3004, Fig 3B) and progesterone (*P* = 0.9590, Fig 3C) levels between animals with different BER (MBER: n = 9; HBER: n = 12). However, the serum concentration of progesterone varied over time, between the day of ovulation (D0 OV) and slaughter (*P* = 0.0082).

### Response to estrous synchronization protocol, ovulation and embryo recovery rates

The diameter of the dominant follicle did not differ in animals with different BER (Table 2). Additionally, we evaluated the presence of anovulatory follicle at D0 OV, which was similar between groups (Table 2). However, MBER animals have higher ovulation rates (MBER: 9/9; HBER: 7/12, *P* = 0.0094, Table 2), when compared to HBER animals. Based on the animals in which ovulation was confirmed, the embryo recovery rate of the MBER (7/9) and HBER (3/7) animals was calculated, which was higher (*P* = 0.0121; Table 2) for the MBER group.

**Table 2:** Least squares mean, standard error of mean and probability of response to the estrus synchronization protocol and reproductive rates of cows with different body energy reserve.

Reproductive analyses^1^	Body energy reserve^3^		P- value^4^
	MBER	HBER	
DF, mm	14.33 ± 0.79	13.92 ± 1.06	0.7696
**Reproductive rates** ^**2**^			
Anovulatory follicle at D0 OV, %	11.11	33.33	0.2211
Ovulation, %	100	58.33	**0.0094**
Embryo recovery rate %	77.78	42.86	**0.0121**

^1^Response to the estrus synchronization protocol: DF: Dominant follicle diameter.

^2^Reproductive rates: Anovulatory follicle at D0 OV: Anovulatory follicle at the day scheduled for ovulation.

^3^Body energy reserve: MBER: Cows with moderated body energy reserve; HBER: Cows with high body energy reserve

^4^P-value: P value between animals with different body energy reserve.

### Extracellular vesicles characterization

The size and concentration of particles obtained by isolation were evaluated by NTA (S3A Fig) and, there were no differences for size (MBER: 159.45 ± 3.57 nm; HBER: 168.27 ± 5.58 nm, *P* = 0.1969, S3B Fig) and particle concentration (MBER: 1.00 x 10^12^ ± 1.30 particles/mL x 10^11^; HBER: 1.39 x 10^12^ ± 2.48 x 10^11^ particles/mL, *P* = 0.6032, S3B Fig). The Western blot analysis allowed to verify the presence of characteristic proteins of EVs (S3C Fig). ALIX and CD9 proteins were found in EV lysate, while endoplasmic reticulum marker proteins (GRP78) was only present in follicular cells. Transmission electron microscopy images demonstrate the presence of small extracellular vesicles in the follicular fluid (S3D Fig). These results demonstrate the efficiency of the EV FF isolation protocol.

### Cumulus cells and follicular fluid extracellular vesicles miRNA content

**Fig 4:**
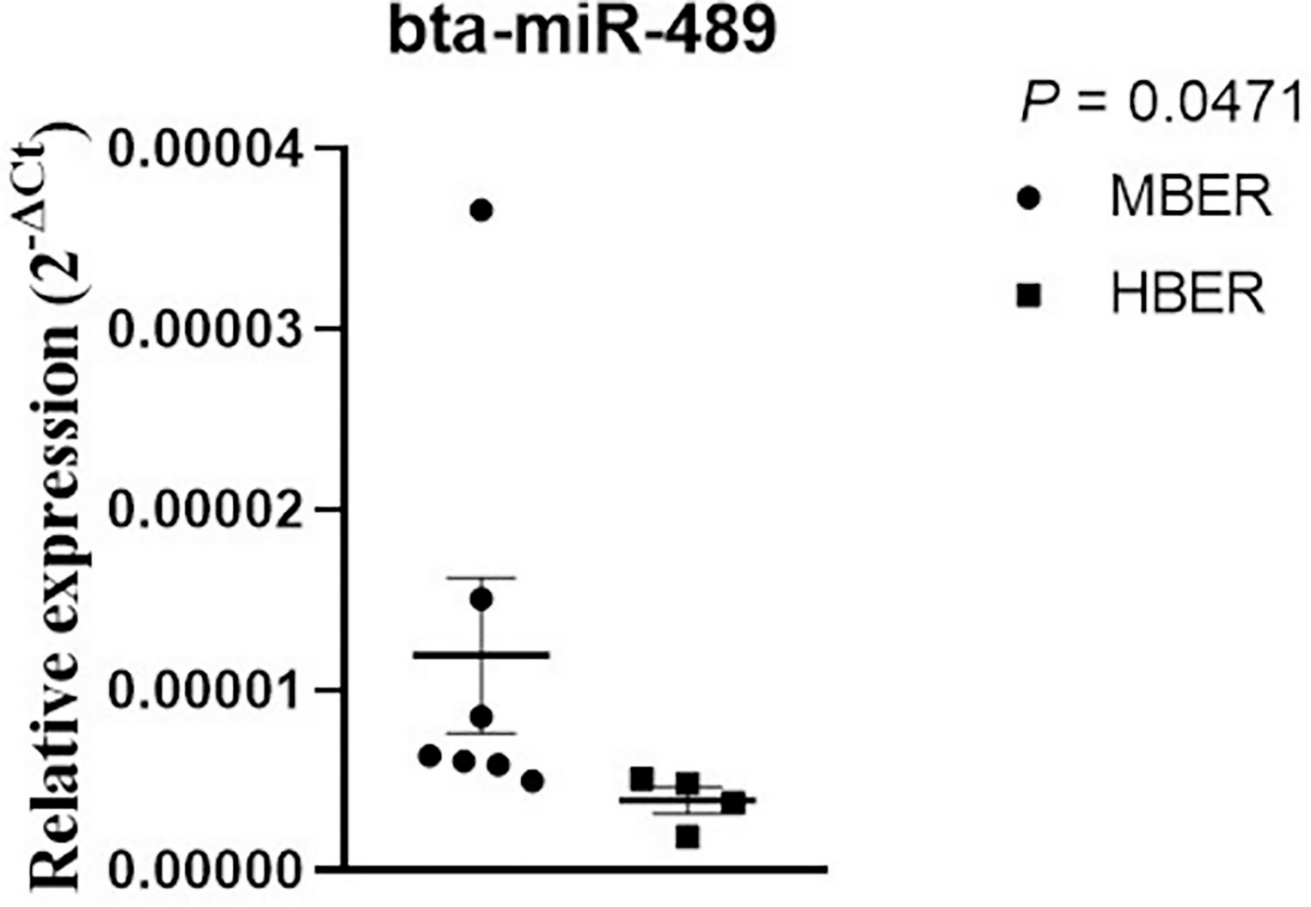
Relative expression of bta-miR-489 which is higher in the follicular fluid extracellular vesicles in MBER group. Mean ± standard error of the mean. P-value is in bold on the right top of the figure.

**Fig 5:**
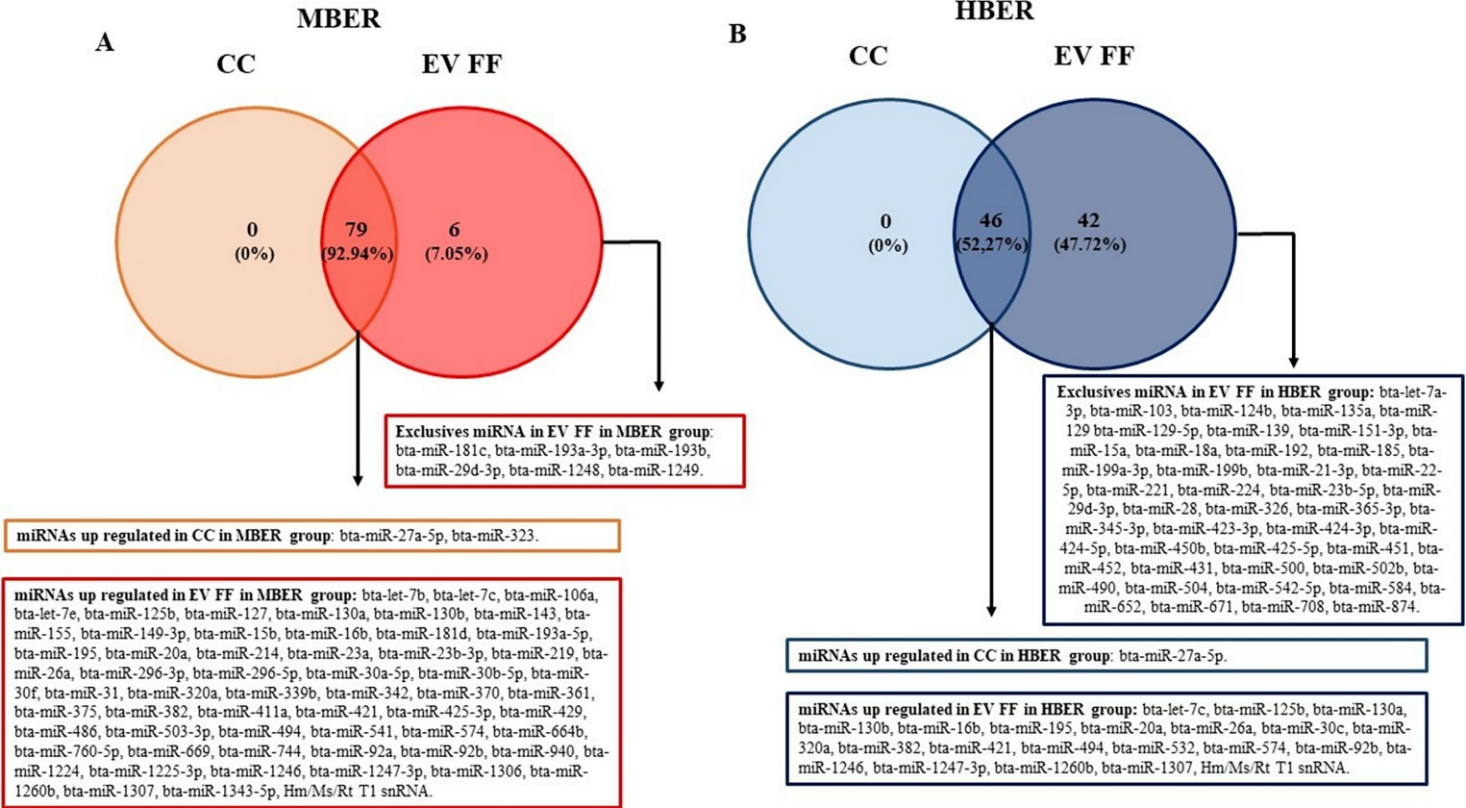
Total numbers of miRNAs detected in cumulus cells (CC) and follicular fluid extracellular vesicles (EV FF) from 3–6 mm follicles from cows with different body energy reserve. **A.** Venn diagram demonstrating a total of 79 miRNAs commonly detected between CC and EV FF for MBER group, which 2 were up regulated in CC samples and 60 up regulated in EV FF and, 6 exclusively detected miRNAs in EV FF but not in CC. **B.** Venn diagram demonstrating a total of 46 miRNAs commonly detected between CC and EV FF for HBER group, only 1 was up regulated in CC samples and 21 up regulated in EVs FF and, a total of 42 exclusively detected miRNAs in EV FF but not in CC.

To understand the influence of BER on the follicular environment, the profile of 383 bovine miRNAs was analyzed in CC and EV FF only from ovulated animals with no anovulatory follicle at D0 OV (MBER: n = 8; HBER: n = 4; S3 and S4 Tables). The mean repeatability of the samples in CC was 0.77 and 0.81 for the MBER and HBER groups, respectively, while in the EV FF it was 0.82 and 0.84 for MBER and HBER groups, respectively. A total of 40 mature miRNAs common between groups were detected in CC samples with no statistical differences. Similarly, the EV FF analysis demonstrated a total of 264 mature and precursor miRNAs common among groups, which only the bta-miR-489 miRNA was increased in the MBER group (Fig 4). However, aiming to evaluate possible effects on cell communication between follicular compartments, the relationship between the miRNA contents in CC and EV FF within each experimental group was evaluated by a simple analysis comparing the miRNA presence among samples for each BER category, without performing an interaction analysis. Thus, for MBER group (Fig 5A), there are 6 exclusives miRNA were detected in EV FF (bta-miR-181c, bta-miR-193a-3p, bta-miR-193b, bta-miR-29d-3p, bta-miR-1248 and bta-miR-1249; S5 Table) and 79 miRNAs commonly detected between CC and EVs, in which two of them are up regulated in CC (bta-miR-27a-5p and bta-miR-323) and 60 up regulated in EV FF (S6 Table). Comparing the CC miRNA content with EV FF for HBER group (Fig 5B), there were 42 exclusives miRNA detected in EV FF compared to CC (S7 Table) and 46 miRNAs commonly detected between CC and EVs, in which one (bta-miR-27a-5p) is up regulated in CC and 21 are up regulated In EVs (S8 Table).

### Enrichment analysis of miRNA differentially expressed in cumulus cells and small extracellular vesicles from follicular fluid

**Fig 6:**
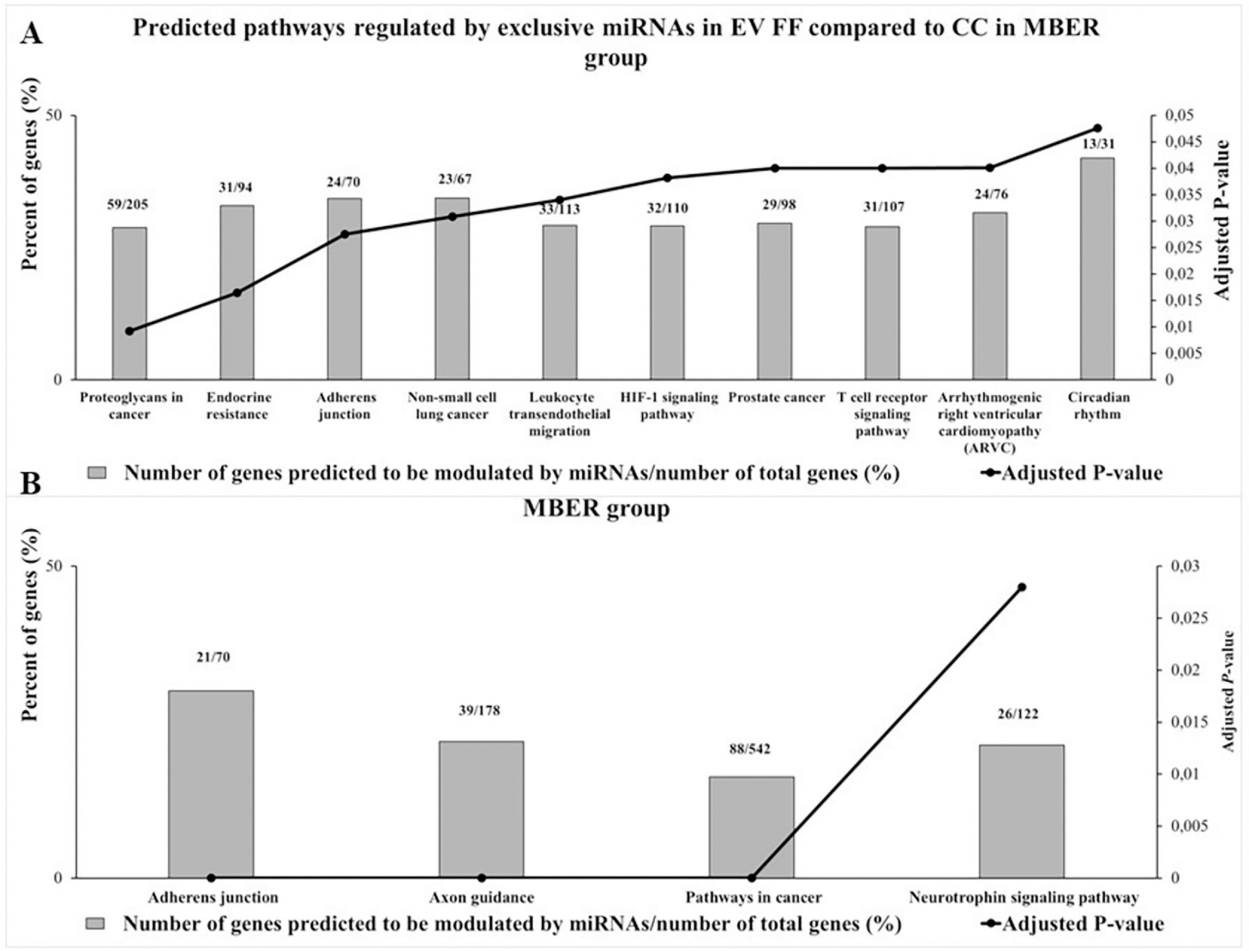
Enrichment analysis performed in miRWalk 3.0 software of predicted pathways modulated by exclusives or up-regulated miRNAs of cumulus cells (CC) and follicular fluid extracellular vesicles (EV FF) from 3–6 mm follicles from cows with moderated body energy reserve (MBER). **A.** The 10 predicted pathways with highest percent of genes predicted to be modulated by exclusives miRNAs in EV FF compared to CC in MBER group. **B.** The predicted pathways modulated by miRNAs up regulated in CC compared to EV FF in MBER group. The Y-axis in left represents the percent of genes (%) predicted to be modulated by miRNAs and the Y-axis in right shows the adjusted *P*-value (Adjusted *P*-value < 0.05).

To determine the predicted biological functions regulated by miRNAs differently expressed in CC and EV FF we performed bioinformatics analysis for the exclusively detected miRNAs in EV FF compared to CC for MBER group. The analysis revealed a total of 275 predicted pathways (S9 Table), being 26 significant in which 10 of them, with the highest percent of genes predicted to be modulate by those miRNAs are represented in Fig 6A. These miRNAs are predicted to regulate pathways like Proteoglycans in cancer, Endocrine resistance, Adherens junction, HIF-1 signaling pathways and Circadian rhythm. For the differently expressed miRNAs in CC compared to EV FF, the up regulated miRNAs in CC are predicted to modulate Adherens junction, Axon guidance, Pathways in cancer and Neurotrophin signaling pathway (Fig 6B), while the up regulated miRNAs in EVs are predicted to modulate Metabolic pathways (S10 Table).

**Fig 7:**
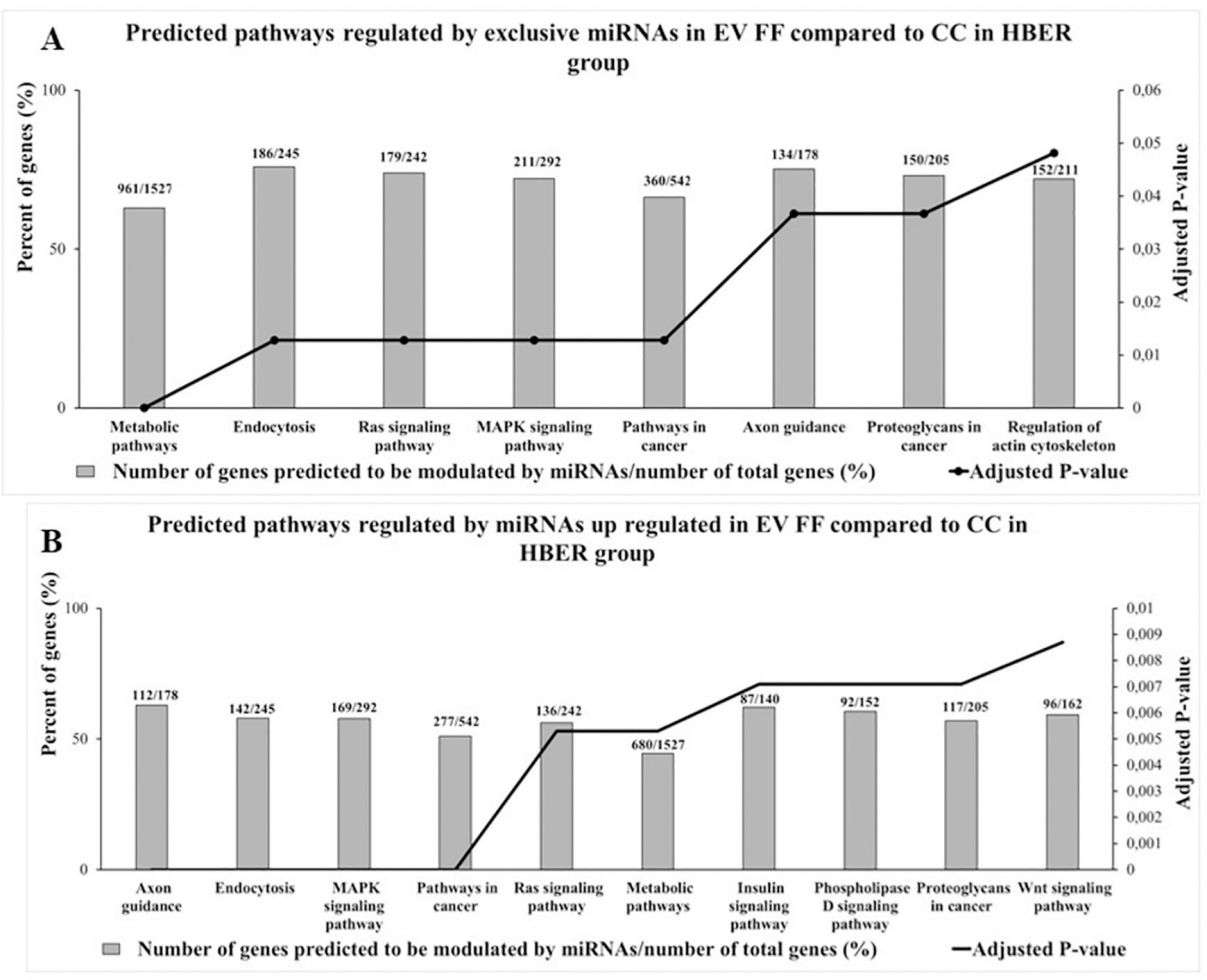
Enrichment analysis performed in miRWalk 3.0 software of predicted pathways modulated by exclusives or up-regulated miRNAs of cumulus cells (CC) and follicular fluid extracellular vesicles (EV FF) from 3–6 mm follicles from cows with high body energy reserve (HBER). **A.** The predicted pathways modulated by exclusives miRNAs in EV FF compared to CC in MBER group. **B.** The 10 predicted pathways with highest percent of genes predicted to be modulated by miRNAs up regulated in EV FF compared to CC in HBER group. The Y-axis in left represents the percent of genes (%) predicted to be modulated by miRNAs and the Y-axis in right shows the adjusted *P*-value (Adjusted *P*-value < 0.05).

For HBER group, the exclusive detected miRNAs in EV FF compared to CC are predicted to regulate 321 predicted pathways (S11 Table) which 8 are significant and are represented in Fig 7A. For the differently expressed miRNAs in CC compared to EV FF, the up regulated miRNAs in CC are predicted to modulate 320 signaling pathways (S12 Table) which 57 are significant and the 10 with the highest percent of genes predicted to be modulated by those miRNAs are represented in Fig 7B. Our results demonstrated that exclusives miRNAs in EV FF compared to CC are predicted to regulate Metabolic pathways, Endocytosis, Regulation of actin on cytoskeleton, Ras and MAPK signaling pathways, while the miRNAs up regulated in EV FF compared to CC are predicted to regulate Metabolic pathways, MAPK, Ras, Wnt, and Insulin signaling pathways.

## Discussion

Due to the importance of body condition for the reproductive response, this study aimed to understand the effects of altered BER on ovulation, embryo recovery and miRNA profile of extracellular vesicles present within the follicular environment in multiparous Nellore cows with different BER. Thus, two different nutritional plans were applied in which animals in HBER treatment had higher values for DMI and ADG changing the animal’s BW and subcutaneous fat thickness. Thus, despite the homogeneity between the groups at the beginning of the experiment (D-34), we demonstrated that at the end of the experimental period (S), animals differed in BER with HBER animals having higher BER than MBER animals.

Importantly, most of the studies involving the effects of nutrition and metabolism on reproductive function were performed in dairy cattle with negative energy balance, during the postpartum period, or submitted to a multiple ovulation protocol [25, 40, 41]. Thus, the correct comparison between studies is essential for a better comprehension of the nutritional effects on reproductive function. The present work evaluated multiparous, non-lactating and non-pregnant Nellore cows (beef cattle), whose DMI was obtained daily and individually for a long period (67 days). Other studies aiming to understand the effects of nutritional management and metabolism on reproductive efficiency also validated the experimental model in beef cattle with different DMI-inducing changes in BER [6, 23, 42]. In ruminants, the glucose is obtained by ruminal volatile fatty acids and, despite these animals having a low peripheral glucose concentration, glucose acts as the main substrate for cell metabolism [43]. However, by increasing the BER, the high fatty acids (FA) availability stimulated the FA oxidation to become the main substrate for cellular metabolism [44] which allows for higher availability of glucose and subsequently insulin concentrations. A state whereby a normal concentration of insulin induces a decreased biological response in the insulin-sensitive tissues is defined as insulin resistance [43]. Before getting to this metabolic condition, the insulin concentration is increased by pancreatic β cells, promoting hyperinsulinemia [43]. Also, basal hyperinsulinemia in cattle is a result not of a reduced insulin body catabolism, but a higher glucose-induced insulin secretion rate which is potentiated in estrus [45–47]. Evaluating cows with moderated BER, in which DMI was high, Adamiak et al. [6] observed that nutritional plan increased the blood insulin concentration and promoted a hyperinsulinemia condition. Our data corroborate this work suggesting that HBER cows may have a hyperinsulinemia condition due to high insulin levels.

High BER is associated with increased hepatic blood flow and, consequently, steroid metabolism [40, 48]. Despite the evidences that BER influences steroids metabolization, in the present work, differences in BER do not alter E2 and P4 serum concentration. Additionally, P4 concentrations were different between days because the P4 concentration was increased following ovulation, with the presence of an active corpus luteum, which is the main structure responsible for P4 synthesis [49]. Despite we have no data from liver metabolism and clearance, the ovarian hormones serum concentration may have no statistical differences between BER due to higher metabolic liver clearance of E2 and P4 [24, 50]. Thus, our results suggest that the effects observed in the reproductive efficiency are not due to altered reproductive steroid hormones and probably due to other effects caused by the elevated BER such as alterations within the follicular environment.

Cow nutrition influences ovarian follicles’ selection and development, and changing body energy condition may alter the follicular environment and gametic/embryonic quality [7, 51]. The increase in BER by the high DMI promotes lower response to the multiple ovulation protocol, subsequently lower ovulation rates [23, 52] and increased propensity of ovarian cysts [52]. However, it is important to consider that the ovarian response and embryo quality is dependent on the animal’s initial BER. High DMI is beneficial for early embryonic development in animals with low BER values, but the opposite occurs for animals with moderate BER [6]. In this work, the nutritional plan aimed to increase animals’ BER, in this way, our data corroborate with Adamiak et al. [6] which observed that high DMI in moderated BER cows has a negative and cumulative effect on ovarian response and embryo quality. In addition, it is important to mention that embryo recovery rate was calculated taking into account only ovulated animals. That is, HBER animals had lower ovulation rate than MBER animals, and the embryo recovery rate was even lower.

The high DMI and change in BER can alter serum concentration of important hormones. Our data revealed that elevated BER increased serum insulin concentration but did not influence serum concentrations of E2 and P4. In the present work with the aim to evaluate the influences of the changing BER in the follicular environment, the EVs FF size and concentration were evaluated as well as the profile of bovine miRNA in CC and EVs FF. The BER did not induce changes in EVs FF size and concentration; however, the bta-miR-489 decreased in the EVs FF of HBER cows. This miRNA is widely associated with several types of cancer since its function is related to cell proliferation inhibition and apoptosis induction by PI3K/AKT pathway inactivation [53]. One of the actions of miR-489 is to regulate apoptosis by targeting 3’-UTR of IGF-I, which inhibits its expression [54]. Also, glucose fluctuations reduce miR-489 expression [55]. In this way, this miRNA function appear to be related to glucose metabolism. Despite that this study did not evaluate the bta-miR-489 targets and its application on biological pathways. Our data suggests that bta-miR-489 decreased levels are induced by HBER influence on follicular environment, which can be connected with increased levels of serum insulin in HBER cows. Similar studies evaluating EVs FF miRNA content observed metabolic influences on follicular environment. A total of 16 miRNAs in EVs FF were differently expressed in cows with negative and positive energy balance predicting to target biological pathways as TGF-beta signaling, apoptosis, cell cycle and FoxO signaling [56]. In humans, 18 EVs FF miRNAs were associated with high body mass index which are related with PI3K/AKT signaling, adipocytokine signaling, AMPK signaling other important pathways to folicular development [57].

Otherwise, when comparing the miRNA profile of CC and EV FF within treatments we observed that there are several distinct miRNAs among samples, suggesting that BER can influence follicular communication mediated by EVs. It is important to consider that during the estrous cycle, follicles go through different sizes and phases impacting their biochemical characteristic [58]. These follicular changes are highly influenced by the bidirectional communication between follicular cells impacting gamete quality. The EVs within the follicular fluid, are one of the main communication agents, actively interact with granulosa cells and CCs, sending bioactive molecules and, thus, assisting in the oocyte’s process of acquiring competence [13, 15, 59]. In high BER animals, the elevated DMI appears to affect later follicular developmental stages as well as early embryonic development [23, 60], suggesting an influence in communication between follicular compartments as early as 3–6 mm follicular diameter. Based on our data, the functional enrichment analysis for miRNAs differentially expressed between CC and EV FF in each group, we found predicted pathways related to cell proliferation, survival, growth and metabolism, which are biological processes associated with the follicular development. Furthermore, the HBER cows showed higher serum insulin concentration than MBER cows, and also the miRNAs up-regulated in EV FF compared to CC in HBER that are predicted to downregulate the insulin signaling pathway. Cows with extreme BER had significant differences in serum metabolomic profile that may cross the blood follicular barrier influencing the follicle and oocyte quality [61]. This suggest that the elevated serum concentration of insulin in HBER cows may play a role on follicular development, which can be partially mediated by altered miRNAs contents in EVs FF.

We also identified that miRNAs exclusively detected in EV FF compared to CC, are different between MBER and HBER group as well as their predicted biological function. While miRNAs in EVs in MBER group are predicted to regulated pathways as Endocrine resistance, Adherens junction and HIF-1 signaling pathway, the pathways predicted by exclusives EV miRNA in HBER groups are Metabolic pathways, Ras and MAPK signaling pathways. Additionally, the number of miRNAs exclusively or differentially expressed in EVs FF was higher than CC in both groups. It is important to highlight that we used different buffers for miRNAs profile analysis by RT- PCR, being HiSpec for CC and HiFlex for EV FF miScript (miScript II RT kit; QIAGEN). The HiSpec buffer is indicated to evaluate the mature miRNAs, that is, the miRNA that has being processed by DROSHA and DICER and is ready to be loaded into the RISC complex [20, 62, 63]. While, the HiFlex buffer promotes the cDNA conversion of the precursor and mature miRNAs, being able to detected a larger amount of miRNAs, since it also evaluates the miRNAs that have not yet been fully processed and are preferentially secreted by the cells [20, 62, 63]. Additionally, in HBER group, 47.72% (42/88) of the miRNAs are exclusive to EVs FF, while in MBER group the similarity between CC and EVs FF is greater, since only 7.05% (6/79) of the miRNAs are exclusive to EV FF, suggesting an active secretion of molecules by the HBER follicular cells. It is plausible to suppose that it occurs because the EVs FF origin may be from other follicular cell types, such as granulosa cells, which due to the BER effect, may have metabolic alterations. Granulosa cells are influenced by high BER leading to increased apoptosis processes and altered mitochondrial metabolic function [64, 65] suggesting that molecules secreted by them may be different. In this way, the BER can influence indirectly the follicular environment by changing the communication mediated by EVs between the different follicular compartments in 3–6 mm follicles.

## Conclusion

Taken together, our data reveal that changing BER promotes a hyperinsulinemia condition, decreases ovulation rates and decreases embryo recovery rates. Additionally, our data suggests that elevated BER can modify EVs miRNAs contents within follicles between 3–6 mm, which are commonly used for commercial ovum pick-up (OPU). This suggests that in later stages the follicular development and oocyte quality can possibly be altered. Additionally, more studies exploiting the BER effect should be performed in other reproductive compartments, such as the oviduct, the subsequent structure where fertilization and early embryo development takes place. Thus, three important areas of research need to be further evaluated in the future, being the regulation of ovulation, fertilization environment, and early embryo development in high body energy reserve beef cows.

## Supporting information

S1 Fig Reproductive tract collection for embryo, cumulus cells and follicular fluid recovery. **A.** MBER group reproductive tract **B.** HBER group reproductive tract. **C.** Ipsilateral oviduct was dissected for flushing of its contents. **D.** The isthmus portion was flushed with 1xPBS in the ovary-uterus direction. **E.** A 8-cell embryo representative image obtained by magnifying glass. **F.** Ovarian follicles (3–6 mm) from ipsi and contralateral ovaries were aspirated to obtain cumulus cells and follicular fluid for small extracellular vesicles separation and analysis.
(JPG)

S2 Fig Representative image of animals with different body energy reserve. **A.** Moderated body energy reserve group (MBER). **B.** Hight body energy reserve group (HBER).
(JPG)

S3 Fig Follicular fluid extracellular vesicles characterization. **A.** Follicular fluid extracellular vesicles from cows with different body energy reserve analyzed by nanoparticle tracking analysis (NTA). **B.** Extracellular vesicles size and concentration were analyzed by NTA. **C.** Western blotting analysis demonstrates the presence of characteristic vesicles proteins (ALIX and CD9) and absence of cell-specific proteins in follicular fluid vesicle samples (GRP78). The western blot images were cropped for the purpose of this figure. **D.** Transmission electron microscopy images shows the presence of extracellular vesicles in follicular fluid.
(JPG)

S1 Table Ingredients on a dry matter (DM) basis (g/kg) of adaptation diets. (DOCX)

S2 Table Least squares mean, standard error of mean and probability of performance and carcass characteristics of Nellore cows that changed their body energy reserve after the experimental period. (DOCX)

S3 Table Raw cycle threshold levels of the 383 miRNAs profile in cumulus cells (CC) from ipsi and contralateral ovarian follicles (3–6 mm in diameter) from cows with different body energy reserve. (DOCX)

S4 Table Raw cycle threshold levels of the 383 miRNAs profile in follicular fluid extracellular vesicles (FF EVs) from ipsi and contralateral ovarian follicles (3–6 mm in diameter) from cows with different body energy reserve. (DOCX)

S5 Table Normalized data of the 6 exclusives miRNAs detected in follicular fluid extracellular vesicles (EV FF) compared to cumulus cells (CC) from ipsi and contralateral ovarian follicles (3–6 mm in diameter) from cows with moderated body energy reserve (MBER). (DOCX)

S6 Table Normalized data of the 79 miRNAs commonly detected in cumulus cells (CC) and follicular fluid extracellular vesicles (EV FF) from ipsi and contralateral ovarian follicles (3–6 mm in diameter) from cows with moderated body energy reserve (MBER). (DOCX)

S7 Table Normalized data of the 42 exclusives miRNAs detected in follicular fluid extracellular vesicles (EV FF) compared to cumulus cells (CC) from ipsi and contralateral ovarian follicles (3–6 mm in diameter) from cows with high body energy reserve (HBER). (DOCX)

S8 Table Normalized data of the 46 miRNAs commonly detected in cumulus cells (CC) and follicular fluid extracellular vesicles (EV FF) from ipsi and contralateral ovarian follicles (3–6 mm in diameter) from cows with high body energy reserve (HBER). (DOCX)

S9 Table Biological patwhays predicted as modulated by exclusives miRNAs detected in follicular fluid extracellular vesicles (EV FF) compared to cumulus cells (CC) from ipsi and contralateral ovarian follicles (3–6 mm in diameter) from cows with moderated body energy reserve (MBER). (DOCX)

S10 Table Biological patwhays predicted as modulated by miRNAs up regulated in cumulus cells (CC) compared to follicular fluid extracellular vesicles (EV FF) from ipsi and contralateral ovarian follicles (3–6 mm in diameter) from cows with moderated body energy reserve (MBER). (DOCX)

S11 Table Biological patwhays predicted as modulated by exclusives miRNAs detected in follicular fluid extracellular vesicles (EV FF) compared to cumulus cells (CC) from ipsi and contralateral ovarian follicles (3–6 mm in diameter) from cows with high body energy reserve (HBER). (DOCX)

S12 Table Biological patwhays predicted as modulated by miRNAs up regulated in follicular fluid extracellular vesicles (EV FF) compared to cumulus cells (CC) from ipsi and contralateral ovarian follicles (3–6 mm in diameter) from cows with high body energy reserve (HBER). (DOCX)

S1 Raw image (PDF)
