# Supplementary figures and images for: High body energy reserve influences extracellular vesicles miRNA contents within the ovarian follicle
Source: PLoS One. 2023 Jan 10;18(1):e0280195. doi: 10.1371/journal.pone.0280195 (PMC9831338; doi:10.1371/journal.pone.0280195)

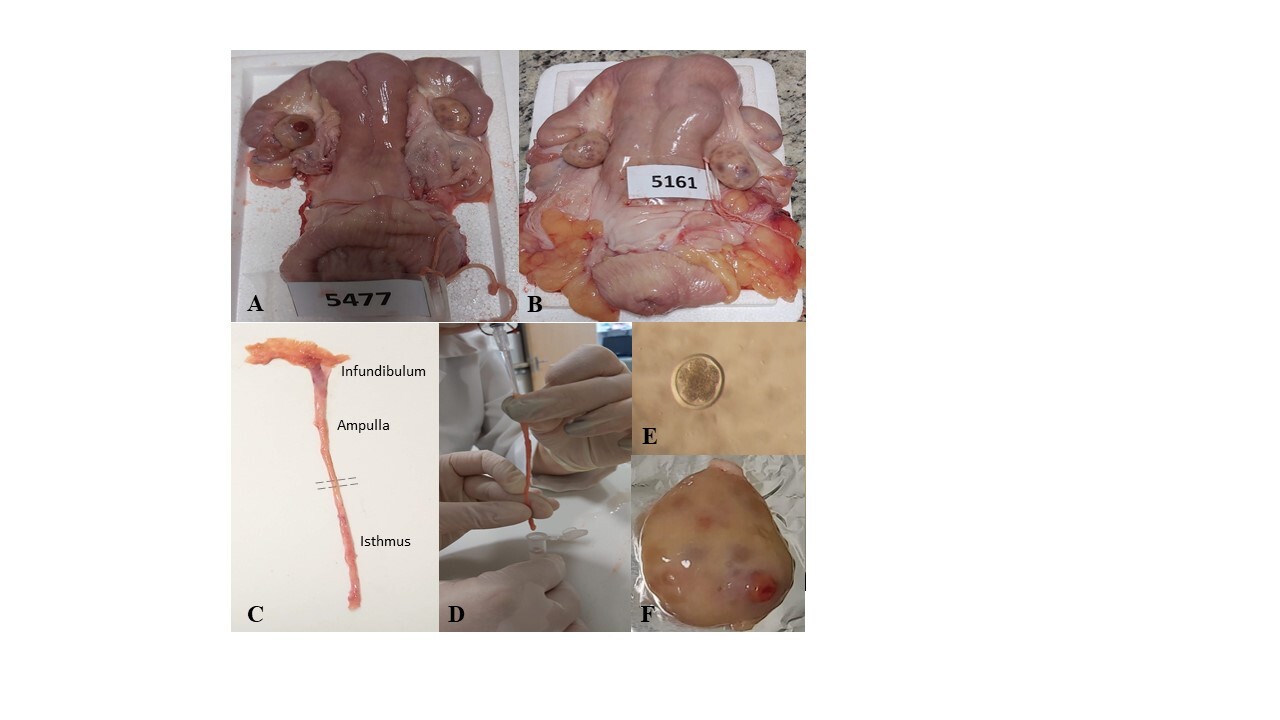

Supplement: S1 Fig — A. MBER group reproductive tract B. HBER group reproductive tract. C. Ipsilateral oviduct was dissected for flushing of its contents. D. The isthmus portion was flushed with 1xPBS in the ovary-uterus direction. E. A 8-cell embryo representative image obtained by magnifying glass. F. Ovarian follicles (3–6 mm) from ipsi and contralateral ovaries were aspirated to obtain cumulus cells and follicular fluid for small extracellular vesicles separation and analysis. (JPG) [file pone.0280195.s001.jpg]

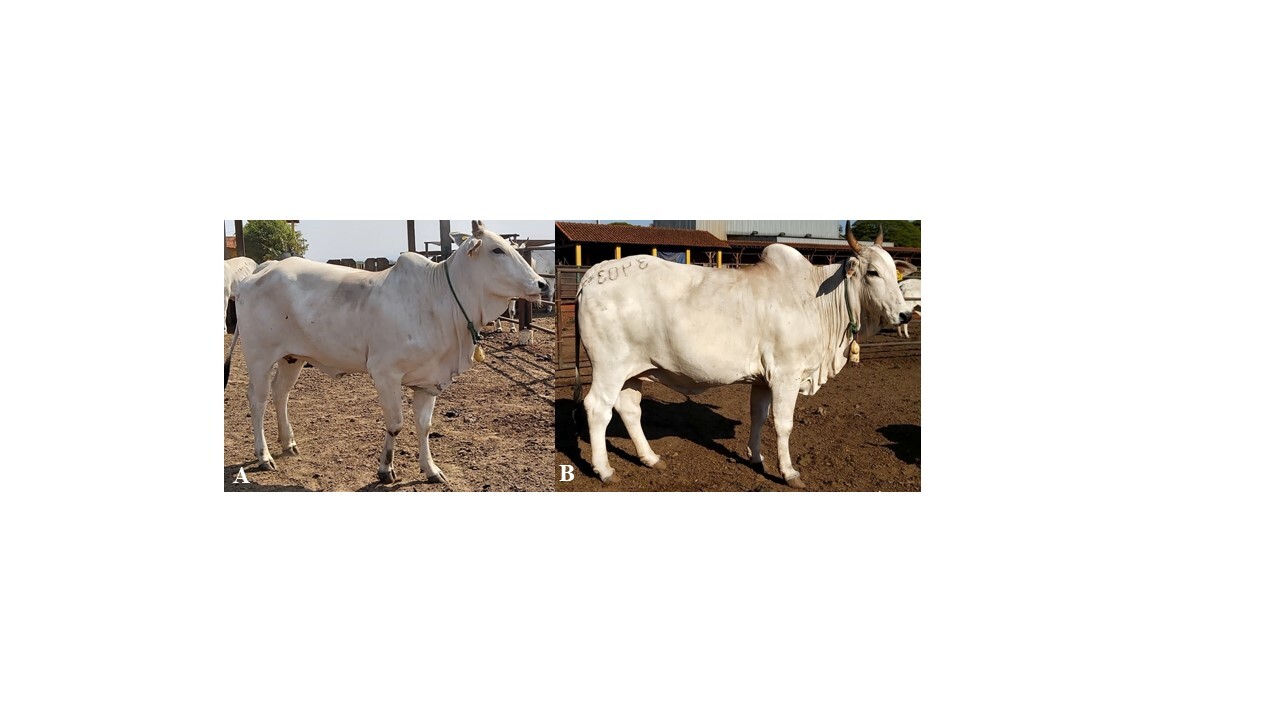

Supplement: S2 Fig — A. Moderated body energy reserve group (MBER). B. Hight body energy reserve group (HBER). (JPG) [file pone.0280195.s002.jpg]

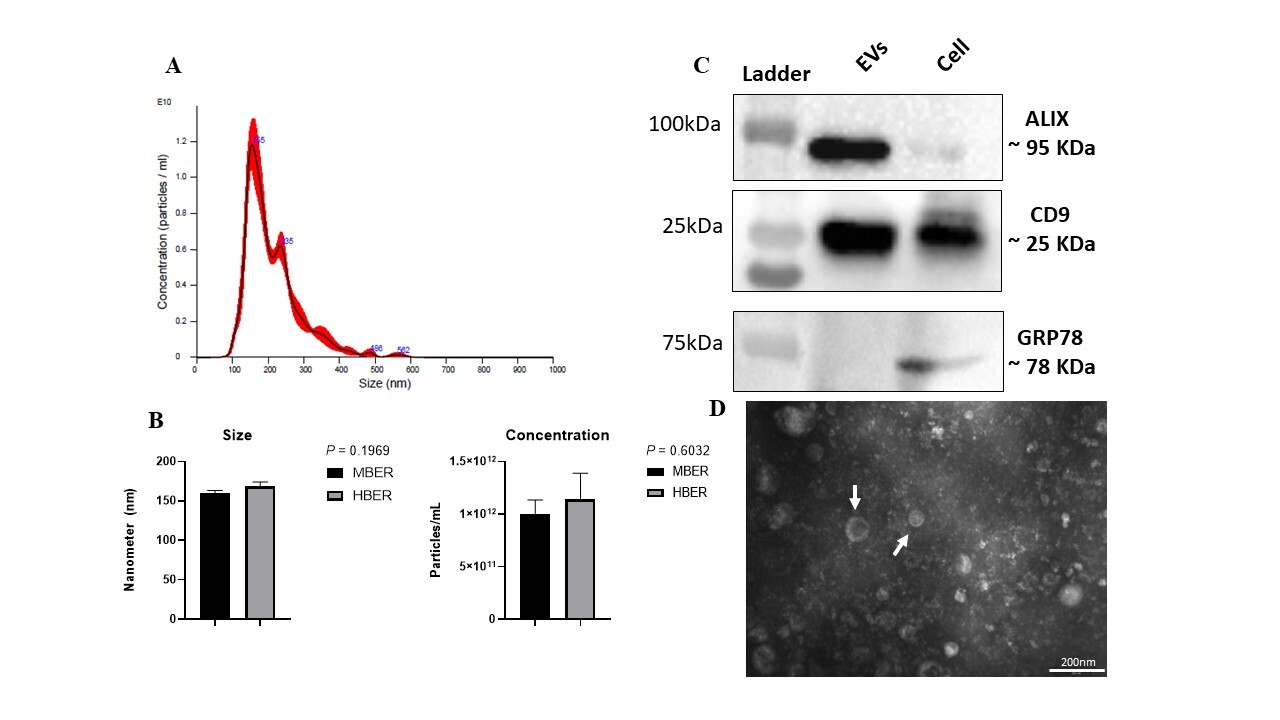

Supplement: S3 Fig — A. Follicular fluid extracellular vesicles from cows with different body energy reserve analyzed by nanoparticle tracking analysis (NTA). B. Extracellular vesicles size and concentration were analyzed by NTA. C. Western blotting analysis demonstrates the presence of characteristic vesicles proteins (ALIX and CD9) and absence of cell-specific proteins in follicular fluid vesicle samples (GRP78). The western blot images were cropped for the purpose of this figure. D. Transmission electron microscopy images shows the presence of extracellular vesicles in follicular fluid. (JPG) [file pone.0280195.s003.jpg]
