# Supplementary material for: High body energy reserve influences extracellular vesicles miRNA contents within the ovarian follicle
Source: PLoS One. 2023 Jan 10;18(1):e0280195. doi: 10.1371/journal.pone.0280195 (PMC9831338; doi:10.1371/journal.pone.0280195)
Supplement: S1 Table — (DOCX) [file pone.0280195.s004.docx]

| **S1 Table.** Ingredients on a dry matter (DM) basis (g/kg) of adaptation diets. | | | |
| --- | --- | --- | --- |
|  | Adaptation diet^1^ (g/kg of DM) | | |
| Ingredients ^2^ | 1 | 2 | 3 |
| Corn silage | 600 | 500 | 550 |
| Finely ground corn | 278.4 | 380 | 470 |
| Soybean meal | 102.4 | 100 | 76.3 |
| Mineral supplement | 5.6 | 6.3 | 6.2 |
| Limestone | 5.4 | 5.6 | 9.7 |
| Urea | 4.5 | 4.4 | 6.9 |
| Salt | 3.5 | 4.4 | 4.4 |
| ^1^Adaptation diet: Adaptation diet 1 with 40:60 concentrate and corn silage ratio; Adaptation diet 2 with 50:50 concentrate and corn silage ratio; Adaptation diet 3 with 58:42 concentrate and corn silage ratio. ^2^Ingredients: Mineral supplement: Ca (208 g/kg); Co (148 mg/kg), Cu (2.7 mg/kg), S (64 g/kg), F (1.6 mg/kg), P (160 g/kg), I (141 mg/kg), Mn (2.2 mg/kg) Se (37 mg/kg), Zn (79.92 mg/kg) and sodium monensin (4000 mg/kg). | | | |
