# Supplementary material for: High body energy reserve influences extracellular vesicles miRNA contents within the ovarian follicle
Source: PLoS One. 2023 Jan 10;18(1):e0280195. doi: 10.1371/journal.pone.0280195 (PMC9831338; doi:10.1371/journal.pone.0280195)
Supplement: S2 Table — (DOCX) [file pone.0280195.s005.docx]

| **Supplementary table 2.** Least squares mean, standard error of mean and probability of performance and carcass characteristics of Nellore cows that changed their body energy reserve after the experimental period. | | | |
| --- | --- | --- | --- |
| **Item** | **Body energy reserve^1^** | | **P- value^2^** |
|  | **MBER** | **HBER** |  |
| Number of animals | 9 | 12 | - |
| Height of withers, cm | 144 ± 0.016 | 145 ± 0.007 | 0.6204 |
| Dry matter intake, kg | 8.20 ± 0.74 | 12.07 ± 0.76 | **<.0001** |
| Average daily gain, kg | 0.41 ± 0.54 | 1.39 ± 0.60 | **<.0001** |
| Carcass weight, kg | 295.82 ± 10.76 | 363.53 ± 16.39 | **0.0020** |
| Carcass yield, % | 58.78 ± 0.47 | 57.31 ± 0.5 | **0.0331** |
| ^1^Body energy reserve: MBER: Cows with moderated body energy reserve; HBER: Cows with high body energy reserve; ^2^P-value: P value between animals with different body energy reserve. | | | |
