# Supplementary material for: High body energy reserve influences extracellular vesicles miRNA contents within the ovarian follicle
Source: PLoS One. 2023 Jan 10;18(1):e0280195. doi: 10.1371/journal.pone.0280195 (PMC9831338; doi:10.1371/journal.pone.0280195)
Supplement: S3 Table — (DOCX) [file pone.0280195.s006.docx]

| **Supplementary table 3.** Raw cycle threshold levels of the 383 miRNAs profile in cumulus cells (CC) from ipsi and contralateral ovarian follicles (3-6 mm in diameter) from cows with different body energy reserve. | | | | | | | | | | | | |
| --- | --- | --- | --- | --- | --- | --- | --- | --- | --- | --- | --- | --- |
| **MiRNAs** | **Body energy reserve^1^** | | | | | | | | | | | |
|  | **MBER** | | | | | | | | **HBER** | | | |
|  | **1** | **2** | **3** | **4** | **5** | **6** | **7** | **8** | **1** | **2** | **3** | **4** |
| bta-let-7a-3p | 32.6329 | . | . | . | . | . | . | . | . | . | . | . |
| bta-miR-103 | 33.4939 | . | . | . | . | . | 34.1132 | 36.0767 | . | . | . | . |
| bta-let-7a-5p | 27.7830 | 35.5376 | 34.8821 | . | 29.1532 | 28.4824 | . | 25.4194 | 34.8821 | 34.5822 | 29.8289 | . |
| bta-miR-105a | . | . | . | . | . | . | 33.1376 | . | . | . | . | . |
| bta-let-7b | 30.6383 | . | 35.0286 | . | 30.6173 | 30.3857 | . | 26.7068 | 35.0286 | . | 30.3539 | . |
| bta-miR-105b | . | . | . | . | . | . | . | 36.5265 | . | . | . | . |
| bta-let-7c | 28.3012 | 33.3092 | 34.3397 | . | 29.0178 | 28.6414 | 34.4343 | 25.6382 | 34.3397 | 35.0800 | 29.3478 | 34.9363 |
| bta-miR-106a | 33.2136 | . | . | . | 34.5924 | 36.4703 | 35.3861 | 32.2902 | . | 35.4608 | . | . |
| bta-let-7d | 29.4991 | 36.9923 | . | . | 30.7635 | 29.9570 | . | 26.8458 | . | . | 32.0303 | . |
| bta-miR-106b | 35.4937 | . | . | . | 34.9646 | . | . | 35.4235 | . | . | 34.3796 | . |
| bta-let-7e | 28.2844 | 33.9702 | 32.4129 | 34.3867 | 28.4557 | 27.0000 | 33.3462 | 25.0261 | 32.4129 | . | 28.4566 | . |
| bta-miR-107 | . | . | . | . | . | . | . | . | . | . | . | . |
| bta-let-7f | 28.0775 | . | . | . | 31.7152 | 30.6277 | . | 27.2196 | . | 34.2189 | 31.5689 | . |
| bta-miR-10a | 31.8335 | . | . | . | . | . | . | . | . | . | . | 34.6471 |
| bta-let-7g | 28.7382 | 34.3414 | . | . | 31.7306 | 34.8084 | . | 28.8894 | . | . | 32.5109 | . |
| bta-miR-10b | 31.5248 | . | . | . | . | . | 35.1241 | 33.1489 | . | . | 35.4336 | . |
| bta-let-7i | 29.6923 | . | . | . | . | . | . | 30.3218 | . | . | . | . |
| bta-miR-122 | . | . | . | . | . | . | . | . | . | . | 34.9644 | . |
| bta-miR-1 | . | . | . | . | . | . | . | . | . | . | . | . |
| bta-miR-124a | . | . | . | . | . | . | . | . | . | . | . | 35.9199 |
| bta-miR-100 | 34.3975 | . | 33.9009 | . | 36.6398 | . | 34.0635 | . | 33.9009 | . | . | . |
| bta-miR-124b | . | . | . | . | . | . | . | 36.5840 | . | . | . | . |
| bta-miR-101 | . | . | . | . | 34.4550 | . | . | . | . | . | . | . |
| bta-miR-125a | 28.7070 | . | 35.6764 | 34.8620 | 33.8154 | 31.6039 | 34.9567 | 29.2240 | 35.6764 | . | 32.9381 | . |
| bta-miR-125b | 31.4882 | . | 36.9339 | . | 36.4632 | 33.0967 | . | 31.9554 | 36.9339 | 34.2379 | 34.9169 | . |
| bta-miR-133b | . | . | . | . | . | . | . | . | . | . | . | . |
| bta-miR-126-3p | . | 34.7000 | . | 33.5459 | . | . | 31.3812 | . | . | . | 32.8437 | 32.6703 |
| bta-miR-133c | 34.6735 | . | . | . | . | . | 31.8928 | . | . | . | 32.8993 | . |
| bta-miR-126-5p | . | . | . | 36.0299 | . | . | 30.8388 | . | . | . | 32.5819 | 32.7644 |
| bta-miR-134 | . | . | . | 32.9150 | . | . | 30.1930 | . | . | . | 34.4423 | 31.7897 |
| bta-miR-127 | 35.7024 | 34.8754 | 34.2865 | 33.4015 | 33.9272 | 32.8803 | 32.9846 | 33.1199 | 34.2865 | . | 34.8852 | 33.4915 |
| bta-miR-135a | . | . | . | . | . | . | . | . | . | . | . | . |
| bta-miR-128 | . | . | . | . | . | . | . | 33.7580 | . | . | 34.7147 | . |
| bta-miR-135b | . | . | . | . | . | . | . | . | . | . | . | . |
| bta-miR-129 | . | . | . | . | 33.9088 | . | . | 35.1693 | . | . | . | . |
| bta-miR-136 | . | . | . | . | . | . | . | . | . | . | . | . |
| bta-miR-129-3p | 36.3606 | . | . | . | . | . | 33.2454 | . | . | . | . | 35.5468 |
| bta-miR-137 | . | . | . | . | . | . | 35.2344 | . | . | . | . | . |
| bta-miR-129-5p | . | . | . | . | . | . | . | 35.9813 | . | . | . | . |
| bta-miR-138 | . | . | . | 31.7937 | . | . | 29.8171 | 31.7289 | . | . | 35.2409 | 30.1953 |
| bta-miR-130a | 35.0502 | 34.4067 | 31.8284 | 32.9105 | 34.7652 | 32.2672 | 31.8123 | 34.8558 | 31.8284 | . | 33.5480 | 32.9560 |
| bta-miR-139 | . | . | . | . | . | . | 33.4570 | . | . | . | . | . |
| bta-miR-130b | 31.7478 | 33.1327 | 31.8508 | 31.7928 | 33.9760 | 32.1363 | 31.3036 | 33.1302 | 31.8508 | 35.9067 | 32.5855 | 31.7114 |
| bta-miR-140 | 35.9445 | . | . | . | . | . | 33.9359 | . | . | . | . | 34.1767 |
| bta-miR-132 | 32.7076 | . | . | 33.7561 | . | . | 30.7109 | 34.1514 | . | . | 33.9945 | 33.9350 |
| bta-miR-141 | . | . | . | . | . | . | 33.0067 | . | . | . | 34.2814 | 35.3385 |
| bta-miR-133a | . | . | . | . | . | 36.4311 | 29.8702 | . | . | 22.8246 | 32.5369 | 33.9379 |
| bta-miR-142-3p | . | . | . | . | . | . | . | . | . | . | 34.8907 | 34.8048 |
| bta-miR-142-5p | . | . | . | 35.2976 | . | . | 35.0368 | . | . | . | 35.0325 | . |
| bta-miR-151-3p | . | . | . | 36.7687 | . | 35.8585 | 34.7198 | 33.0143 | . | . | . | . |
| bta-miR-143 | 33.9600 | 34.7640 | . | 34.8022 | 34.9841 | 33.8794 | 31.8105 | 35.9016 | . | . | 32.9025 | 33.2229 |
| bta-miR-151-5p | 33.5249 | . | . | . | . | 36.7780 | . | 32.9893 | . | 34.9583 | . | . |
| bta-miR-144 | . | . | . | . | . | . | . | . | . | . | . | . |
| bta-miR-152 | 36.7749 | . | 36.8008 | 33.2544 | . | . | 31.8320 | . | 36.8008 | . | 33.1764 | . |
| bta-miR-145 | . | . | . | 33.8873 | 34.2177 | . | 31.8903 | 33.4795 | . | . | 36.9522 | 32.6011 |
| bta-miR-153 | . | . | . | . | . | . | . | . | . | . | . | . |
| bta-miR-146a | . | . | . | . | . | . | 34.9847 | . | . | . | 34.5914 | . |
| bta-miR-154a | . | . | . | 36.2968 | . | 35.5757 | 32.2864 | . | . | . | . | 34.1084 |
| bta-miR-146b | . | 34.2215 | . | 35.0519 | . | . | 34.8799 | . | . | . | . | 34.6747 |
| bta-miR-154b | . | 34.7742 | 32.8739 | 30.3812 | 33.9249 | 33.3504 | 29.1363 | 31.7179 | 32.8739 | . | 31.9521 | 30.3117 |
| bta-miR-147 | . | . | . | . | . | . | . | 35.3285 | . | . | . | . |
| bta-miR-154c | . | 36.3512 | . | . | 36.8795 | . | 36.6252 | . | . | . | . | . |
| bta-miR-148a | 32.8794 | . | . | . | 34.1772 | . | . | . | . | 34.2057 | . | . |
| bta-miR-155 | 32.8174 | . | . | . | 32.5614 | 32.8736 | 33.3902 | 33.9497 | . | . | 33.6392 | 34.2225 |
| bta-miR-148b | 31.8361 | . | . | . | . | . | . | 36.2579 | . | 34.2469 | . | . |
| bta-miR-15a | 34.6343 | . | . | . | . | . | 35.0208 | . | . | . | . | . |
| bta-miR-149-3p | 34.3217 | . | . | 34.5023 | 35.2679 | 33.0436 | 32.3272 | 32.7985 | . | . | 32.9724 | 32.6484 |
| bta-miR-15b | 28.7549 | . | 33.9609 | . | 33.8676 | 30.6392 | . | 29.7108 | 33.9609 | . | 32.8569 | . |
| bta-miR-149-5p | . | . | . | . | . | 36.0016 | 32.7299 | 34.3492 | . | . | 33.3838 | 32.7677 |
| bta-miR-16a | 29.6701 | . | . | . | . | 33.1408 | 32.8766 | 31.6752 | . | 33.5683 | . | . |
| bta-miR-150 | . | . | . | . | . | . | 32.7471 | . | . | . | 34.4546 | 34.9615 |
| bta-miR-16b | 29.8265 | . | . | . | 33.4366 | 33.1997 | 32.5006 | 30.7786 | . | 33.9599 | 32.8449 | 32.9321 |
| bta-miR-17-3p | . | . | . | . | . | . | . | . | . | . | . | . |
| bta-miR-188 | . | . | . | . | . | . | 32.9035 | 35.3687 | . | . | . | . |
| bta-miR-17-5p | 34.9310 | . | . | . | . | . | 34.2054 | 32.9785 | . | . | . | 36.5868 |
| bta-miR-18a | 34.4806 | . | . | . | . | . | . | 33.3318 | . | . | . | . |
| bta-miR-181a | 34.8406 | . | . | . | . | . | . | . | . | 35.1678 | . | . |
| bta-miR-18b | . | . | . | . | . | . | . | . | . | . | . | . |
| bta-miR-181b | . | . | . | . | . | 33.6750 | . | 34.9148 | . | . | . | . |
| bta-miR-190a | . | . | . | . | . | . | . | . | . | . | . | . |
| bta-miR-181c | . | . | . | . | . | . | . | . | . | . | 36.6183 | . |
| bta-miR-190b | 36.6755 | . | . | . | . | . | 34.5340 | 33.1601 | . | . | 34.4527 | . |
| bta-miR-181d | 33.5128 | 35.3660 | . | 34.9862 | 34.9305 | 35.0255 | 32.7695 | 33.7460 | . | . | . | 33.7427 |
| bta-miR-191 | 31.6633 | 19.6404 | 27.8390 | 18.2415 | 32.7027 | 33.3956 | 31.9914 | 31.4216 | 27.8390 | 20.0441 | 32.8450 | 21.8092 |
| bta-miR-182 | . | . | . | . | . | . | 33.2708 | . | . | . | . | 34.2894 |
| bta-miR-192 | 34.9210 | . | . | 36.8534 | . | 33.8752 | . | . | . | . | . | . |
| bta-miR-183 | . | . | . | . | . | . | 32.4643 | . | . | . | 34.8324 | 36.5417 |
| bta-miR-193a | . | . | . | . | . | . | . | . | . | . | . | . |
| bta-miR-184 | . | . | . | . | . | . | 33.3915 | . | . | . | 33.5950 | 34.8707 |
| bta-miR-193a-3p | . | . | . | . | . | . | . | . | . | . | 35.1697 | . |
| bta-miR-185 | 35.2934 | . | . | . | . | . | . | 35.3009 | . | . | . | . |
| bta-miR-193a-5p | 36.9167 | . | 36.7786 | . | 33.9348 | 31.9433 | . | 31.6654 | 36.7786 | . | 34.0928 | . |
| bta-miR-186 | . | . | . | . | . | . | 32.0485 | 32.8926 | . | . | 33.7941 | 32.9233 |
| bta-miR-193b | . | . | . | . | . | . | . | . | . | . | . | . |
| bta-miR-187 | . | 32.0092 | . | . | . | . | 31.4952 | 33.8485 | . | . | . | 32.8885 |
| bta-miR-194 | . | . | . | . | . | . | . | . | . | . | . | . |
| bta-miR-195 | 30.4225 | 33.9622 | 35.9576 | 35.9908 | 34.4141 | 32.5607 | 31.9836 | 30.3539 | 35.9576 | . | 34.3291 | 33.9686 |
| bta-miR-200c | . | . | . | . | . | . | 33.6991 | . | . | . | 34.2661 | . |
| bta-miR-196a | 34.8447 | . | . | 34.9120 | 33.8648 | . | 32.0356 | 32.8575 | . | . | 33.6075 | 33.0888 |
| bta-miR-202 | 28.1818 | . | . | . | . | 35.2635 | . | 30.4398 | . | 32.5535 | 30.8038 | . |
| bta-miR-196b | . | . | . | 36.2879 | . | . | 35.7364 | 33.5800 | . | . | 34.8288 | 33.7672 |
| bta-miR-204 | . | . | . | 36.6348 | . | 36.6706 | 31.5981 | . | . | . | 32.2138 | 32.9583 |
| bta-miR-197 | 33.9686 | . | . | . | 30.3852 | 28.5821 | 30.1886 | 26.9685 | . | . | 28.8215 | 31.8557 |
| bta-miR-205 | . | . | . | . | . | . | 32.9755 | 35.3986 | . | . | 31.8619 | 34.9110 |
| bta-miR-199a-3p | 34.7238 | . | . | . | . | . | . | . | . | . | . | . |
| bta-miR-206 | . | . | . | 35.5308 | 34.3179 | 34.6096 | 31.7284 | . | . | . | 31.7719 | 32.1446 |
| bta-miR-199a-5p | . | . | . | . | . | . | . | . | . | . | . | . |
| bta-miR-208a | . | . | . | . | . | . | . | . | . | . | . | . |
| bta-miR-199b | . | . | . | . | . | . | 35.7784 | . | . | . | . | . |
| bta-miR-208b | . | . | . | . | . | . | . | . | . | . | . | . |
| bta-miR-199c | . | 34.2018 | . | . | . | . | 31.7519 | . | . | . | 31.5969 | 34.1195 |
| bta-miR-20a | 31.6567 | . | . | 34.9325 | 34.4801 | . | 33.7804 | 32.1166 | . | 35.4886 | 33.5269 | 34.7654 |
| bta-miR-19a | . | . | . | . | . | . | . | . | . | 35.3081 | 35.6543 | . |
| bta-miR-20b | 34.8625 | . | . | . | . | . | 36.8952 | 34.8504 | . | . | . | 34.9714 |
| bta-miR-19b | 34.7653 | . | . | . | 34.5400 | . | . | . | . | 35.0393 | . | . |
| bta-miR-21-3p | . | . | . | . | 35.1695 | 35.0111 | . | . | . | . | . | . |
| bta-miR-200a | . | . | . | . | . | . | . | . | . | . | . | . |
| bta-miR-21-5p | 31.6939 | . | . | . | 33.8697 | . | . | 33.2655 | . | 34.0121 | . | . |
| bta-miR-200b | 34.2378 | . | . | . | . | . | 32.9298 | 35.6406 | . | . | 34.4080 | . |
| bta-miR-210 | 33.6049 | . | . | . | . | 34.9643 | . | 34.1871 | . | . | 33.8143 | 34.2843 |
| bta-miR-211 | . | . | . | 36.3571 | . | . | 32.0915 | . | . | . | 32.7494 | 33.1448 |
| bta-miR-22-5p | 34.5064 | . | . | . | . | . | . | . | . | . | . | . |
| bta-miR-212 | . | . | . | . | . | . | . | 33.6057 | . | . | . | . |
| bta-miR-221 | 35.1105 | . | . | . | . | 36.8768 | . | . | . | . | . | . |
| bta-miR-214 | 35.6785 | . | . | . | 35.2427 | 33.9840 | 36.6934 | 36.3181 | . | . | . | 35.3230 |
| bta-miR-222 | . | . | . | . | . | . | 32.7836 | 35.1659 | . | . | 32.8619 | . |
| bta-miR-215 | 34.8993 | 34.8933 | . | . | . | . | 33.3926 | . | . | . | 35.6990 | 35.2757 |
| bta-miR-223 | . | . | . | 36.8756 | . | . | 34.0398 | . | . | . | 33.8812 | 35.9726 |
| bta-miR-216a | . | . | . | . | . | . | . | . | . | . | . | . |
| bta-miR-224 | 32.8857 | . | . | . | . | . | 34.9315 | 34.7126 | . | . | . | . |
| bta-miR-216b | . | . | . | . | . | 33.8988 | . | 35.9551 | . | . | . | . |
| bta-miR-23a | 29.8475 | . | . | 34.3461 | 35.2246 | 32.8541 | 33.9346 | 32.7422 | . | . | 33.9055 | . |
| bta-miR-217 | . | . | . | . | . | . | . | . | . | . | . | . |
| bta-miR-23b-3p | 30.3252 | . | . | . | 34.8386 | 32.5033 | 32.9699 | 32.1203 | . | 35.4091 | 33.7349 | . |
| bta-miR-218 | . | . | . | . | . | . | 35.4427 | 35.4001 | . | . | . | . |
| bta-miR-23b-5p | 34.9656 | . | . | 34.3006 | . | 35.7368 | . | . | . | . | . | . |
| bta-miR-219 | 36.1105 | 36.5761 | 35.9463 | . | 36.4318 | . | 31.6548 | 35.3552 | 35.9463 | . | 31.3967 | 33.6446 |
| bta-miR-24 | . | 17.8245 | . | . | . | . | . | . | . | 18.3596 | . | . |
| bta-miR-219-3p | . | . | . | . | 36.6639 | 35.0375 | 32.8627 | 35.6858 | . | . | 33.9014 | . |
| bta-miR-24-3p | 33.8440 | . | . | . | . | 36.6923 | . | 32.9922 | . | . | 34.9402 | . |
| bta-miR-219-5p | . | . | . | . | . | . | . | . | . | . | . | . |
| bta-miR-25 | 29.7013 | . | . | 36.9239 | . | 31.9729 | . | 30.4763 | . | 35.1271 | . | . |
| bta-miR-22-3p | 2.1736 | 2.3081 | 2.3994 | 2.5749 | 2.4831 | 2.5526 | 2.6351 | 2.3036 | 2.3994 | 2.6994 | 3.0553 | 2.4952 |
| bta-miR-26a | 28.4467 | . | . | 33.9430 | 33.2769 | 32.8148 | 32.2940 | 30.8431 | . | 32.8127 | 32.6566 | 32.8577 |
| bta-miR-26b | 29.7272 | . | . | . | 33.7686 | 35.0710 | . | 32.9827 | . | 35.5273 | . | . |
| bta-miR-29d-3p | . | . | . | . | . | . | . | . | . | . | . | . |
| bta-miR-26c | . | . | . | . | . | . | . | . | . | . | . | . |
| bta-miR-29d-5p | . | . | 34.9245 | . | . | . | . | . | 34.9245 | . | . | . |
| bta-miR-27a-3p | 34.6542 | . | . | . | 36.2073 | 36.1345 | 31.1250 | . | . | . | 32.8999 | 33.6042 |
| bta-miR-29e | . | . | . | . | . | . | . | . | . | . | . | . |
| bta-miR-27a-5p | 9.4657 | 9.2365 | 8.1142 | 8.5150 | 9.1427 | 35.6654 | . | 7.3364 | 8.1142 | 8.7058 | 9.5688 | 10.1206 |
| bta-miR-301a | . | . | . | . | . | . | . | . | . | . | . | 34.7270 |
| bta-miR-27b | 33.4357 | . | . | . | . | . | 33.4395 | 33.9975 | . | . | 32.3044 | 34.3072 |
| bta-miR-301b | . | . | . | . | . | . | 35.8340 | . | . | . | 35.5925 | . |
| bta-miR-28 | . | . | . | . | . | . | 33.9263 | . | . | . | . | . |
| bta-miR-302a | . | . | . | . | 35.8240 | . | . | . | . | . | 34.9293 | . |
| bta-miR-296-3p | 32.7752 | 33.9950 | . | 35.4993 | 34.5661 | 33.3140 | 35.3540 | 30.9305 | . | . | 33.8311 | 36.4068 |
| bta-miR-302b | . | . | . | . | . | . | . | . | . | . | 34.4227 | 35.8908 |
| bta-miR-296-5p | 35.7104 | . | 36.7894 | . | . | 35.2243 | 32.9498 | 33.9362 | 36.7894 | . | 31.8317 | 32.8323 |
| bta-miR-302c | . | . | . | . | . | . | 33.9126 | . | . | . | . | 35.6125 |
| bta-miR-299 | . | . | . | . | . | . | . | . | . | . | . | . |
| bta-miR-302d | . | . | . | 34.4043 | . | . | . | . | . | . | . | . |
| bta-miR-29a | 32.0420 | . | . | . | . | 33.8299 | . | . | . | 33.8721 | . | . |
| bta-miR-3064 | . | . | . | . | . | . | . | . | . | . | . | . |
| bta-miR-29b | 34.5445 | . | . | . | . | . | . | . | . | . | . | . |
| bta-miR-30a-5p | 32.6045 | . | . | 34.4104 | . | 36.8179 | 35.0071 | 32.0031 | . | 35.6487 | 36.4727 | . |
| bta-miR-29c | 31.1626 | . | . | . | . | 34.8972 | . | 32.7699 | . | . | 34.7598 | . |
| bta-miR-30b-3p | . | . | . | . | 34.6732 | . | . | . | . | . | . | . |
| bta-miR-30b-5p | 32.8332 | . | . | 34.9363 | . | 35.1110 | 32.8819 | 33.2177 | . | . | 34.1697 | 34.9144 |
| bta-miR-328 | 34.1796 | . | . | . | . | 33.3191 | 34.0238 | . | . | . | 35.0194 | . |
| bta-miR-30c | 30.2571 | . | . | . | . | 33.6058 | 32.4000 | 31.1427 | . | 33.5641 | 32.8968 | 34.4084 |
| bta-miR-329a | . | . | . | . | . | . | . | . | . | . | . | . |
| bta-miR-30d | 33.9221 | . | . | . | . | . | 34.8932 | 32.5995 | . | 35.9774 | 34.9089 | . |
| bta-miR-329b | . | . | . | . | . | . | . | . | . | . | . | . |
| bta-miR-30e-5p | 35.1849 | . | . | . | . | . | 34.9262 | 33.0576 | . | 35.0747 | 34.0557 | . |
| bta-miR-330 | . | . | . | . | . | 36.2217 | . | 33.5945 | . | . | 35.0027 | . |
| bta-miR-30f | 32.0531 | . | . | 33.8138 | . | 33.4080 | 35.1965 | 33.1698 | . | . | 34.9343 | . |
| bta-miR-331-3p | . | . | . | . | . | 35.3639 | . | 33.4008 | . | . | . | . |
| bta-miR-31 | 30.4427 | . | . | 36.8190 | 33.4756 | 31.7775 | . | 30.0938 | . | 33.9760 | 33.8044 | . |
| bta-miR-331-5p | . | . | . | 34.5732 | . | 35.2996 | 31.8985 | . | . | . | 31.4883 | 32.5107 |
| bta-miR-32 | . | . | . | . | . | . | . | . | . | . | . | . |
| bta-miR-335 | 33.9707 | . | . | 36.0278 | 34.6767 | . | . | . | . | . | 36.5548 | . |
| bta-miR-320a | 31.8904 | 36.2960 | 34.6620 | . | 31.4937 | 31.8546 | 34.4136 | 28.4305 | 34.6620 | . | 31.0635 | 33.8862 |
| bta-miR-338 | . | . | . | . | . | . | . | . | . | . | . | . |
| bta-miR-320b | . | . | . | . | . | 34.6263 | 32.8969 | 34.7941 | . | . | 34.1305 | 33.9190 |
| bta-miR-339a | . | . | . | 33.6918 | . | . | . | 32.5040 | . | . | . | 34.6318 |
| bta-miR-323 | 19.3785 | 17.1192 | 15.8844 | 15.7498 | 19.4385 | 17.2737 | 17.6686 | 20.6150 | 15.8844 | 21.6157 | 18.7191 | 17.7376 |
| bta-miR-339b | 33.0316 | . | . | 33.5172 | 31.5418 | 35.1416 | 32.0415 | 31.1874 | . | 17.1678 | 31.9507 | 33.2442 |
| bta-miR-324 | . | . | . | . | . | . | 32.7139 | . | . | . | 34.9700 | 33.9812 |
| bta-miR-33a | . | . | . | . | . | . | . | . | . | . | . | . |
| bta-miR-326 | . | . | . | . | 35.7449 | 34.9171 | 35.1819 | . | . | . | . | . |
| bta-miR-33b | . | 36.4775 | . | 36.0681 | 34.8983 | 34.2320 | 34.8549 | 35.4230 | . | . | . | 35.0411 |
| bta-miR-340 | . | . | . | . | . | . | 33.8994 | 33.1944 | . | . | 31.9905 | . |
| bta-miR-365-3p | 30.8804 | . | . | . | . | . | . | 32.3892 | . | . | . | . |
| bta-miR-342 | 33.9694 | . | . | 34.9936 | . | 32.9053 | 33.6413 | 31.6317 | . | . | 33.3334 | 35.4356 |
| bta-miR-365-5p | . | . | . | . | 36.8022 | 34.9886 | . | 34.9077 | . | . | 35.0218 | 35.4840 |
| bta-miR-345-3p | . | . | . | . | . | . | . | . | . | . | . | . |
| bta-miR-367 | . | . | . | . | . | . | 36.2053 | . | . | . | . | . |
| bta-miR-345-5p | . | . | . | . | . | . | 33.8552 | . | . | . | 33.9391 | 33.9637 |
| bta-miR-369-3p | . | . | . | . | . | . | . | . | . | . | . | . |
| bta-miR-346 | . | . | . | . | 35.3747 | 36.6267 | 32.9444 | 32.8427 | . | . | 33.2539 | . |
| bta-miR-369-5p | . | . | . | . | . | . | 33.3732 | 35.8516 | . | . | 33.2680 | 34.4616 |
| bta-miR-34a | . | . | . | . | 35.0421 | 35.2769 | 34.5280 | 32.0705 | . | . | . | 34.6651 |
| bta-miR-370 | . | 35.7506 | . | 34.6013 | 33.9415 | 32.1730 | 34.9481 | 33.0144 | . | 35.4226 | 33.8910 | . |
| bta-miR-34b | . | . | . | . | . | . | . | . | . | . | . | . |
| bta-miR-371 | . | 35.4800 | . | 34.9024 | . | . | . | . | . | . | 36.7333 | 34.9232 |
| bta-miR-34c | . | . | . | . | . | . | . | . | . | . | . | . |
| bta-miR-374a | 34.8218 | . | . | . | . | . | . | . | . | . | . | . |
| bta-miR-361 | 31.9101 | . | . | . | 33.9990 | 32.4140 | . | 31.4354 | . | . | 34.1427 | . |
| bta-miR-374b | 31.6008 | . | . | . | . | . | 34.6589 | . | . | . | . | . |
| bta-miR-362-3p | . | . | . | . | . | . | 34.9182 | . | . | . | . | . |
| bta-miR-375 | . | 34.7678 | 32.9869 | 30.6016 | 34.4514 | 31.7544 | 30.6241 | . | 32.9869 | . | 30.7844 | 29.7425 |
| bta-miR-362-5p | . | . | . | . | . | 36.0219 | . | 34.8051 | . | . | . | . |
| bta-miR-376a | . | . | . | . | . | . | 34.0018 | 36.9730 | . | . | 31.9728 | . |
| bta-miR-363 | . | . | . | . | . | . | . | . | . | . | 33.8912 | . |
| bta-miR-376b | . | . | . | . | . | . | . | . | . | . | . | . |
| bta-miR-376c | . | . | . | . | . | . | . | . | . | . | . | . |
| bta-miR-382 | 36.5578 | 34.9303 | 36.1682 | 32.7086 | 33.9920 | 34.4666 | 31.7033 | 33.0485 | 36.1682 | 36.3061 | 33.0123 | 33.1904 |
| bta-miR-376d | . | . | . | . | . | . | . | . | . | . | . | . |
| bta-miR-383 | . | . | . | 32.9275 | . | . | 33.2008 | . | . | . | 36.8510 | . |
| bta-miR-376e | . | . | . | . | . | . | . | . | . | . | . | . |
| bta-miR-409a | . | . | . | . | . | . | . | . | . | . | . | . |
| bta-miR-377 | . | . | . | . | . | . | . | . | . | . | . | . |
| bta-miR-409b | . | . | . | . | . | . | . | . | . | . | . | 34.9559 |
| bta-miR-378 | . | . | . | . | 34.0453 | 33.4267 | 31.7683 | . | . | . | 32.6782 | 32.6360 |
| bta-miR-410 | . | . | . | . | . | . | 35.0093 | . | . | . | . | . |
| bta-miR-378b | . | . | . | 34.9327 | . | . | 32.7503 | 34.6895 | . | . | 32.1304 | 32.5901 |
| bta-miR-411a | . | 35.9600 | 36.8022 | 34.4003 | 34.6577 | 35.0102 | 32.2470 | 35.6020 | 36.8022 | . | 33.1828 | 32.8486 |
| bta-miR-378c | . | . | . | 36.0124 | . | 35.8411 | 36.5963 | 34.7474 | . | 34.9325 | . | . |
| bta-miR-411b | . | . | 34.5095 | 35.2848 | 35.5426 | 33.7818 | 34.1531 | . | 34.5095 | 23.6390 | 35.1951 | 34.3176 |
| bta-miR-378d | . | 35.9298 | . | 32.8466 | . | 34.9704 | 36.8377 | . | . | . | 35.0205 | 33.6833 |
| bta-miR-411c-3p | . | . | . | . | . | . | . | . | . | . | . | . |
| bta-miR-379 | . | . | . | . | . | . | . | . | . | . | . | . |
| bta-miR-411c-5p | . | . | . | . | . | . | . | . | . | . | . | . |
| bta-miR-380-3p | . | . | . | . | . | . | 33.8743 | 35.7677 | . | . | . | 35.7453 |
| bta-miR-412 | . | . | . | . | . | . | . | . | . | . | . | . |
| bta-miR-380-5p | . | 35.9054 | . | 34.0579 | . | . | 31.8243 | . | . | 34.2154 | 31.4519 | 32.3943 |
| bta-miR-421 | 33.7388 | 34.4778 | 32.6496 | 34.6022 | 31.9194 | . | 31.3350 | 35.1485 | 32.6496 | 34.7893 | 31.8333 | 33.8691 |
| bta-miR-381 | . | . | . | . | . | . | . | . | . | . | 35.1595 | . |
| bta-miR-423-3p | 35.9268 | . | . | . | . | 35.7211 | . | 32.8085 | . | . | . | . |
| bta-miR-423-5p | 31.7584 | . | . | . | 33.2695 | 32.3116 | . | 29.7646 | . | . | 32.7813 | . |
| bta-miR-449c | . | . | . | . | . | . | 33.9152 | . | . | . | 32.8824 | 35.2932 |
| bta-miR-424-3p | . | . | . | . | . | . | . | . | . | . | . | . |
| bta-miR-449d | . | . | . | . | 35.0107 | 33.1009 | 33.8596 | 32.9081 | . | . | 32.8321 | 36.0919 |
| bta-miR-424-5p | 32.5336 | . | . | 34.1810 | 35.5854 | . | . | . | . | . | . | . |
| bta-miR-450a | . | . | . | . | . | . | . | . | . | . | . | . |
| bta-miR-425-3p | 32.4723 | . | . | . | 35.9329 | 30.7368 | 33.3083 | 32.7962 | . | . | 32.1639 | 32.7601 |
| bta-miR-450b | . | . | . | . | . | . | . | . | . | . | . | . |
| bta-miR-425-5p | 34.8359 | . | . | . | . | . | 35.0124 | . | . | . | . | . |
| bta-miR-451 | . | . | . | . | . | . | . | . | . | . | . | . |
| bta-miR-429 | 35.9611 | 32.2459 | 33.6305 | 32.6573 | 32.2034 | 30.5414 | 30.3136 | 33.1151 | 33.6305 | 36.3953 | 32.0754 | 31.0765 |
| bta-miR-452 | . | . | . | . | . | . | 34.6085 | . | . | . | . | . |
| bta-miR-431 | . | . | . | . | . | . | . | . | . | . | . | . |
| bta-miR-4523 | . | . | 35.8877 | 36.4017 | . | . | 34.8666 | 36.8509 | 35.8877 | . | 35.1428 | 35.5200 |
| bta-miR-432 | 34.9424 | . | . | 35.5499 | . | . | 31.3461 | . | . | . | 30.3136 | 32.9358 |
| bta-miR-453 | . | 36.4057 | 36.2101 | . | . | . | 34.6335 | . | . | . | 32.9538 | 34.8814 |
| bta-miR-433 | 31.8930 | 30.4069 | 28.8153 | 29.3664 | 32.8493 | 30.9091 | 30.2186 | 33.5136 | 28.8153 | 31.9433 | 30.5722 | 30.3427 |
| bta-miR-454 | . | . | . | . | . | . | 32.2671 | . | . | . | 31.9381 | 33.1557 |
| bta-miR-448 | . | . | . | . | . | . | 35.8722 | . | . | . | . | . |
| bta-miR-455-3p | . | . | . | . | . | . | 34.6563 | . | . | . | . | . |
| bta-miR-449a | . | . | . | . | . | . | 36.0651 | 33.0950 | . | . | . | . |
| bta-miR-455-5p | . | . | . | . | . | . | . | . | . | . | . | . |
| bta-miR-449b | . | . | . | . | . | . | 34.8829 | . | . | . | . | . |
| bta-miR-483 | . | . | . | . | . | 35.4207 | . | . | . | . | 35.4481 | . |
| bta-miR-484 | 34.0364 | . | . | . | . | . | . | 34.0322 | . | 35.8419 | . | 34.9139 |
| bta-miR-496 | . | . | . | . | . | . | 35.0714 | . | . | . | 34.4055 | 33.5788 |
| bta-miR-485 | . | . | . | 34.8735 | . | 36.8368 | 34.9842 | . | . | . | 34.0263 | . |
| bta-miR-497 | . | . | . | . | . | . | . | 33.2641 | . | . | . | . |
| bta-miR-486 | 33.6286 | . | . | . | 36.3341 | 32.7828 | 34.9421 | 32.2445 | . | . | 35.4372 | . |
| bta-miR-499 | . | . | . | . | . | . | . | . | . | . | . | . |
| bta-miR-487a | . | . | . | . | . | . | . | . | . | . | 34.8724 | . |
| bta-miR-500 | 33.7736 | . | . | . | . | . | . | . | . | . | . | . |
| bta-miR-487b | . | . | . | . | . | . | . | 35.6283 | . | . | . | . |
| bta-miR-502a | . | . | . | . | . | . | . | . | . | . | . | . |
| bta-miR-488 | . | . | . | . | . | . | 34.8244 | . | . | . | 34.5750 | 34.4788 |
| bta-miR-502b | . | . | . | . | . | 34.4550 | . | . | . | . | . | . |
| bta-miR-489 | . | 34.9317 | . | 35.2159 | 34.9825 | 34.2332 | 34.2023 | . | . | . | 34.4694 | 34.0032 |
| bta-miR-503-3p | 34.4055 | . | . | 34.8724 | 34.4553 | 32.4983 | 31.5761 | 30.8984 | . | . | 31.8525 | 32.7927 |
| bta-miR-490 | . | . | . | . | . | . | 33.0715 | 36.1290 | . | . | . | . |
| bta-miR-503-5p | . | 27.9393 | . | 18.2871 | . | . | . | . | . | . | 24.4655 | 34.8125 |
| bta-miR-491 | 34.0999 | . | . | . | . | . | 32.8301 | 34.9047 | . | . | 33.1930 | 35.7792 |
| bta-miR-504 | 35.7637 | . | . | . | . | . | . | 36.0424 | . | . | . | . |
| bta-miR-493 | . | . | . | . | 34.5875 | 34.0990 | 32.1189 | 33.0938 | . | . | 32.3659 | 34.9122 |
| bta-miR-505 | 32.5767 | . | . | . | . | . | 35.0468 | 32.8204 | . | . | 35.0382 | . |
| bta-miR-494 | 28.3453 | 30.8312 | 29.8328 | 30.4536 | 28.8058 | 25.8343 | 29.9510 | 27.3494 | 29.8328 | 30.8125 | 29.7290 | 30.7430 |
| bta-miR-532 | 33.1305 | . | . | 34.8479 | 32.9388 | . | 33.2107 | . | . | 34.0354 | 34.5842 | 33.8858 |
| bta-miR-495 | . | . | . | . | . | . | . | . | . | . | . | . |
| bta-miR-539 | . | . | . | . | . | . | . | . | . | . | . | . |
| bta-miR-541 | . | 33.8297 | 34.6828 | 33.8748 | 34.1074 | 32.3239 | 31.9207 | 34.2343 | 34.6828 | . | 32.3785 | 31.9840 |
| bta-miR-582 | . | . | . | . | . | . | . | . | . | . | . | . |
| bta-miR-542-5p | . | . | . | . | . | . | . | . | . | . | . | . |
| bta-miR-584 | . | . | . | . | . | 36.8985 | 34.8746 | . | . | . | . | . |
| bta-miR-543 | . | . | . | . | . | . | . | . | . | . | . | . |
| bta-miR-592 | . | . | . | . | . | . | 35.6735 | . | . | . | 35.6957 | . |
| bta-miR-544a | . | . | . | . | . | . | . | . | . | . | . | . |
| bta-miR-599 | . | . | . | . | . | . | . | . | . | . | . | . |
| bta-miR-544b | . | . | . | . | . | . | . | . | . | . | . | . |
| bta-miR-615 | 12.1437 | 11.4892 | 11.5965 | 11.3251 | 11.7342 | 11.7814 | 11.7244 | 11.3228 | 11.5965 | 11.8554 | 11.5882 | 11.3326 |
| bta-miR-545-3p | . | . | . | . | . | . | . | . | . | . | . | . |
| bta-miR-628 | . | . | 34.7308 | 34.8034 | . | . | 32.6669 | 34.0086 | 34.7308 | . | 32.8855 | 32.8347 |
| bta-miR-545-5p | . | . | . | . | . | . | . | . | . | . | 34.1350 | . |
| bta-miR-631 | 22.2985 | 19.7268 | 18.9791 | 18.9787 | 21.3397 | 19.5733 | 19.3643 | 22.4016 | 18.9791 | 23.9478 | 20.7170 | 19.9158 |
| bta-miR-551a | . | . | . | . | . | . | . | . | . | . | . | . |
| bta-miR-652 | 32.7966 | . | . | . | . | . | 35.1978 | . | . | . | . | . |
| bta-miR-551b | . | . | . | . | . | . | . | . | . | . | . | . |
| bta-miR-653 | . | . | . | . | . | . | . | . | . | . | . | . |
| bta-miR-562 | . | . | . | . | . | . | . | . | . | . | . | . |
| bta-miR-654 | . | . | . | . | . | 36.8402 | . | 32.9645 | . | . | . | . |
| bta-miR-568 | . | . | . | . | . | . | . | . | . | . | . | . |
| bta-miR-655 | . | . | . | . | . | . | 34.9591 | . | . | . | 34.7929 | . |
| bta-miR-574 | 31.0758 | 33.0696 | 33.1283 | 32.8303 | 28.5351 | 27.5073 | 31.4290 | 26.4982 | 33.1283 | 36.5842 | 28.8957 | 31.4702 |
| bta-miR-656 | . | 33.8776 | 32.6654 | 32.7364 | 34.2860 | 33.6520 | 32.6517 | 35.6752 | 32.6654 | . | 33.4261 | 32.4987 |
| bta-miR-658 | . | . | . | . | . | . | . | . | . | . | 36.9044 | . |
| bta-miR-758 | . | . | 35.3267 | . | . | . | . | . | 35.3267 | . | . | 35.0312 |
| bta-miR-660 | 35.9602 | . | . | . | . | . | 32.8747 | . | . | . | 33.8692 | 35.7858 |
| bta-miR-759 | . | . | . | . | . | . | . | . | . | . | . | . |
| bta-miR-664a | . | . | . | . | 34.2407 | 34.4148 | 33.8070 | 35.2604 | . | . | . | 34.2905 |
| bta-miR-760-3p | . | . | . | . | . | . | 33.9574 | 35.8898 | . | . | 35.3161 | 35.0605 |
| bta-miR-664b | 31.9499 | . | . | . | 34.3009 | 33.9447 | 34.9613 | 34.1679 | . | 36.8215 | 35.1926 | . |
| bta-miR-760-5p | 31.8379 | . | . | 34.1134 | 33.4687 | 32.9094 | 30.7198 | 31.3532 | . | . | 32.7478 | 32.5056 |
| bta-miR-665 | 34.2483 | . | . | . | 33.9370 | 31.4091 | . | 32.6993 | . | . | 33.9281 | 35.1131 |
| bta-miR-761 | . | . | . | . | . | . | . | 35.4243 | . | . | 36.9151 | . |
| bta-miR-669 | . | . | . | 35.5730 | 31.7902 | 31.7687 | 31.5082 | 30.8516 | . | . | 31.4566 | . |
| bta-miR-763 | . | . | . | 34.6551 | 35.8797 | 36.7804 | . | 33.9972 | . | . | 34.8724 | 34.9659 |
| bta-miR-670 | . | . | . | . | . | . | . | . | . | . | 36.1018 | . |
| bta-miR-764 | . | . | . | . | . | . | . | 35.6364 | . | . | . | 34.9419 |
| bta-miR-671 | . | . | . | . | . | . | . | 36.0761 | . | . | . | . |
| bta-miR-767 | 36.7716 | 33.9549 | 32.8315 | 33.0774 | 34.9681 | 32.0659 | 32.5830 | 33.9112 | 32.8315 | . | 34.9189 | 32.6276 |
| bta-miR-677 | 34.3609 | . | . | . | . | . | . | 36.9806 | . | . | 35.8614 | . |
| bta-miR-769 | . | . | . | . | . | . | 35.0842 | 35.9648 | . | . | 32.8437 | . |
| bta-miR-7 | 33.0160 | . | . | . | . | . | . | 33.3987 | . | . | . | . |
| bta-miR-873 | . | . | . | . | . | . | 34.0353 | . | . | . | . | . |
| bta-miR-708 | . | . | . | . | . | 35.9663 | 35.0038 | . | . | . | . | . |
| bta-miR-874 | . | . | . | . | . | . | 35.1619 | 33.4948 | . | . | . | . |
| bta-miR-744 | 33.9533 | 33.7916 | 35.8698 | . | 30.7157 | 30.6550 | 35.0131 | 30.2562 | 35.8698 | . | 33.9336 | . |
| bta-miR-875 | . | . | . | . | . | . | 33.9062 | . | . | . | 34.9452 | 34.9599 |
| bta-miR-876 | . | . | . | . | . | . | . | . | . | . | 35.5650 | . |
| bta-miR-98 | 31.4446 | . | . | . | 34.0537 | . | . | 33.0543 | . | . | . | . |
| bta-miR-877 | . | . | . | . | . | 35.8287 | 32.4225 | 33.9599 | . | . | 32.7840 | 33.7721 |
| bta-miR-99a-3p | . | . | . | . | . | . | 32.6334 | . | . | . | 33.7227 | . |
| bta-miR-885 | . | . | . | . | 36.5384 | . | 33.3393 | 36.9928 | . | . | 32.4945 | 33.0170 |
| bta-miR-99a-5p | 34.9053 | . | . | . | . | . | . | . | . | 35.3492 | . | . |
| bta-miR-9-3p | . | . | . | . | . | . | . | . | . | . | . | . |
| bta-miR-99b | 24.5998 | 23.3018 | 21.1714 | 22.1699 | 24.7431 | 22.8748 | 23.2209 | 25.5968 | 21.1714 | 24.5977 | 24.5195 | 23.7746 |
| bta-miR-9-5p | . | . | . | . | . | . | 34.3766 | . | . | . | 34.3210 | 33.9900 |
| bta-miR-1179 | . | . | . | 34.6916 | . | . | . | . | . | . | 34.7463 | . |
| bta-miR-92a | 29.3032 | . | . | 33.5412 | . | 32.6235 | 33.8048 | 29.5108 | . | . | 34.8936 | . |
| bta-miR-1185 | . | . | . | . | . | . | . | . | . | . | . | . |
| bta-miR-92b | 33.1418 | 32.9753 | 32.9044 | 32.8777 | 32.5948 | 30.6146 | 31.0786 | 31.2819 | 32.9044 | . | 29.8071 | 30.2334 |
| bta-miR-1193 | . | . | . | . | . | . | . | . | . | . | . | . |
| bta-miR-93 | 34.5710 | . | . | . | . | . | . | 32.1964 | . | . | . | 36.0023 |
| bta-miR-1197 | . | . | . | . | . | . | . | . | . | . | . | . |
| bta-miR-935 | . | . | . | . | 35.5011 | 33.4598 | 32.6111 | 34.1689 | . | . | 33.7286 | 34.6490 |
| bta-miR-122 | . | . | . | . | . | . | . | . | . | . | . | 34.8658 |
| bta-miR-940 | 32.4066 | . | . | 36.8733 | 34.8249 | 31.8996 | . | 31.9588 | . | . | 32.8201 | . |
| bta-miR-1224 | 34.2819 | . | . | 34.4538 | 33.4669 | 34.9133 | 33.5904 | 31.6506 | . | 35.4623 | 32.8038 | 35.5079 |
| bta-miR-95 | . | . | . | . | . | . | . | . | . | . | 35.0521 | . |
| bta-miR-1225-3p | 33.8604 | . | . | . | 33.8970 | 31.9569 | 35.5129 | 33.0053 | . | . | 33.2605 | 34.1263 |
| bta-miR-96 | . | . | . | . | . | . | 35.1298 | . | . | . | . | . |
| bta-miR-1246 | 27.3755 | 30.0785 | 28.7901 | 28.9034 | 26.5989 | 25.5448 | 30.5542 | 23.7961 | 28.7901 | 30.0979 | 27.6184 | 32.6107 |
| bta-miR-1247-3p | . | . | 33.6832 | 31.7063 | 33.9019 | 34.9955 | 31.6428 | 33.6760 | 33.6832 | . | 31.9829 | 33.0339 |
| bta-miR-1296 | . | . | . | . | . | 33.6681 | 32.8916 | . | . | . | 32.7716 | 34.4487 |
| bta-miR-1247-5p | 34.9951 | . | 34.8871 | . | 36.9928 | . | . | 34.7239 | 34.8871 | . | . | . |
| bta-miR-1298 | . | . | . | . | . | . | . | . | . | . | . | . |
| bta-miR-1248 | . | . | . | . | . | . | . | . | . | . | . | . |
| bta-miR-1301 | 36.4894 | . | . | . | . | . | . | . | . | . | . | . |
| bta-miR-1249 | . | . | . | . | . | . | . | . | . | . | . | . |
| bta-miR-1306 | 33.9356 | . | . | . | 34.4527 | 34.6863 | 32.1128 | 32.5890 | . | . | 32.7556 | 33.0154 |
| bta-miR-1260b | 27.1906 | 36.3851 | 32.0804 | 35.1181 | 30.2108 | 28.2464 | 32.8457 | 26.4281 | 32.0804 | 29.3194 | 30.1294 | 33.7232 |
| bta-miR-1307 | 34.3105 | 35.9487 | . | 34.9091 | 36.1567 | 32.9290 | 35.4056 | 33.0381 | . | 36.7601 | 33.1743 | 33.7840 |
| bta-miR-1271 | . | . | . | . | . | . | . | . | . | . | . | . |
| bta-miR-1343-3p | 35.3548 | . | . | . | . | 34.3209 | 32.8706 | 32.7790 | . | . | 33.4523 | . |
| bta-miR-1277 | . | . | . | . | . | . | . | . | . | . | . | . |
| bta-miR-1343-5p | 32.8263 | . | . | 35.5688 | 34.0025 | 33.1691 | . | 32.6796 | . | . | 36.1962 | 36.1294 |
| bta-miR-1281 | . | . | . | 32.9951 | . | 31.9410 | 32.3516 | 32.5159 | . | . | 31.0455 | 32.9258 |
| bta-miR-1388-3p | . | 36.7863 | . | 35.3805 | . | . | 36.5170 | 35.6263 | . | . | 35.7396 | 35.5214 |
| bta-miR-1282 | . | . | . | . | . | . | . | . | . | . | . | . |
| RNT43 snoRNA | 30.4981 | . | . | . | 34.5266 | 31.8553 | . | 32.5419 | . | 34.9616 | . | . |
| bta-miR-1284 | . | . | . | . | . | . | . | . | . | . | . | . |
| Hm/Ms/Rt T1 snRNA | 22.6585 | 29.0975 | 29.4449 | 27.4063 | 27.7294 | 27.0742 | 30.2098 | 25.5978 | 29.4449 | 27.7984 | 26.1580 | 30.6986 |
| bta-miR-1287 | . | . | . | . | . | . | . | . | . | . | . | . |
| bta-miR-99b | 24.5360 | 23.1711 | 21.3774 | 21.7380 | 24.6964 | 22.8635 | 23.0394 | 25.2990 | 21.3774 | 24.2534 | 24.3961 | 23.6566 |
| bta-miR-1291 | . | . | 36.8670 | . | . | . | . | . | 36.8670 | . | 35.0374 | . |
| Negative control | . | . | . | . | . | . | . | . | . | . | . | . |
| ^1^Body energy reserve: MBER: Cows with moderated body energy reserve; HBER: Cows with high body energy reserve. | | | | | | | | | | | | |
