# Supplementary material for: High body energy reserve influences extracellular vesicles miRNA contents within the ovarian follicle
Source: PLoS One. 2023 Jan 10;18(1):e0280195. doi: 10.1371/journal.pone.0280195 (PMC9831338; doi:10.1371/journal.pone.0280195)
Supplement: S4 Table — (DOCX) [file pone.0280195.s007.docx]

| **Supplementary table 4.** Raw cycle threshold levels of the 383 miRNAs profile in follicular fluid extracellular vesicles (FF EVs) from ipsi and contralateral ovarian follicles (3-6 mm in diameter) from cows with different body energy reserve. | | | | | | | | | | | | |
| --- | --- | --- | --- | --- | --- | --- | --- | --- | --- | --- | --- | --- |
| **MiRNAs** | **Body energy reserve^1^** | | | | | | | | | | | |
|  | **MBER** | | | | | | | | **HBER** | | | |
|  | **1** | **2** | **3** | **4** | **5** | **6** | **7** | **8** | **1** | **2** | **3** | **4** |
| bta-let-7a-3p | 32.4228 | 31.5078 | 31.6503 | 33.6150 | 30.5825 | . | 34.8630 | 29.8423 | 33.0103 | 32.6799 | 32.8767 | 33.9361 |
| bta-miR-103 | 31.1236 | 31.3223 | 30.2220 | 31.7174 | 28.7657 | 31.8996 | 32.9519 | 28.5728 | 30.8996 | 31.0321 | 30.8218 | 30.6779 |
| bta-let-7a-5p | 26.7365 | 26.2293 | 25.9156 | 27.2431 | 25.5704 | 27.4169 | 30.1855 | 25.7767 | 27.1868 | 27.0570 | 27.5731 | 26.6384 |
| bta-miR-105a | 30.9832 | . | 33.8898 | . | 32.8564 | 33.9507 | . | 32.9432 | . | 35.0364 | . | 32.5419 |
| bta-let-7b | 25.5766 | 24.7403 | 25.2329 | 25.7622 | 24.7464 | 25.8998 | 26.9993 | 24.4538 | 24.8582 | 25.7400 | 25.0831 | 25.3147 |
| bta-miR-105b | 31.0393 | 34.9981 | 34.7762 | 36.7867 | . | . | . | 32.4225 | 34.9487 | . | . | 31.5192 |
| bta-let-7c | 25.8030 | 25.1456 | 25.1794 | 26.2232 | 25.0407 | 26.1883 | 27.6499 | 24.6088 | 25.4922 | 26.1605 | 25.3909 | 25.5985 |
| bta-miR-106a | 25.7693 | 25.0272 | 24.3453 | 24.7342 | 24.1360 | 26.5594 | 29.5632 | 22.5894 | 24.3822 | 26.4648 | 25.8058 | 24.5030 |
| bta-let-7d | 28.5072 | 27.7561 | 27.5883 | 29.4502 | 26.9662 | 29.1954 | 31.6870 | 27.0728 | 29.1154 | 28.7851 | 28.7543 | 28.4652 |
| bta-miR-106b | 29.0310 | 28.7073 | 28.6133 | 28.7477 | 27.0708 | 30.6888 | 32.7442 | 25.7499 | 28.5190 | 29.6812 | 29.2870 | 28.5875 |
| bta-let-7e | 25.8844 | 25.7143 | 25.6689 | 26.8061 | 25.3125 | 26.8096 | 28.5943 | 25.7777 | 26.8376 | 26.6606 | 26.6757 | 26.8321 |
| bta-miR-107 | 33.5579 | 34.2404 | 33.8288 | . | 32.1112 | 34.7233 | . | 32.7584 | . | . | 35.0041 | 36.5599 |
| bta-let-7f | 28.7939 | 28.0970 | 27.7909 | 28.5613 | 27.4070 | 28.6922 | 32.5091 | 27.3307 | 28.7672 | 29.2516 | 29.2897 | 28.7572 |
| bta-miR-10a | 29.2027 | 28.7278 | 29.2311 | 29.9615 | 28.4994 | 30.6999 | 33.6378 | 28.5819 | 30.3422 | 30.8350 | 30.4833 | 29.8286 |
| bta-let-7g | 30.2968 | 28.5931 | 27.7863 | 28.9552 | 26.9986 | 31.1341 | 33.9057 | 26.6684 | 29.2052 | 30.3427 | 30.0564 | 28.2012 |
| bta-miR-10b | 29.6353 | 28.2817 | 28.8352 | 28.9502 | 27.8972 | 29.8023 | 31.8476 | 27.1947 | 29.6170 | 30.0968 | 29.4343 | 29.3593 |
| bta-let-7i | 30.4678 | 30.4667 | 29.7851 | 31.5483 | 28.7981 | 31.8890 | 32.4128 | 28.7357 | 30.8083 | 32.0591 | 31.7134 | 30.7494 |
| bta-miR-122 | 31.8864 | . | 35.4849 | . | 33.9465 | 36.4848 | . | 32.4473 | . | . | . | . |
| bta-miR-1 | 35.6191 | 33.6520 | . | . | . | . | . | 36.1135 | 35.2261 | . | . | . |
| bta-miR-124a | 33.6171 | . | 33.5591 | 34.9129 | 33.2353 | 36.8955 | . | 33.7149 | 34.7666 | . | . | 36.1281 |
| bta-miR-100 | 27.3328 | 26.5539 | 27.2604 | 28.1472 | 26.7753 | 29.1401 | 31.5578 | 26.7740 | 28.2314 | 28.6143 | 28.5954 | 28.1636 |
| bta-miR-124b | . | . | 36.3839 | 33.9487 | 33.6957 | 34.4239 | . | 32.8905 | 34.0009 | 35.4418 | 36.7406 | 33.8939 |
| bta-miR-101 | 34.1501 | 32.3122 | 30.9559 | 30.5158 | 29.9062 | . | . | 27.6389 | 30.7502 | 34.5389 | . | 29.5032 |
| bta-miR-125a | 29.7724 | 30.3313 | 29.7410 | 29.7311 | 27.7803 | 30.7677 | 33.6682 | 28.1014 | 29.9611 | 29.7493 | 30.4188 | 29.7426 |
| bta-miR-125b | 28.2830 | 26.6026 | 26.8217 | 26.5974 | 26.4886 | 27.7183 | 30.7197 | 25.7519 | 25.9847 | 27.5790 | 27.7634 | 26.7529 |
| bta-miR-133b | 33.9688 | . | 35.6960 | 34.8837 | 33.9251 | . | . | . | . | . | . | . |
| bta-miR-126-3p | 27.6414 | 29.0583 | 28.9387 | 29.7983 | 29.5036 | 32.2961 | 32.9377 | 27.7751 | 28.7046 | 30.9613 | 30.8280 | 30.0796 |
| bta-miR-133c | 29.7590 | . | 34.6218 | . | 33.9014 | 34.8435 | . | . | . | . | . | 36.0791 |
| bta-miR-126-5p | 26.2022 | 26.1628 | 26.4822 | 27.5442 | 26.6237 | 27.8351 | 28.0916 | 24.9549 | 25.7785 | 27.6425 | 26.6421 | 27.2444 |
| bta-miR-134 | 27.3659 | 34.1607 | 33.0286 | 32.4321 | 30.2382 | 33.0082 | 33.1450 | 31.1164 | 31.7912 | 34.3739 | . | 30.8096 |
| bta-miR-127 | 30.7168 | 31.8859 | 31.1933 | 32.0147 | 30.5582 | 32.4954 | 32.3764 | 30.4066 | 32.9544 | 32.9742 | 31.8468 | 30.7121 |
| bta-miR-135a | 34.9460 | 34.2594 | 35.4655 | 33.5262 | 34.1691 | 35.7546 | . | 32.8487 | 33.9448 | . | 35.3489 | 33.9575 |
| bta-miR-128 | 28.5798 | 27.8082 | 27.8735 | 29.1496 | 27.5039 | 29.7003 | 32.3674 | 26.3765 | 28.7456 | 29.3745 | 29.7318 | 28.8268 |
| bta-miR-135b | 36.0047 | 35.2784 | . | . | 34.2151 | . | . | 34.2456 | . | 36.8814 | . | 33.9485 |
| bta-miR-129 | 32.0819 | 31.9367 | 33.0180 | 31.5310 | 29.4454 | 32.9453 | 36.1299 | 29.9026 | 31.9060 | 34.3028 | 35.0105 | 29.8934 |
| bta-miR-136 | . | 35.4264 | 34.8898 | . | 34.5331 | 34.5826 | . | 33.0342 | 35.0635 | . | 35.0949 | 33.6781 |
| bta-miR-129-3p | 32.9595 | . | . | 35.0417 | 34.1764 | . | . | 31.7545 | 35.0402 | . | . | . |
| bta-miR-137 | . | . | 33.9069 | . | . | . | . | . | 33.6531 | . | . | . |
| bta-miR-129-5p | 31.0122 | 32.5931 | 31.8928 | 32.5889 | 29.4655 | 31.8908 | 36.3964 | 30.2408 | 32.7090 | 33.0767 | 34.9641 | 30.6239 |
| bta-miR-138 | 26.7509 | 31.8403 | 32.2412 | 34.0342 | 30.7808 | 33.3739 | 33.4450 | 31.8431 | 34.1830 | 33.3263 | 32.9037 | 31.1384 |
| bta-miR-130a | 29.6399 | 28.8479 | 28.5248 | 29.8705 | 28.0053 | 30.7608 | 29.9978 | 26.0006 | 29.2946 | 30.1403 | 28.7213 | 29.0393 |
| bta-miR-139 | 31.7227 | 33.9617 | 33.1589 | 34.6059 | 30.8350 | . | 33.9518 | 31.5338 | 33.0909 | 33.8280 | 31.7979 | 32.8215 |
| bta-miR-130b | 29.9667 | 29.8433 | 28.7499 | 30.6126 | 28.1278 | 30.8411 | 31.7753 | 26.7214 | 29.7086 | 30.4710 | 28.8467 | 29.3173 |
| bta-miR-140 | 27.9940 | 27.7804 | 26.6921 | 28.8081 | 26.8220 | 28.8196 | 29.7728 | 25.3496 | 27.7360 | 28.4985 | 26.7216 | 28.7083 |
| bta-miR-132 | 26.6552 | 27.6625 | 28.3148 | 27.8400 | 27.7775 | 28.2127 | 33.2859 | 26.8941 | 27.6982 | 29.7819 | 28.7401 | 27.7155 |
| bta-miR-141 | 31.4331 | . | . | . | . | . | . | 34.0654 | . | . | 33.6902 | . |
| bta-miR-133a | 28.4781 | 34.0005 | 31.6101 | 33.8756 | 30.5731 | 31.8095 | 35.8533 | 29.7482 | 31.4540 | . | 33.5147 | 30.1999 |
| bta-miR-142-3p | 31.4671 | . | 33.4683 | . | 32.8181 | . | . | . | . | . | . | . |
| bta-miR-142-5p | 32.5181 | . | . | . | . | . | . | 36.4960 | . | . | . | . |
| bta-miR-151-3p | 28.3935 | 28.3983 | 27.7243 | 28.8305 | 27.7991 | 29.0531 | 30.5382 | 26.7391 | 28.0278 | 29.1314 | 28.7670 | 28.1975 |
| bta-miR-143 | 29.5047 | 30.7542 | 30.5590 | 30.7553 | 29.8380 | 32.2576 | 32.5436 | 29.0104 | 29.7486 | 31.4621 | 33.5028 | 29.9782 |
| bta-miR-151-5p | 30.6188 | 30.6818 | 29.7941 | 30.7249 | 29.1692 | 32.1540 | 32.8775 | 29.7096 | 31.4900 | 31.8498 | 31.7056 | 30.7119 |
| bta-miR-144 | 32.8571 | . | . | . | . | . | . | . | . | . | . | . |
| bta-miR-152 | 28.6821 | 31.3618 | 30.9745 | 31.3191 | 30.4051 | 32.6573 | . | 29.2707 | 30.6831 | 32.0940 | 32.1316 | 30.9927 |
| bta-miR-145 | 27.8867 | 27.0294 | 27.3086 | 27.7874 | 26.7036 | 29.6206 | 29.7461 | 25.7341 | 26.9732 | 29.8425 | 28.3775 | 28.1673 |
| bta-miR-153 | . | . | 35.2468 | . | . | 35.5119 | . | 36.8271 | 35.8931 | 35.4707 | . | 35.0823 |
| bta-miR-146a | 33.4573 | . | 34.7184 | . | 34.6387 | . | . | 33.3193 | 33.5267 | 33.7860 | 35.0080 | 34.4454 |
| bta-miR-154a | 29.5635 | . | . | 35.7357 | . | 36.5649 | . | . | . | . | . | . |
| bta-miR-146b | 31.9353 | 35.4319 | . | . | 34.1279 | . | . | 32.2972 | . | 34.5278 | 35.0799 | 36.5694 |
| bta-miR-154b | 27.2709 | 32.8409 | 32.9530 | 32.3862 | 33.9009 | 32.5109 | 32.0863 | 31.8717 | 33.9719 | 32.7117 | 31.7890 | 32.0118 |
| bta-miR-147 | . | . | 34.7574 | . | 33.2167 | 34.4247 | . | 32.9357 | . | . | . | . |
| bta-miR-154c | 31.7529 | 33.0618 | 33.8930 | 34.3448 | 32.4900 | . | . | 31.5802 | 32.6236 | 33.7405 | 35.1018 | . |
| bta-miR-148a | 28.4351 | 26.8332 | 26.6782 | 26.6476 | 25.8886 | 28.7001 | 33.1440 | 24.7356 | 26.3462 | 28.7710 | 28.7396 | 26.7724 |
| bta-miR-155 | 29.8867 | 30.3809 | 30.7092 | 30.7577 | 29.4119 | 31.4013 | 32.2543 | 29.4949 | 29.5001 | 30.3795 | 29.8976 | 30.8211 |
| bta-miR-148b | 28.8819 | 27.6253 | 27.7217 | 27.3010 | 26.4773 | 29.4587 | . | 25.4233 | 26.7125 | 29.6019 | 29.8403 | 27.1694 |
| bta-miR-15a | 26.4079 | 25.8111 | 25.5529 | 25.7798 | 25.3761 | 27.2023 | 33.7772 | 24.0270 | 25.3229 | 27.6821 | 27.0948 | 25.4005 |
| bta-miR-149-3p | 23.2398 | 23.4274 | 22.3210 | 24.8034 | 23.3729 | 24.5964 | 26.2070 | 22.4597 | 23.7692 | 24.7095 | 24.2058 | 24.4370 |
| bta-miR-15b | 26.7626 | 26.4339 | 25.8663 | 26.5414 | 25.2949 | 27.4094 | 29.6963 | 24.4551 | 26.1153 | 27.7109 | 26.7673 | 26.3455 |
| bta-miR-149-5p | 29.0026 | 31.6956 | 30.8070 | 33.0522 | 29.4158 | 31.7857 | 36.2637 | 29.8734 | 32.2642 | 31.7248 | 31.7554 | 29.6981 |
| bta-miR-16a | 25.9539 | 25.4842 | 24.8049 | 25.5106 | 24.6162 | 26.6498 | 29.7464 | 23.0574 | 24.7665 | 26.4205 | 26.3375 | 24.9083 |
| bta-miR-150 | 30.6527 | 36.0265 | 33.9239 | 36.7149 | 31.5226 | 36.0438 | 35.0545 | 32.4333 | 32.8444 | . | . | 35.3759 |
| bta-miR-16b | 24.8358 | 24.3704 | 23.7556 | 24.6230 | 23.6180 | 25.6198 | 28.6124 | 22.2334 | 23.8277 | 25.5424 | 25.0268 | 24.1022 |
| bta-miR-17-3p | 33.4909 | 35.5283 | 34.1641 | 36.8912 | 33.0874 | 34.2547 | . | 33.1913 | 34.2541 | . | 33.7296 | 32.8376 |
| bta-miR-188 | 29.0703 | 28.7557 | 28.0667 | 28.4587 | 27.3090 | 29.3216 | 31.1733 | 26.7711 | 27.6993 | 28.4654 | 28.8869 | 27.7711 |
| bta-miR-17-5p | 29.7769 | 29.7086 | 29.0846 | 30.0129 | 28.2818 | 30.7176 | 33.2897 | 27.6108 | 29.7940 | 30.2762 | 30.6470 | 29.5706 |
| bta-miR-18a | 30.7425 | 30.4603 | 29.7213 | 30.1411 | 28.8734 | 31.7012 | . | 27.7111 | 29.3417 | 32.3479 | 31.7542 | 29.3807 |
| bta-miR-181a | 30.3294 | 30.6564 | 29.8195 | 31.1566 | 29.5578 | 31.7838 | 32.9816 | 28.6074 | 31.2526 | 31.8053 | 30.4650 | 31.0727 |
| bta-miR-18b | 31.7586 | 33.4158 | 32.7350 | 31.3404 | 30.7982 | 34.1250 | . | 28.8932 | 30.5116 | 33.5739 | 33.9386 | 30.6741 |
| bta-miR-181b | 29.8830 | 29.8369 | 29.7141 | 29.7779 | 28.7563 | 30.7109 | 33.0760 | 28.7018 | 30.0467 | 30.7994 | 30.3145 | 30.6072 |
| bta-miR-190a | 32.5633 | . | 36.3765 | . | 33.8579 | 36.5483 | . | 34.3610 | . | . | . | 35.4685 |
| bta-miR-181c | 32.0773 | 32.3693 | 30.2518 | 32.3770 | 29.7870 | 35.5699 | 33.8783 | 29.0776 | 30.7631 | 31.7118 | 30.9309 | 32.4060 |
| bta-miR-190b | 32.4437 | 31.6403 | 31.2800 | 32.7257 | 31.8356 | 32.8262 | 34.8259 | 29.8184 | 32.2584 | 33.1410 | 30.4314 | 31.2958 |
| bta-miR-181d | 28.3788 | 29.1625 | 29.6810 | 29.5540 | 28.7181 | 29.7769 | 31.5329 | 28.3909 | 28.7702 | 29.7495 | 30.8300 | 28.8231 |
| bta-miR-191 | 27.9814 | 27.9108 | 28.6909 | 28.8009 | 27.7723 | 29.7825 | 35.2976 | 26.4018 | 28.8762 | 30.4536 | 30.5047 | 28.6149 |
| bta-miR-182 | 31.6896 | . | . | . | 35.5619 | . | . | 35.4662 | . | . | . | 34.9392 |
| bta-miR-192 | 30.2134 | 31.5569 | 31.7657 | 32.0465 | 30.3048 | 32.8743 | 36.9990 | 29.7534 | 31.8910 | 33.4457 | 31.8952 | 30.7823 |
| bta-miR-183 | 30.5403 | 36.9023 | . | . | . | . | . | 35.9293 | . | . | 34.1759 | 35.9822 |
| bta-miR-193a | 35.3375 | . | . | . | . | . | . | . | . | . | . | . |
| bta-miR-184 | 29.7009 | . | . | . | . | . | . | . | 36.6117 | . | . | . |
| bta-miR-193a-3p | 34.1963 | 33.3968 | 31.7807 | 34.3233 | 30.6144 | 36.5464 | 34.0276 | 29.5353 | 33.9172 | 34.5439 | 32.7295 | 32.9013 |
| bta-miR-185 | 30.6256 | 30.6772 | 29.9009 | 30.7008 | 28.6758 | 31.7811 | 31.8797 | 28.3391 | 30.7442 | 31.2711 | 30.2229 | 29.6738 |
| bta-miR-193a-5p | 27.9134 | 28.5166 | 27.7558 | 28.5190 | 27.3261 | 28.2646 | 28.6544 | 26.3627 | 27.8258 | 29.0177 | 27.1410 | 27.8123 |
| bta-miR-186 | 28.6601 | 29.7076 | 28.7322 | 30.0979 | 28.1175 | 30.9023 | 32.6668 | 27.1450 | 28.8653 | 31.5097 | 29.7405 | 29.6772 |
| bta-miR-193b | 29.6202 | 29.7203 | 29.5541 | 30.6039 | 28.4844 | 30.5189 | 32.5528 | 27.4991 | 29.6967 | 31.5201 | 28.9425 | 29.8528 |
| bta-miR-187 | 28.4819 | 30.0615 | 29.3750 | 30.6054 | 28.5156 | 30.1520 | 31.9584 | 28.4913 | 30.2612 | 30.0756 | 30.0847 | 28.7177 |
| bta-miR-194 | 32.8445 | 32.7579 | 32.7544 | 32.6847 | 31.4743 | 34.8110 | . | 31.0786 | 31.9774 | 34.4434 | . | 32.1351 |
| bta-miR-195 | 26.7789 | 26.1563 | 25.7267 | 26.4423 | 25.7151 | 27.3380 | 30.6947 | 24.7001 | 25.8388 | 27.4483 | 27.0438 | 25.8064 |
| bta-miR-200c | 31.1271 | 36.6591 | 35.9860 | . | 36.3394 | . | . | 33.3894 | 36.5223 | . | 29.7339 | 35.3729 |
| bta-miR-196a | 28.7697 | 33.9088 | 32.8617 | 33.5386 | 33.8794 | 32.8099 | . | 32.0911 | 34.0478 | 34.1375 | 34.7717 | 32.8648 |
| bta-miR-202 | 27.0171 | 26.3465 | 25.6341 | 25.0903 | 24.1578 | 28.0998 | 32.2620 | 22.7310 | 25.5829 | 27.0876 | 27.8403 | 24.6404 |
| bta-miR-196b | 31.4707 | 33.8507 | 34.3470 | . | 34.2962 | 36.7366 | 34.9747 | 32.9237 | 35.7974 | 33.9578 | . | 32.0148 |
| bta-miR-204 | 28.8547 | 31.1705 | 30.5738 | 31.9238 | 31.5105 | 32.4874 | 33.7327 | 29.7261 | 30.7437 | 33.0044 | 30.4975 | 31.9681 |
| bta-miR-197 | 27.7222 | 28.8959 | 28.1172 | 28.7778 | 26.7775 | 28.6520 | 32.0911 | 26.6228 | 28.7198 | 29.7391 | 29.3715 | 27.7523 |
| bta-miR-205 | 29.4967 | . | 33.8841 | 34.1190 | 30.7973 | 33.5449 | 34.5310 | 32.0085 | 34.5524 | 34.1476 | 31.7630 | 32.9476 |
| bta-miR-199a-3p | 27.2414 | 26.2073 | 26.6269 | 26.8165 | 25.7540 | 28.3316 | 29.6883 | 25.4449 | 26.1272 | 28.0205 | 28.2462 | 26.6414 |
| bta-miR-206 | 27.8370 | 31.7322 | 31.1553 | 30.8235 | 29.8153 | 31.7731 | 33.7511 | 30.5487 | 31.3961 | 31.7619 | 32.9053 | 30.2727 |
| bta-miR-199a-5p | 32.8806 | 32.8519 | 34.1920 | 33.0442 | 31.8171 | . | 34.3415 | 30.6768 | 32.9197 | . | 33.0117 | 33.9446 |
| bta-miR-208a | . | 36.3132 | . | . | . | . | . | 33.9864 | . | . | . | . |
| bta-miR-199b | 33.8675 | 31.8674 | 33.2756 | 32.1033 | 33.9310 | 34.9370 | . | 31.7640 | 33.2870 | 34.5386 | 34.0760 | 32.9445 |
| bta-miR-208b | . | . | . | . | . | . | 36.4235 | . | . | . | . | . |
| bta-miR-199c | 25.7261 | 24.7683 | 25.4254 | 25.6791 | 25.2630 | 26.8384 | 28.7195 | 24.3730 | 24.8073 | 26.8186 | 26.7616 | 25.6094 |
| bta-miR-20a | 25.3155 | 24.8366 | 23.9511 | 24.7968 | 23.7958 | 26.1800 | 27.7894 | 22.1781 | 24.1566 | 25.8426 | 25.2403 | 24.4275 |
| bta-miR-19a | 35.6973 | 32.8558 | 31.3003 | 30.8129 | 30.6387 | . | 34.7350 | 26.4072 | 31.2800 | 34.2570 | 31.6965 | 29.7468 |
| bta-miR-20b | 26.6881 | 26.1262 | 25.4020 | 25.8479 | 24.9039 | 27.6566 | 29.7357 | 23.5864 | 25.3960 | 27.2815 | 26.7625 | 25.7726 |
| bta-miR-19b | 31.4194 | 33.8840 | 31.6854 | 32.0065 | 29.7367 | 31.4026 | 32.3749 | 26.3931 | 32.2478 | 32.9410 | 30.9312 | 30.1197 |
| bta-miR-21-3p | 33.9581 | 34.5320 | 31.8231 | 34.1406 | 31.2464 | 35.5155 | . | 31.6793 | 32.9643 | 36.8073 | 32.8696 | 32.1199 |
| bta-miR-200a | . | . | 34.2440 | . | 34.4964 | . | . | . | . | . | . | 34.4191 |
| bta-miR-21-5p | 31.2950 | 30.8349 | 31.8431 | 31.1447 | 30.6039 | 32.6507 | . | 31.2218 | 32.0963 | 31.7735 | 32.8760 | 31.7250 |
| bta-miR-200b | 30.3477 | 31.5795 | 30.1840 | 32.4841 | 30.6253 | 32.9201 | . | 29.8784 | 30.7501 | 32.8104 | 27.7001 | 31.8771 |
| bta-miR-210 | 29.2249 | 29.4182 | 28.2168 | 29.3312 | 27.4176 | 30.5031 | 30.6963 | 25.7377 | 29.1403 | 29.7547 | 27.7555 | 28.7443 |
| bta-miR-211 | 29.7140 | 31.0659 | 30.6635 | 31.5328 | 30.8150 | 33.2661 | 32.7663 | 29.8321 | 32.3835 | 32.0088 | 30.5695 | 31.0697 |
| bta-miR-22-5p | 31.9620 | 31.8519 | 31.0039 | 32.6676 | 29.7973 | 34.8031 | . | 29.7672 | 31.2563 | 33.8498 | 31.8522 | 32.1809 |
| bta-miR-212 | . | . | . | . | . | . | 35.2012 | . | . | . | . | 35.1307 |
| bta-miR-221 | 27.7132 | 27.3908 | 27.3457 | 28.4344 | 26.9021 | 29.1730 | 28.5326 | 25.8100 | 27.5146 | 28.6763 | 27.4431 | 27.7396 |
| bta-miR-214 | 28.6101 | 28.8466 | 28.3817 | 29.9440 | 27.7837 | 28.9640 | 31.1452 | 27.3499 | 28.8501 | 31.6009 | 30.1580 | 27.7749 |
| bta-miR-222 | 28.8342 | 29.3457 | 29.5867 | 30.9557 | 28.8327 | 30.9105 | 30.6162 | 28.1913 | 29.5034 | 30.2645 | 28.9518 | 30.4596 |
| bta-miR-215 | 30.7801 | 30.4764 | 31.1897 | 30.3109 | 29.7196 | 31.9536 | 33.6409 | 29.4630 | 30.8296 | 31.9983 | 31.6630 | 30.6811 |
| bta-miR-223 | 31.5458 | 33.5204 | 34.5751 | . | 33.4819 | . | . | 32.9085 | 35.4411 | 34.4659 | . | 33.7687 |
| bta-miR-216a | . | . | 35.8870 | . | 32.2047 | . | . | 33.7839 | . | 35.3007 | . | 33.7852 |
| bta-miR-224 | 31.5254 | 30.3466 | 29.3253 | 30.8745 | 28.7588 | 30.8667 | 32.9469 | 28.0947 | 30.0591 | 30.5149 | 30.0627 | 29.6967 |
| bta-miR-216b | . | . | . | . | 36.5151 | 36.5269 | . | . | . | . | . | 35.0157 |
| bta-miR-23a | 24.3164 | 23.8651 | 24.4422 | 24.8625 | 23.6997 | 25.2934 | 26.5342 | 23.4106 | 24.4004 | 25.3886 | 24.3918 | 24.8442 |
| bta-miR-217 | . | . | . | . | . | . | . | . | . | . | 36.5946 | . |
| bta-miR-23b-3p | 26.7601 | 27.1201 | 26.9342 | 28.0803 | 26.5413 | 27.6612 | 29.7217 | 26.6953 | 28.4917 | 27.5156 | 26.9290 | 27.7170 |
| bta-miR-218 | 32.1876 | 33.5794 | 35.2766 | 35.6930 | 35.0120 | 35.6500 | . | 33.5224 | 33.9825 | . | . | 34.2206 |
| bta-miR-23b-5p | 36.3099 | 35.5891 | 32.7832 | . | 33.7719 | . | . | 32.2090 | 35.0095 | 35.7148 | 32.2199 | 33.8645 |
| bta-miR-219 | 28.7147 | 31.9068 | 31.8650 | 32.2806 | 30.5082 | 32.3573 | 33.9981 | 30.3931 | 32.2475 | 34.3453 | 32.7036 | 30.8281 |
| bta-miR-24 | 35.1847 | . | . | . | . | . | . | . | . | . | . | . |
| bta-miR-219-3p | 30.7222 | . | 34.0062 | 35.5791 | 31.1091 | 36.1194 | . | 31.5257 | 33.2554 | 33.7197 | . | 31.6248 |
| bta-miR-24-3p | 26.6153 | 26.1072 | 25.7584 | 25.6605 | 24.8250 | 27.4483 | 30.7487 | 23.7223 | 25.2864 | 27.7219 | 26.7303 | 25.6395 |
| bta-miR-219-5p | . | . | . | . | 35.7124 | . | . | . | . | . | . | . |
| bta-miR-25 | 22.8001 | 22.5804 | 22.4982 | 22.7170 | 22.0300 | 22.7425 | 24.8119 | 20.9735 | 22.6336 | 23.5023 | 22.0723 | 22.5775 |
| bta-miR-22-3p | . | 3.2601 | 3.0317 | 2.9693 | . | 2.8317 | 2.8798 | 2.9011 | . | 3.0328 | 2.8339 | 3.0108 |
| bta-miR-26a | 26.6917 | 26.7723 | 26.5436 | 28.2898 | 25.8019 | 28.3471 | 31.8221 | 26.1486 | 27.7834 | 28.0645 | 27.7951 | 27.3574 |
| bta-miR-26b | 30.2085 | 29.2331 | 29.2139 | 30.3954 | 28.3666 | 32.3850 | . | 28.7362 | 30.3577 | 30.3925 | 31.4242 | 29.7309 |
| bta-miR-29d-3p | 29.8317 | 28.8796 | 28.7988 | 29.0397 | 28.0188 | 30.2522 | 32.9151 | 27.6188 | 28.8198 | 30.6049 | 30.2155 | 28.5791 |
| bta-miR-26c | . | . | . | . | . | . | . | 42.2589 | . | . | . | . |
| bta-miR-29d-5p | 31.8798 | 34.8662 | 33.3113 | . | 33.0252 | . | 34.4433 | 31.7889 | 33.6344 | 34.2056 | 34.2972 | 34.2051 |
| bta-miR-27a-3p | 27.2697 | 27.5374 | 27.1953 | 27.5753 | 26.2067 | 28.6288 | 29.4542 | 25.0254 | 27.0793 | 28.7165 | 26.9626 | 26.7138 |
| bta-miR-29e | . | . | . | . | 43.8504 | . | . | . | . | . | 34.5373 | . |
| bta-miR-27a-5p | . | 36.9155 | 33.9796 | 8.2319 | 32.1650 | 34.4102 | 8.7284 | 33.0417 | . | 28.5175 | 10.8155 | 31.8468 |
| bta-miR-301a | 34.4754 | . | 35.3799 | 36.9715 | 34.9203 | . | . | 34.1295 | . | . | . | . |
| bta-miR-27b | 25.9579 | 25.8033 | 25.0413 | 24.9141 | 24.7432 | 26.8823 | 26.9167 | 23.0813 | 25.2637 | 26.9333 | 24.7876 | 25.0699 |
| bta-miR-301b | 34.6598 | . | . | . | 36.1314 | . | . | . | . | . | . | . |
| bta-miR-28 | 33.9500 | 31.7954 | 31.8460 | 34.8322 | 31.4189 | 33.9546 | . | 30.4372 | 31.7813 | 33.6675 | 32.3278 | 31.5523 |
| bta-miR-302a | . | . | . | . | . | . | . | . | . | . | . | . |
| bta-miR-296-3p | 25.7393 | 25.7359 | 25.5346 | 26.3561 | 25.0815 | 31.8751 | 27.4460 | 24.2527 | 25.8070 | 33.1374 | 25.2313 | 26.4410 |
| bta-miR-302b | 32.2820 | . | . | . | . | . | . | . | . | . | . | . |
| bta-miR-296-5p | 29.0219 | 31.3941 | 30.7528 | 31.7964 | 28.8870 | 31.5767 | . | 28.4729 | 30.7918 | 31.8586 | 31.9069 | 29.7891 |
| bta-miR-302c | 30.0601 | . | 35.1261 | . | 33.8408 | . | . | . | . | . | . | 32.3891 |
| bta-miR-299 | 32.1301 | . | . | . | 34.6484 | . | . | . | . | . | . | . |
| bta-miR-302d | . | . | . | . | . | . | . | 34.7441 | . | . | . | . |
| bta-miR-29a | 26.1354 | 25.4836 | 25.0413 | 24.7200 | 24.3337 | 26.6447 | 28.5971 | 23.5024 | 24.5578 | 26.6013 | 25.4807 | 24.7656 |
| bta-miR-3064 | 33.9681 | . | . | . | . | . | 35.7205 | . | . | . | . | 36.7723 |
| bta-miR-29b | 35.4296 | . | 34.4473 | 36.8010 | 32.5539 | . | . | 32.8340 | 32.8882 | . | . | 32.8026 |
| bta-miR-30a-5p | 29.4231 | 28.9331 | 28.1781 | 28.9829 | 26.9188 | 30.7738 | 32.9101 | 25.7407 | 28.4252 | 30.3583 | 29.8262 | 28.3113 |
| bta-miR-29c | 26.2108 | 25.7291 | 24.8577 | 25.2604 | 24.4422 | 26.6891 | 29.4056 | 23.4650 | 24.7321 | 26.7808 | 25.6061 | 24.7004 |
| bta-miR-30b-3p | 33.0079 | . | 33.0252 | . | 33.6104 | 35.0577 | . | 32.7210 | 34.4991 | . | . | 34.7993 |
| bta-miR-30b-5p | 30.3065 | 31.4023 | 30.1202 | 31.5974 | 29.7465 | 31.7920 | 33.8327 | 28.4130 | 30.9047 | 32.6182 | 30.6738 | 31.2337 |
| bta-miR-328 | 28.9470 | 29.7274 | 28.7381 | 29.6897 | 27.5656 | 28.6989 | 31.0280 | 26.8397 | 29.0480 | 31.6243 | 29.7539 | 28.4644 |
| bta-miR-30c | 29.1946 | 29.9755 | 28.7254 | 30.4782 | 27.8039 | 30.3994 | 32.1287 | 27.4531 | 29.8042 | 30.6989 | 29.1179 | 29.8279 |
| bta-miR-329a | . | . | 34.5003 | . | 34.2355 | . | . | 33.8390 | . | . | . | . |
| bta-miR-30d | 28.9748 | 29.6764 | 28.1669 | 28.6981 | 27.1658 | 30.3798 | 31.8552 | 25.7758 | 28.7922 | 29.8655 | 29.0565 | 28.8080 |
| bta-miR-329b | . | . | . | . | . | . | . | . | . | . | . | 35.5156 |
| bta-miR-30e-5p | 29.3592 | 29.4992 | 28.2698 | 28.8159 | 27.1074 | 31.0971 | 31.9210 | 25.7813 | 28.7026 | 29.7130 | 30.1556 | 28.6201 |
| bta-miR-330 | 31.6201 | 32.2045 | 32.3054 | 32.3072 | 29.8367 | 30.9183 | 34.2130 | 29.8558 | 32.7595 | 32.9744 | 32.8025 | 29.7451 |
| bta-miR-30f | 29.8048 | 31.0667 | 29.8962 | 31.3922 | 29.1047 | 31.8221 | 33.7510 | 28.4039 | 31.1925 | 31.4135 | 29.9433 | 31.3408 |
| bta-miR-331-3p | 32.6118 | 32.5433 | 32.9321 | 32.5749 | 31.5949 | 33.3883 | 33.9611 | 30.7397 | 32.7105 | 33.9687 | 32.3635 | 35.6981 |
| bta-miR-31 | 28.7125 | 28.7568 | 27.8844 | 29.8044 | 27.7666 | 29.8542 | 31.5704 | 26.7256 | 28.9401 | 29.1951 | 28.4624 | 28.6410 |
| bta-miR-331-5p | 28.3079 | 30.4564 | 28.1238 | 29.9447 | 28.0001 | 30.8055 | 30.2736 | 27.7745 | 29.4745 | 29.7648 | 28.4392 | 28.4614 |
| bta-miR-32 | . | . | . | . | . | . | 36.0465 | . | . | . | . | . |
| bta-miR-335 | 31.2325 | 29.4401 | 29.7927 | 29.3810 | 28.4239 | 31.8373 | 34.4914 | 26.7714 | 29.9351 | 31.8268 | 31.6509 | 29.1258 |
| bta-miR-320a | 21.5181 | 21.7355 | 21.8394 | 22.6954 | 21.7273 | 22.7118 | 22.7781 | 20.6537 | 21.9973 | 22.8727 | 21.7383 | 22.1649 |
| bta-miR-338 | . | . | 33.2737 | 34.5871 | 32.5619 | 35.5626 | . | 33.3011 | . | 34.4044 | . | 34.6877 |
| bta-miR-320b | 28.7020 | 29.0654 | 28.8042 | 28.9884 | 27.8178 | 29.5763 | 31.2624 | 27.6630 | 28.2209 | 29.6634 | 30.4288 | 27.7636 |
| bta-miR-339a | 28.0457 | 27.9802 | 27.7445 | 29.8208 | 26.8319 | 29.7223 | 30.5125 | 25.8608 | 28.5863 | 30.1849 | 27.7811 | 28.0003 |
| bta-miR-323 | 19.5343 | 19.3921 | 19.4683 | 19.1106 | 20.4556 | 19.6812 | 18.8213 | 20.5126 | 19.4994 | 18.2217 | 19.0694 | 19.2659 |
| bta-miR-339b | 26.5555 | 26.3640 | 26.6526 | 28.8146 | 25.5567 | 27.7508 | 28.6052 | 24.6548 | 27.4233 | 28.3626 | 26.1593 | 27.2862 |
| bta-miR-324 | 28.7976 | 29.9875 | 30.1834 | 30.8388 | 29.5363 | 33.5745 | 32.2625 | 28.4584 | 29.6541 | 32.7399 | 31.2386 | 30.0773 |
| bta-miR-33a | . | . | . | . | 33.8263 | . | . | . | . | . | . | . |
| bta-miR-326 | 29.2146 | 29.7414 | 28.8136 | 30.5705 | 27.1702 | 29.8182 | 32.3738 | 27.3206 | 28.8338 | 29.8103 | 29.8372 | 27.3586 |
| bta-miR-33b | . | 36.8159 | . | . | 36.0927 | 34.9609 | . | 36.0024 | 36.0410 | . | . | 34.2862 |
| bta-miR-340 | 29.8077 | . | . | 34.9427 | 36.6068 | . | . | . | . | . | . | . |
| bta-miR-365-3p | 32.6354 | 30.0546 | 28.5648 | 28.8642 | 27.1969 | 31.8406 | 31.8183 | 26.4317 | 28.7925 | 31.7168 | 30.6928 | 28.2832 |
| bta-miR-342 | 29.9419 | 31.1182 | 29.9635 | 31.4340 | 29.4739 | 31.2461 | 34.0180 | 28.2393 | 30.8258 | 31.6872 | 30.6134 | 30.5925 |
| bta-miR-365-5p | 27.6044 | 25.7018 | 25.8145 | 25.7934 | 23.8229 | 27.3012 | 30.7555 | 24.2640 | 25.3958 | 26.7117 | 27.8238 | 24.8173 |
| bta-miR-345-3p | 29.6828 | 29.6500 | 29.2864 | 29.3639 | 28.4782 | 29.9928 | 30.7074 | 27.9464 | 29.2833 | 29.7541 | 29.6871 | 28.7793 |
| bta-miR-367 | 34.7159 | . | . | . | . | . | . | . | . | . | . | . |
| bta-miR-345-5p | 30.7357 | 33.9442 | 33.2093 | 33.9768 | 30.7399 | 33.1936 | 35.2530 | 30.9650 | 32.8942 | 36.0795 | 35.6768 | 30.7074 |
| bta-miR-369-3p | 33.0428 | 33.0362 | 33.0873 | 32.9728 | 32.9462 | . | . | 31.0570 | 33.0953 | . | . | 33.8720 |
| bta-miR-346 | 28.6873 | 30.2691 | 28.6778 | 29.7257 | 26.9583 | 29.0786 | 31.8964 | 27.5268 | 29.5271 | 30.7147 | 31.2310 | 26.8104 |
| bta-miR-369-5p | 30.7812 | . | 33.4772 | . | 33.6968 | . | . | 33.9530 | 34.5242 | . | . | . |
| bta-miR-34a | 34.0930 | 32.7157 | 31.7492 | 34.4680 | 31.7485 | 34.6774 | . | 31.7766 | 34.0622 | 33.9960 | 33.1614 | 33.2453 |
| bta-miR-370 | 32.4314 | 31.7342 | 31.1842 | 31.8268 | 29.1586 | 32.4322 | 32.2833 | 29.4555 | 31.2921 | 33.7397 | 32.0136 | 29.7943 |
| bta-miR-34b | 33.1425 | . | . | 33.6535 | . | . | . | 36.3868 | . | . | . | . |
| bta-miR-371 | 32.3838 | . | . | . | 36.1405 | . | . | . | 35.6168 | . | 36.9974 | . |
| bta-miR-34c | 33.3970 | . | . | . | 36.7622 | . | . | . | 36.6227 | 34.3593 | . | . |
| bta-miR-374a | 31.7048 | 31.3115 | 30.9747 | 32.8527 | 30.0471 | 32.3217 | 34.3959 | 30.6604 | 31.8787 | 32.6026 | 32.4497 | 31.0056 |
| bta-miR-361 | 28.0171 | 27.6947 | 26.1398 | 27.2379 | 26.7693 | 28.4460 | 28.7774 | 25.8052 | 27.8046 | 28.6504 | 27.9647 | 27.6707 |
| bta-miR-374b | 29.2573 | 29.2956 | 28.5940 | 30.3678 | 27.7700 | 30.0304 | 31.6783 | 27.9883 | 29.1233 | 29.4588 | 28.9218 | 29.1948 |
| bta-miR-362-3p | 28.2825 | 27.8755 | 27.8192 | 28.4072 | 26.8866 | 29.6953 | 32.1331 | 26.2337 | 28.2613 | 29.8339 | 28.1699 | 28.8167 |
| bta-miR-375 | 29.2239 | 28.7202 | 30.1555 | 31.3601 | 30.0698 | 31.3811 | 32.5707 | 29.6095 | . | 31.8794 | 30.7537 | 29.7311 |
| bta-miR-362-5p | 32.9264 | 33.7169 | 33.1522 | 35.0307 | 31.5381 | 34.2267 | . | 31.8588 | 33.8683 | 33.3021 | 34.0050 | 32.1679 |
| bta-miR-376a | 30.2585 | . | 35.0718 | 36.0036 | 36.9752 | . | . | 33.0261 | . | . | . | 34.0182 |
| bta-miR-363 | 31.8270 | . | 34.9008 | . | 33.9403 | 36.5986 | . | . | . | . | . | 36.4402 |
| bta-miR-376b | 34.3792 | . | . | . | . | . | . | . | . | . | . | . |
| bta-miR-376c | . | . | . | . | . | . | . | . | . | . | . | . |
| bta-miR-382 | 28.7280 | 29.8014 | 30.2978 | 30.7094 | 29.8281 | 30.8158 | 30.8382 | 28.8351 | 29.7354 | 31.8185 | 30.4791 | 30.8824 |
| bta-miR-376d | 31.6176 | . | 34.9145 | . | . | . | . | 32.9241 | . | . | . | . |
| bta-miR-383 | 29.5382 | 36.1138 | 33.9419 | 33.9873 | 31.8847 | 35.1707 | . | 32.9063 | 36.9540 | 36.8475 | 32.1430 | 32.8115 |
| bta-miR-376e | . | . | . | . | 34.5473 | . | . | . | . | . | . | . |
| bta-miR-409a | 34.2874 | . | . | . | . | . | . | 35.7653 | 35.6773 | 36.0067 | 35.6608 | 34.7958 |
| bta-miR-377 | . | . | . | 34.1261 | . | . | . | 35.8850 | . | . | . | . |
| bta-miR-409b | 32.5758 | . | . | . | . | . | . | . | . | . | . | . |
| bta-miR-378 | 28.4652 | 30.3029 | 29.7932 | 30.3264 | 29.1232 | 30.7383 | 32.6733 | 27.8393 | 30.5140 | 31.0851 | 30.0792 | 29.4253 |
| bta-miR-410 | 32.9122 | 34.2049 | 32.6365 | 35.4684 | 32.1773 | 35.0566 | . | 30.8135 | 35.2453 | . | 35.2373 | 31.7845 |
| bta-miR-378b | 29.5039 | 32.3079 | 31.3031 | 31.8249 | 29.5525 | 30.6477 | 32.7431 | 27.8056 | 31.7597 | 30.7648 | 29.7729 | 30.3925 |
| bta-miR-411a | 28.7740 | 30.7080 | 32.4110 | 31.9038 | 31.0563 | 32.1236 | 32.7028 | 30.5301 | 32.1288 | 32.7645 | 31.8660 | 32.8024 |
| bta-miR-378c | 33.9019 | . | 34.1716 | 36.7926 | 32.8686 | 33.3378 | 35.4795 | 32.6445 | 34.5980 | 36.9652 | 33.9941 | 33.6384 |
| bta-miR-411b | 31.6881 | 34.2318 | 33.4977 | 35.1399 | . | 35.3808 | 34.3713 | 36.9217 | 36.0926 | 32.5750 | 33.9117 | 33.9601 |
| bta-miR-378d | 29.9578 | 31.6493 | 33.0832 | 32.8155 | 30.6076 | 34.4088 | 33.3600 | 30.5251 | 33.8781 | 33.2931 | 32.1410 | 31.0976 |
| bta-miR-411c-3p | 34.0439 | 36.3331 | . | 36.3452 | . | . | . | . | . | . | . | . |
| bta-miR-379 | 32.0382 | 33.8559 | 32.7478 | 34.5051 | 33.5556 | . | 34.2687 | . | . | . | 34.6418 | . |
| bta-miR-411c-5p | . | . | . | . | . | . | . | . | . | . | . | . |
| bta-miR-380-3p | 30.8003 | 33.1738 | 30.7250 | 32.8114 | 31.8743 | 32.7301 | 31.7244 | 30.6980 | 31.8630 | 34.0011 | 31.5230 | 31.8975 |
| bta-miR-412 | 34.9657 | . | 36.4852 | . | 32.8161 | 34.2180 | . | 34.8347 | . | . | . | 33.7187 |
| bta-miR-380-5p | 28.3175 | 33.9779 | 32.9624 | 35.0617 | 31.3854 | 33.9474 | 34.1998 | 31.8654 | 32.9067 | . | 35.4233 | 31.5525 |
| bta-miR-421 | 27.6532 | 28.3462 | 27.8000 | 29.0799 | 27.7740 | 29.7286 | 29.6419 | 26.8290 | 28.8917 | 28.9164 | 27.9513 | 29.3318 |
| bta-miR-381 | 35.3731 | 34.9417 | 32.6047 | 34.0969 | 31.6039 | 34.2196 | . | 31.4384 | 33.9008 | . | . | 31.5532 |
| bta-miR-423-3p | 26.6177 | 26.3132 | 26.1567 | 26.8547 | 25.7165 | 27.8365 | 28.9787 | 24.4354 | 26.1952 | 27.7713 | 26.5401 | 26.5471 |
| bta-miR-423-5p | 23.9366 | 24.1172 | 24.1912 | 23.9972 | 24.0000 | 24.3298 | 25.9930 | 23.4330 | 23.6811 | 24.7609 | 23.5652 | 23.4978 |
| bta-miR-449c | 30.3339 | 35.4185 | 32.8567 | 35.0980 | 30.7156 | 35.9677 | . | 33.8737 | 33.8886 | 34.8873 | 34.8010 | 32.7191 |
| bta-miR-424-3p | 29.6021 | 28.2630 | 28.4275 | 30.6104 | 28.3335 | 30.4195 | 29.7549 | 27.4927 | 29.2678 | 30.8684 | 28.3546 | 29.3579 |
| bta-miR-449d | 29.5819 | 30.6483 | 30.1490 | 30.4929 | 28.0090 | 30.7917 | 32.9290 | 28.5245 | 30.5292 | 30.6872 | 32.3404 | 28.5593 |
| bta-miR-424-5p | 23.8257 | 22.2529 | 23.1342 | 23.6225 | 22.7487 | 25.7376 | 27.9292 | 21.7414 | 22.7535 | 25.2688 | 23.7377 | 23.2842 |
| bta-miR-450a | 35.9438 | 34.9584 | 35.5235 | 34.9235 | 33.8663 | . | . | 33.0735 | 33.9464 | 36.4079 | . | 33.8733 |
| bta-miR-425-3p | 26.7430 | 27.7257 | 25.4479 | 28.0999 | 25.7709 | 28.4087 | 27.5735 | 24.6860 | 27.3056 | 27.6856 | 26.2941 | 26.6696 |
| bta-miR-450b | 32.6831 | 31.8977 | 32.6082 | 31.6919 | 31.5532 | 35.9722 | . | 30.7637 | 32.5563 | 34.4145 | 34.7596 | 31.8790 |
| bta-miR-425-5p | 27.7652 | 27.6731 | 27.5562 | 28.7946 | 27.2437 | 29.3416 | 30.0672 | 26.3754 | 28.0130 | 28.8163 | 28.4532 | 28.6448 |
| bta-miR-451 | 32.8714 | 32.2473 | 33.6965 | 34.4225 | 32.9252 | 35.0159 | 33.6355 | 31.0317 | 32.0354 | 34.6378 | 34.8470 | 33.3370 |
| bta-miR-429 | 29.2020 | 31.5155 | 31.5899 | 30.7711 | 31.8787 | 31.5872 | 30.7956 | 30.3191 | 31.2056 | 31.1995 | 30.6342 | 31.2643 |
| bta-miR-452 | 33.5362 | 36.3244 | 35.0514 | 34.1677 | 32.1145 | . | 34.3094 | 32.7943 | 36.6242 | 33.5559 | 33.5617 | 34.8693 |
| bta-miR-431 | 32.0012 | 36.1599 | 31.8005 | 33.2758 | 30.0162 | 33.0461 | . | 30.6408 | 36.4190 | 34.6460 | 33.9526 | 30.7494 |
| bta-miR-4523 | 31.7464 | 33.0613 | 33.4886 | 32.2944 | 30.6594 | 33.3023 | . | 31.9736 | 32.8989 | 34.6302 | 35.9951 | 31.1312 |
| bta-miR-432 | 27.8987 | 30.4767 | 30.2935 | 30.6013 | 30.1565 | 31.5454 | 30.6923 | 29.0073 | 30.3885 | 31.7811 | 30.7520 | 30.0097 |
| bta-miR-453 | 32.8577 | 34.3048 | 34.8210 | 34.8144 | 33.7641 | 33.8836 | 36.0543 | 32.9350 | 33.9052 | . | 35.3546 | 35.2606 |
| bta-miR-433 | 27.8141 | 31.8505 | 31.8632 | 31.8638 | 29.7696 | 31.6758 | 32.7923 | 30.5532 | 31.5184 | 31.4971 | 32.7715 | 29.7750 |
| bta-miR-454 | 28.8730 | 34.2575 | 34.2672 | 35.2446 | 33.3025 | . | . | . | . | . | . | . |
| bta-miR-448 | 35.0924 | . | . | . | 33.8388 | . | . | . | . | . | . | 34.6332 |
| bta-miR-455-3p | 30.8625 | 35.3241 | . | . | 34.1433 | 35.5828 | 34.5190 | 34.7922 | . | . | 33.8564 | 33.4337 |
| bta-miR-449a | . | 33.9624 | 35.9633 | 33.0707 | 36.7609 | . | 35.0208 | . | 35.8506 | . | 36.6686 | 33.8736 |
| bta-miR-455-5p | . | . | 33.8559 | 33.8862 | 33.4348 | . | . | 30.7286 | 32.6031 | . | 34.8730 | 33.2049 |
| bta-miR-449b | . | 34.3794 | 36.9146 | 36.7370 | 33.8557 | . | . | 33.9213 | 35.0553 | . | 34.9370 | 33.5080 |
| bta-miR-483 | 34.3259 | 33.2595 | 31.5598 | 33.9823 | 31.8383 | 32.4629 | 32.8751 | 29.8241 | 30.6547 | 34.9786 | 32.8214 | 32.0629 |
| bta-miR-484 | 27.3398 | 26.8588 | 26.7817 | 28.3089 | 25.8353 | 28.7547 | 29.5658 | 24.8226 | 27.2038 | 28.2347 | 26.3709 | 27.3765 |
| bta-miR-496 | 31.7996 | . | 36.0630 | . | 32.7863 | . | 36.5241 | 33.2384 | . | . | . | 34.9115 |
| bta-miR-485 | 31.8630 | . | 34.4322 | 35.6130 | 34.2251 | . | . | 34.7563 | . | . | 36.7495 | . |
| bta-miR-497 | 30.8398 | 30.7341 | 29.5963 | 30.4112 | 29.1796 | 29.6981 | 33.6317 | 27.4979 | 30.0404 | 32.0781 | 30.7469 | . |
| bta-miR-486 | 28.2447 | 29.2051 | 27.7774 | 29.9264 | 27.1209 | 28.9311 | 30.1478 | 26.7612 | 28.8278 | 30.4912 | 28.5998 | 27.6808 |
| bta-miR-499 | . | 35.4355 | 34.9341 | . | 34.0187 | . | . | 35.3786 | 33.5062 | . | . | . |
| bta-miR-487a | . | 34.1991 | 35.7615 | . | 33.3672 | . | . | 33.9102 | 34.0744 | . | 36.2680 | . |
| bta-miR-500 | 30.8312 | 30.6882 | 30.0460 | 30.7209 | 29.6303 | 31.7411 | 32.6591 | 28.7066 | 30.5812 | 31.7844 | 30.0304 | 30.4043 |
| bta-miR-487b | 34.7440 | 32.1305 | 33.1148 | 33.9744 | 31.6759 | . | 33.6950 | 31.4314 | 31.8680 | . | 32.8707 | 34.6517 |
| bta-miR-502a | 32.9128 | 32.1267 | . | 32.9039 | 32.8861 | 34.2271 | . | 33.1397 | 34.8957 | 34.1161 | 35.0320 | 32.8619 |
| bta-miR-488 | 32.0157 | . | . | . | 32.2486 | 34.9390 | . | 33.8142 | 35.5143 | 36.4275 | 35.9183 | 33.5905 |
| bta-miR-502b | 30.1885 | 29.8287 | 29.9972 | 29.8445 | 28.8689 | 30.7611 | 32.6378 | 28.3430 | 29.4457 | 30.7860 | 30.1384 | 29.8980 |
| bta-miR-489 | 32.9267 | 35.2008 | 35.4119 | . | 35.5486 | 35.7445 | 35.3027 | 34.5509 | 36.0319 | 35.8466 | 36.0299 | . |
| bta-miR-503-3p | 27.3799 | 28.8159 | 28.5507 | 29.5536 | 27.2864 | 29.2545 | 31.7379 | 27.3678 | 28.7866 | 29.9304 | 28.5408 | 27.5207 |
| bta-miR-490 | 31.4561 | 35.3214 | 32.2731 | 32.9987 | 30.1983 | 32.2865 | 36.2800 | 31.6517 | 32.9725 | 35.0128 | 34.1823 | 30.7755 |
| bta-miR-503-5p | 31.8050 | 33.8718 | 34.0736 | 34.5414 | 31.9347 | 35.3721 | . | 31.4318 | 33.0897 | 35.2774 | 32.9361 | 31.8386 |
| bta-miR-491 | 29.7265 | 32.6368 | 31.9603 | 31.8328 | 30.6389 | 32.1702 | 35.4830 | 30.5065 | 31.7624 | 32.8157 | 32.7558 | 31.6258 |
| bta-miR-504 | 32.4787 | 35.0071 | 33.9204 | 32.9877 | 30.2517 | 33.6714 | 32.0805 | 28.3732 | 34.8355 | 33.5791 | 30.5598 | 30.5409 |
| bta-miR-493 | 28.7378 | 30.8911 | 29.7207 | 30.7611 | 27.6349 | 29.9247 | 32.7557 | 28.4094 | 29.8051 | 31.7532 | 30.8141 | 27.6975 |
| bta-miR-505 | 27.3751 | 27.3217 | 26.7756 | 28.3306 | 26.2777 | 28.7198 | 28.8544 | 25.6005 | 27.7585 | 28.5461 | 26.6983 | 28.2468 |
| bta-miR-494 | 23.6960 | 23.8975 | 22.7070 | 25.8406 | 22.6614 | 25.1333 | 25.5433 | 22.4946 | 24.5845 | 24.9326 | 23.5160 | 23.5459 |
| bta-miR-532 | 30.1559 | 31.6601 | 30.8111 | 31.3445 | 30.6368 | 32.7750 | 33.8865 | 28.3526 | 30.4087 | 32.3626 | 30.9877 | 30.4407 |
| bta-miR-495 | . | . | 35.0511 | . | 33.8317 | . | . | 31.8101 | 34.7269 | . | . | 34.9652 |
| bta-miR-539 | 33.6338 | . | 33.8845 | 36.7008 | 34.3507 | . | . | 36.8718 | 34.8747 | . | . | 34.8084 |
| bta-miR-541 | 29.7635 | 32.7068 | 31.8001 | 31.8791 | 29.5406 | 31.1867 | 32.9011 | 29.9863 | 31.1629 | 32.6907 | 32.7441 | 29.6118 |
| bta-miR-582 | 34.9909 | . | . | . | . | . | . | . | . | . | . | . |
| bta-miR-542-5p | 31.9530 | 31.9086 | 31.8224 | 32.9912 | 32.0648 | . | 34.3456 | 30.5139 | 31.7984 | 35.1698 | 33.1743 | 34.7986 |
| bta-miR-584 | 30.6239 | 31.3907 | 30.6969 | 31.8535 | 29.4699 | 31.1608 | 32.0326 | 29.7959 | 30.7539 | 31.0729 | 32.4298 | 29.0787 |
| bta-miR-543 | . | . | 36.0913 | . | . | . | . | 35.1840 | . | . | . | 34.6476 |
| bta-miR-592 | 29.6881 | 30.1736 | 29.3733 | 32.0904 | 29.8713 | 31.9295 | 35.0220 | 30.0754 | 30.7753 | 30.0886 | 30.3185 | 31.2216 |
| bta-miR-544a | . | . | . | 34.2023 | . | . | . | . | . | . | . | . |
| bta-miR-599 | . | . | . | . | . | . | . | . | . | . | . | . |
| bta-miR-544b | 33.9224 | . | . | 33.8548 | 32.7872 | . | . | 36.6403 | . | . | . | 33.8519 |
| bta-miR-615 | 12.9510 | 12.8858 | 12.7690 | 12.7656 | 12.6331 | 12.7566 | 12.7762 | 12.7248 | 12.7568 | 12.4785 | 13.2102 | 12.7763 |
| bta-miR-545-3p | . | . | . | . | . | . | . | . | . | . | . | . |
| bta-miR-628 | 28.9576 | 34.1735 | 35.7985 | . | 33.2055 | . | 36.5633 | 33.9043 | . | . | 33.2464 | . |
| bta-miR-545-5p | 32.5423 | 33.6958 | . | 35.8115 | 33.1459 | . | 34.5204 | 33.3470 | 33.9047 | 33.9298 | 36.3976 | 32.3153 |
| bta-miR-631 | 20.5944 | 20.4416 | 20.5926 | 20.4080 | 21.6651 | 20.4415 | 19.9901 | 21.1955 | 20.7295 | 19.8389 | 20.2980 | 20.5669 |
| bta-miR-551a | 33.8517 | . | . | . | 35.0002 | . | . | . | . | . | . | . |
| bta-miR-652 | 29.8711 | 29.0903 | 28.7726 | 30.1914 | 28.4663 | 30.6940 | 30.6852 | 28.1687 | 29.3769 | 30.4416 | 29.1216 | 29.9764 |
| bta-miR-551b | 34.1637 | 35.1802 | . | 35.0919 | 33.8979 | 36.7175 | . | 33.2635 | . | 36.5289 | . | . |
| bta-miR-653 | . | . | . | 36.6975 | . | . | . | 35.1248 | . | . | . | . |
| bta-miR-562 | . | . | . | . | . | . | . | . | . | . | . | . |
| bta-miR-654 | 34.1927 | 33.6177 | . | . | 33.3018 | 35.7362 | . | 32.5239 | 36.5564 | . | 35.3761 | 32.8881 |
| bta-miR-568 | . | . | . | . | . | . | . | . | . | . | . | . |
| bta-miR-655 | 32.9023 | 33.8892 | 35.8603 | . | 33.4607 | . | . | . | 36.5255 | . | 36.5642 | . |
| bta-miR-574 | 23.0212 | 23.4137 | 22.5879 | 22.8356 | 20.6028 | 22.6650 | 26.4392 | 21.6005 | 22.8427 | 24.1427 | 24.7051 | 20.8014 |
| bta-miR-656 | 31.3137 | 36.7164 | 34.2362 | 35.7623 | 35.7201 | 35.4679 | 35.0241 | 35.7159 | 33.9454 | 36.4669 | 35.2212 | 34.2007 |
| bta-miR-658 | 31.9142 | 32.3885 | 31.8807 | 33.6027 | 30.9159 | 32.9277 | 35.1295 | 31.0598 | 31.9031 | 34.1078 | 33.2968 | 31.6936 |
| bta-miR-758 | 32.2758 | . | 34.9371 | . | 32.8780 | 36.3403 | . | . | 34.8054 | . | 34.2496 | 35.9208 |
| bta-miR-660 | 26.4971 | 26.1933 | 26.1069 | 26.6604 | 25.7090 | 27.2110 | 28.6780 | 24.7377 | 26.5924 | 27.4752 | 25.8205 | 26.5533 |
| bta-miR-759 | . | . | . | . | . | . | . | . | . | . | . | . |
| bta-miR-664a | 29.1982 | 28.6178 | 28.6299 | 27.8345 | 26.5134 | 28.6693 | 32.5890 | 26.7243 | 27.7377 | 28.7275 | 30.2303 | 26.8276 |
| bta-miR-760-3p | 27.8034 | 27.7365 | 27.7033 | 27.9579 | 26.6917 | 27.8225 | 28.9686 | 26.3803 | 27.1125 | 28.5277 | 27.7446 | 26.7749 |
| bta-miR-664b | 31.0692 | 30.1744 | 31.2154 | 32.5852 | 29.8440 | 32.2935 | 33.7166 | 30.0590 | 32.5440 | 31.7844 | 30.7969 | 32.2321 |
| bta-miR-760-5p | 27.8214 | 29.7734 | 29.1172 | 29.7243 | 27.4712 | 29.3893 | 31.6589 | 27.9219 | 29.4528 | 30.7655 | 29.9452 | 27.7499 |
| bta-miR-665 | 28.4351 | 28.7950 | 28.0909 | 29.3021 | 26.7651 | 28.2354 | 31.4656 | 27.0304 | 28.4409 | 29.7445 | 29.9112 | 26.9461 |
| bta-miR-761 | . | . | . | . | 33.8576 | . | 36.6585 | 34.0377 | 33.3927 | . | . | 32.8395 |
| bta-miR-669 | 25.6993 | 26.5893 | 25.3764 | 25.8252 | 22.9505 | 25.4692 | 29.8188 | 23.8126 | 25.5616 | 27.4428 | 27.8260 | 23.4048 |
| bta-miR-763 | 31.8238 | 32.9253 | 33.2374 | 32.6265 | 29.5895 | 32.7950 | . | 31.0324 | 31.9064 | 33.2852 | 34.1051 | 30.0200 |
| bta-miR-670 | 34.7366 | 35.0533 | . | 36.6449 | . | . | 34.9367 | . | 35.4883 | 34.5060 | 35.3414 | 33.9287 |
| bta-miR-764 | 33.4116 | 36.3804 | 35.1791 | . | 31.7014 | 34.9224 | . | 33.2291 | 34.3646 | . | . | 33.5640 |
| bta-miR-671 | 31.7435 | 30.6125 | 30.6779 | 31.6921 | 30.4000 | 31.8712 | 31.7824 | 29.8858 | 30.6421 | 31.5082 | 30.6326 | 30.8224 |
| bta-miR-767 | 32.6307 | 33.9124 | 33.7265 | 33.8807 | 31.9892 | 34.1959 | 33.1502 | 32.7857 | 32.9136 | 34.2967 | 32.2372 | 32.5189 |
| bta-miR-677 | 29.3988 | 29.0085 | 28.1391 | 30.6322 | 28.5973 | 31.3111 | 30.0690 | 27.0185 | 31.0643 | 30.4203 | 29.3256 | 31.5912 |
| bta-miR-769 | 31.8754 | . | 34.9155 | 34.6264 | 30.7490 | 34.8158 | . | 31.7737 | 33.5359 | 34.3489 | 34.2070 | 32.0499 |
| bta-miR-7 | . | 33.8481 | . | . | . | . | . | 33.9009 | . | 35.0304 | 34.8719 | 34.5725 |
| bta-miR-873 | 32.9992 | 35.2196 | 35.4196 | . | 32.6307 | . | . | 33.1071 | 33.9157 | 35.0714 | . | 33.1870 |
| bta-miR-708 | 29.9695 | 31.6810 | 31.8206 | 32.6971 | 31.0047 | 33.2095 | . | 30.5298 | 32.8158 | 33.7898 | 31.9847 | 31.8600 |
| bta-miR-874 | 29.8215 | 30.0811 | 29.4911 | 30.7779 | 27.8817 | 30.3550 | 31.7845 | 28.8217 | 29.7908 | 30.2308 | 30.6778 | 28.4665 |
| bta-miR-744 | 27.3194 | 27.7802 | 27.5578 | 28.9683 | 27.2606 | 28.0824 | 28.6462 | 26.5882 | 27.7785 | 28.8219 | 26.9229 | 28.8926 |
| bta-miR-875 | 32.9081 | . | . | . | 35.1776 | . | . | . | . | . | . | . |
| bta-miR-876 | 36.2115 | 34.3705 | . | 35.8240 | 34.3202 | 36.7007 | . | 35.0202 | 34.5882 | 35.9453 | 35.0592 | . |
| bta-miR-98 | 34.1061 | 33.8880 | 33.5127 | 35.4199 | 32.8964 | 34.2395 | . | 33.7086 | 33.3808 | . | 34.8782 | 33.3749 |
| bta-miR-877 | 26.7577 | 26.7596 | 26.7023 | 26.8400 | 25.7602 | 27.4689 | 28.0098 | 25.5300 | 26.4106 | 27.8308 | 26.8512 | 25.8346 |
| bta-miR-99a-3p | 29.7983 | 29.3708 | 29.6041 | 31.4909 | 29.4581 | 31.5539 | 30.4614 | 27.8282 | 30.1281 | 31.7781 | 28.7707 | 32.0868 |
| bta-miR-885 | 31.5688 | 32.8741 | 31.9083 | 32.0423 | 30.3725 | 32.1149 | . | 30.0141 | 32.8894 | 33.9263 | 32.0303 | 29.7857 |
| bta-miR-99a-5p | 26.3265 | 24.8274 | 25.5089 | 25.7044 | 24.7839 | 27.6479 | 29.7567 | 23.7772 | 25.3757 | 26.7446 | 26.6990 | 26.7477 |
| bta-miR-9-3p | . | . | . | . | . | . | . | . | . | . | . | . |
| bta-miR-99b | 24.0727 | 23.7476 | 23.8140 | 24.1548 | 24.9161 | 24.3077 | 23.6423 | 24.7382 | 24.2490 | 23.0970 | 24.0140 | 23.7879 |
| bta-miR-9-5p | 31.7910 | . | . | . | 35.1638 | . | . | 34.1134 | 34.5150 | . | . | . |
| bta-miR-1179 | 31.5101 | . | . | . | 33.1639 | . | . | 36.1207 | . | . | . | . |
| bta-miR-92a | 22.4779 | 22.2003 | 21.9972 | 22.6397 | 21.7054 | 22.8181 | 24.6015 | 20.6643 | 22.3863 | 23.2544 | 22.2748 | 21.8099 |
| bta-miR-1185 | . | . | . | . | . | . | . | . | . | . | . | . |
| bta-miR-92b | 26.8117 | 26.7733 | 26.1626 | 27.2480 | 25.3860 | 27.4747 | 28.6328 | 24.8106 | 26.8070 | 27.7818 | 27.2755 | 25.6847 |
| bta-miR-1193 | . | . | . | . | . | . | . | . | . | . | . | . |
| bta-miR-93 | 24.7906 | 24.4538 | 24.0437 | 25.1375 | 23.9418 | 25.7110 | 26.1923 | 22.5471 | 24.6178 | 25.5644 | 24.5020 | 25.1515 |
| bta-miR-1197 | 33.9846 | . | 36.9055 | . | . | . | . | . | . | . | . | . |
| bta-miR-935 | 29.7469 | 31.4212 | 31.8857 | 32.7816 | 30.1814 | 31.5656 | 32.8177 | 30.6792 | 31.9088 | 32.8704 | 32.6853 | 30.8057 |
| bta-miR-122 | 34.0032 | . | 35.6655 | . | 34.4213 | 33.7846 | . | 33.3930 | 34.8764 | . | 34.5701 | 33.9590 |
| bta-miR-940 | 25.3142 | 23.7681 | 24.0438 | 24.8187 | 23.8194 | 25.7321 | 26.7162 | 23.3901 | 24.7441 | 25.7801 | 25.5569 | 24.1012 |
| bta-miR-1224 | 19.2112 | 18.6878 | 18.5709 | 20.2122 | 19.2068 | 20.4460 | 20.7146 | 17.9197 | 19.4977 | 20.5220 | 19.6513 | 20.2018 |
| bta-miR-95 | 33.7050 | . | . | 33.6170 | 32.8550 | 34.3331 | . | 32.9045 | 34.4899 | 33.9751 | . | . |
| bta-miR-1225-3p | 26.6665 | 27.5638 | 26.2550 | 27.3752 | 24.8271 | 26.8512 | 30.1466 | 25.3571 | 26.8466 | 27.6961 | 27.9107 | 25.2518 |
| bta-miR-96 | . | . | . | . | . | . | . | . | . | . | . | . |
| bta-miR-1246 | 15.1996 | 14.7367 | 13.8231 | 14.1552 | 14.7244 | 14.1588 | 16.7983 | 13.8262 | 14.0462 | 14.2366 | 15.3026 | 13.7766 |
| bta-miR-1247-3p | 28.1163 | 30.4295 | 28.7303 | 30.4517 | 27.8190 | 29.1566 | 31.6961 | 28.0462 | 29.7253 | 30.8576 | 29.7232 | 28.6361 |
| bta-miR-1296 | 28.9338 | 30.8375 | 29.5410 | 30.6602 | 27.2050 | 29.9183 | 32.9625 | 28.0486 | 30.4084 | 32.1671 | 30.6618 | 27.9840 |
| bta-miR-1247-5p | 27.4084 | 27.0554 | 25.7175 | 27.6566 | 25.3499 | 28.3901 | 28.7333 | 24.6925 | 27.1991 | 28.6008 | 26.9219 | 26.6553 |
| bta-miR-1298 | . | . | 35.9578 | . | 34.9747 | . | . | . | 35.1795 | 35.5576 | . | 34.6465 |
| bta-miR-1248 | 31.8950 | 32.6860 | 31.8034 | 32.2809 | 31.9080 | 33.9598 | 32.8221 | 31.7750 | 33.1249 | 32.4276 | 32.8955 | 32.9029 |
| bta-miR-1301 | 35.8258 | 35.0005 | . | . | 34.6031 | . | 34.0729 | 33.8360 | 32.8843 | . | 35.9527 | . |
| bta-miR-1249 | 36.5169 | 36.9969 | 33.3966 | 32.7972 | 31.4780 | . | 33.7038 | 31.8274 | 33.2639 | 36.6343 | 35.8326 | 32.8186 |
| bta-miR-1306 | 28.3227 | 28.2553 | 27.9370 | 28.7809 | 27.4888 | 29.1763 | 30.5620 | 26.5743 | 28.3839 | 30.5165 | 27.9403 | 28.0886 |
| bta-miR-1260b | 21.7134 | 21.9263 | 21.4981 | 23.3321 | 20.8133 | 22.8220 | 23.9149 | 21.4490 | 23.6837 | 22.5980 | 20.9101 | 22.7490 |
| bta-miR-1307 | 27.4050 | 27.7342 | 26.8718 | 29.1417 | 26.6454 | 28.6999 | 29.0824 | 26.5657 | 28.3840 | 28.8207 | 27.7509 | 27.7621 |
| bta-miR-1271 | 32.0955 | 32.6496 | 32.6037 | 31.8621 | 30.8371 | 32.6567 | . | 31.7353 | 31.8731 | 33.1223 | 32.8880 | 32.3137 |
| bta-miR-1343-3p | 28.4537 | 28.7484 | 28.1138 | 29.1592 | 26.8150 | 28.8178 | 29.9135 | 26.6676 | 28.7543 | 30.8361 | 28.0120 | 27.5741 |
| bta-miR-1277 | . | . | . | . | . | . | . | . | . | . | . | . |
| bta-miR-1343-5p | 24.4095 | 24.7643 | 23.4090 | 25.3219 | 22.8058 | 25.0283 | 26.8777 | 23.0432 | 24.7250 | 25.4799 | 24.5666 | 24.0330 |
| bta-miR-1281 | 28.2479 | 29.1126 | 28.0323 | 29.1958 | 25.8625 | 28.4084 | 31.8789 | 26.7124 | 28.1884 | 30.0557 | 29.7884 | 26.5853 |
| bta-miR-1388-3p | 30.1853 | 29.8920 | 29.6897 | 30.3395 | 28.7682 | 29.8903 | 31.8528 | 28.2345 | 30.1575 | 30.7431 | 30.3377 | 28.9773 |
| bta-miR-1282 | . | . | 36.9771 | 35.7403 | 32.8224 | . | . | 35.3923 | 35.0761 | . | 34.6061 | 35.0535 |
| RNT43 snoRNA | 29.7641 | 30.1469 | 29.2112 | 30.7354 | 29.6725 | 30.6002 | 30.4378 | 28.8917 | 30.7754 | 31.7674 | 30.0056 | 30.7444 |
| bta-miR-1284 | 33.9575 | . | 34.5083 | . | 30.8129 | 35.9108 | . | 32.2943 | . | . | . | 32.2890 |
| Hm/Ms/Rt T1 snRNA | 13.8171 | 13.5662 | 13.2898 | 13.7441 | 13.3647 | 14.0554 | 15.7783 | 12.7134 | 13.4337 | 14.3951 | 14.2455 | 13.7318 |
| bta-miR-1287 | . | . | . | . | 32.1608 | . | . | 34.3599 | . | . | . | 33.8545 |
| bta-miR-99b | 23.8166 | 23.7110 | 23.8340 | 23.7415 | 24.7679 | 23.7172 | 23.5108 | 24.6369 | 24.0899 | 22.9006 | 23.7781 | 23.9145 |
| bta-miR-1291 | 31.6743 | . | 33.5925 | 33.2988 | 30.6159 | 33.5334 | 33.9159 | 31.6542 | 32.9333 | 33.4315 | 33.9577 | 31.4523 |
| Negative control | . | . | . | . | . | . | . | . | . | . | . | . |
| ^1^Body energy reserve: MBER: Cows with moderated body energy reserve; HBER: Cows with high body energy reserve. | | | | | | | | | | | | |
