# Supplementary material for: High body energy reserve influences extracellular vesicles miRNA contents within the ovarian follicle
Source: PLoS One. 2023 Jan 10;18(1):e0280195. doi: 10.1371/journal.pone.0280195 (PMC9831338; doi:10.1371/journal.pone.0280195)
Supplement: S5 Table — (DOCX) [file pone.0280195.s008.docx]

| **Supplementary table 5.** Normalized data of the 6 exclusives miRNAs detected in follicular fluid extracellular vesicles (EV FF) compared to cumulus cells (CC) from ipsi and contralateral ovarian follicles (3-6 mm in diameter) from cows with moderated body energy reserve (MBER) | | | | | | | | | | | | | | | | | |
| --- | --- | --- | --- | --- | --- | --- | --- | --- | --- | --- | --- | --- | --- | --- | --- | --- | --- |
| miRNA | **MBER^1^** | | | | | | | | | | | | | | | | |
|  | **CC^2^** | | | | | | | | **EV FF^3^** | | | | | | | | |
|  | **1** | **2** | **3** | **4** | **5** | **6** | **7** | **8** | | **1** | **2** | **3** | **4** | **5** | **6** | **7** | **8** |
| bta-miR-181c | . | . | . | . | . | . | . | . | | 8,132996642 | 8,640025529 | 6,427796566 | 8,429802773 | 4,945081669 | 11,55921334 | 10,30181512 | 4,39011904 |
| bta-miR-193a-3p | . | . | . | . | . | . | . | . | | 10,25194653 | 9,667453203 | 7,956708157 | 10,37610328 | 5,772487599 | 12,5357644 | 10,45109949 | 4,847768271 |
| bta-miR-193b | . | . | . | . | . | . | . | . | | 5,67589482 | 5,991000566 | 5,730170452 | 6,656687615 | 3,642534214 | 6,50828332 | 8,976314637 | 2,811650717 |
| bta-miR-29d-3p | . | . | . | . | . | . | . | . | | 5,887381637 | 5,150321397 | 4,974793636 | 5,092440483 | 3,176912266 | 6,241519633 | 9,338638398 | 2,93126055 |
| bta-miR-1248 | . | . | . | . | . | . | . | . | | 7,95066365 | 8,956679735 | 7,979468548 | 8,333615181 | 7,066070515 | 9,949098292 | 9,245575043 | 7,087476218 |
| bta-miR-1249 | . | . | . | . | . | . | . | . | | 12,57262143 | 13,26765004 | 9,572621548 | 8,850003121 | 6,636095005 | 13,53172845 | 10,1273118 | 7,139872992 |
| ^1^MBER: Cows with moderated body energy reserve. ^2^CC: Cumulus cells. ^3^EV FF: Follicular fluid extracellular vesicles. | | | | | | | | | | | | | | | | | |
