# Supplementary material for: High body energy reserve influences extracellular vesicles miRNA contents within the ovarian follicle
Source: PLoS One. 2023 Jan 10;18(1):e0280195. doi: 10.1371/journal.pone.0280195 (PMC9831338; doi:10.1371/journal.pone.0280195)
Supplement: S6 Table — (DOCX) [file pone.0280195.s009.docx]

| **Supplementary table 6.** Normalized data of the 79 miRNAs commonly detected in cumulus cells (CC) and follicular fluid extracellular vesicles (EV FF) from ipsi and contralateral ovarian follicles (3-6 mm in diameter) from cows with moderated body energy reserve (MBER). | | | | | | | | | | | | | | | | | |
| --- | --- | --- | --- | --- | --- | --- | --- | --- | --- | --- | --- | --- | --- | --- | --- | --- | --- |
| **miRNA** | **MBER** | | | | | | | | | | | | | | | | ***P* - value** |
|  | **CC** | | | | | | | | **EV FF** | | | | | | | |  |
|  | **1** | **2** | **3** | **4** | **5** | **6** | **7** | **8** | **1** | **2** | **3** | **4** | **5** | **6** | **7** | **8** |  |
| bta-let-7a-5p | 3.215098 | 12.30125 | 13.60788 | . | 4.433421 | 5.613194 | . | -0.02804 | 2.792211 | 2.500034 | 2.091621 | 3.295899 | 0.728554 | 3.406233 | 6.609068 | 1.089246 | **0.0338** |
| bta-let-7b | 6.070368 | . | 13.75444 | . | 5.897568 | 7.516566 | . | 1.259287 | 1.632321 | 1.011032 | 1.408923 | 1.814925 | -0.09545 | 1.889122 | 3.42285 | -0.2337 | **0.024** |
| bta-let-7c | 3.733269 | 10.07284 | 13.06554 | . | 4.298087 | 5.772217 | 11.30433 | 0.190758 | 1.858687 | 1.416274 | 1.355439 | 2.275935 | 0.198864 | 2.177686 | 4.073399 | -0.0787 | **0.008933** |
| bta-miR-106a | 8.645741 | . | . | . | 9.872654 | 13.6011 | 12.25613 | 6.842761 | 1.825025 | 1.29792 | 0.521371 | 0.786946 | -0.70592 | 2.548731 | 5.986717 | -2.09807 | **6.28E-05** |
| bta-let-7d | 4.931174 | 13.75596 | . | . | 6.043734 | 7.087807 | . | 1.398373 | 4.562907 | 4.026845 | 3.764344 | 5.502978 | 2.124357 | 5.18478 | 8.110502 | 2.385335 | 0.244555 |
| bta-let-7e | 3.716477 | 10.73381 | 11.13868 | 12.43376 | 3.735995 | 4.130852 | 10.21619 | -0.42136 | 1.940122 | 1.984991 | 1.844918 | 2.858857 | 0.47064 | 2.79898 | 5.01787 | 1.09016 | **0.0313** |
| bta-let-7g | 4.17034 | 11.10507 | . | . | 7.010861 | 11.93923 | . | 3.441893 | 6.352487 | 4.863756 | 3.962333 | 5.007933 | 2.156702 | 7.123443 | 10.32924 | 1.980941 | 0.23227 |
| bta-miR-125a | 4.139088 | . | 14.40224 | 12.90915 | 9.095719 | 8.734744 | 11.82673 | 3.77657 | 5.828084 | 6.60202 | 5.917001 | 5.783868 | 2.938395 | 6.757005 | 10.09167 | 3.413862 | 0.067123 |
| bta-miR-125b | 6.920321 | . | 15.65969 | . | 11.74343 | 10.22754 | . | 6.507939 | 4.338666 | 2.87329 | 2.997707 | 2.650128 | 1.646759 | 3.707622 | 7.143185 | 1.064374 | **0.000954** |
| bta-miR-127 | 11.13453 | 11.63901 | 13.01234 | 11.44861 | 9.207448 | 10.01111 | 9.854611 | 7.672476 | 6.772437 | 8.156606 | 7.369352 | 8.067426 | 5.716337 | 8.484728 | 8.799908 | 5.719072 | **0.000745** |
| bta-miR-130a | 10.48232 | 11.17032 | 10.55417 | 10.9576 | 10.04546 | 9.398013 | 8.682341 | 9.408354 | 5.695558 | 5.118623 | 4.700797 | 5.923238 | 3.163383 | 6.750129 | 6.421372 | 1.313138 | **4.04E-06** |
| bta-miR-130b | 7.179871 | 9.8964 | 10.57661 | 9.839933 | 9.256295 | 9.267138 | 8.173609 | 7.682715 | 6.022334 | 6.114021 | 4.925943 | 6.665379 | 3.285904 | 6.830455 | 8.19878 | 2.033929 | **0.000876** |
| bta-miR-143 | 9.392117 | 11.52765 | . | 12.8493 | 10.26438 | 11.01025 | 8.680583 | 10.4541 | 5.560336 | 7.024858 | 6.73507 | 6.808034 | 4.996136 | 8.246916 | 8.967114 | 4.322917 | **0.000167** |
| bta-miR-154b | . | 11.53787 | 11.59966 | 8.428284 | 9.205147 | 10.48119 | 6.006369 | 6.270384 | 3.326585 | 9.111633 | 9.129033 | 8.43895 | 9.059008 | 8.500227 | 8.509853 | 7.184246 | 0.308318 |
| bta-miR-155 | 8.249493 | . | . | . | 7.841656 | 10.00446 | 10.26027 | 8.502276 | 5.942416 | 6.651609 | 6.885223 | 6.81046 | 4.570038 | 7.390615 | 8.677853 | 4.807456 | **0.005067** |
| bta-miR-149-3p | 9.753766 | . | . | 12.54941 | 10.54814 | 10.17445 | 9.197259 | 7.350993 | -0.70452 | -0.30188 | -1.50298 | 0.856118 | -1.46898 | 0.585699 | 2.630514 | -2.22775 | **0.0024** |
| bta-miR-15b | 4.186984 | . | 12.6867 | . | 9.147881 | 7.770051 | . | 4.263352 | 2.818261 | 2.704601 | 2.042325 | 2.594123 | 0.452984 | 3.398721 | 6.119854 | -0.23235 | **0.005963** |
| bta-miR-16b | 5.258654 | . | . | . | 8.716912 | 10.33057 | 9.370656 | 5.331124 | 0.891456 | 0.641147 | -0.0684 | 0.675811 | -1.22391 | 1.609184 | 5.035885 | -2.45406 | **0.00017** |
| bta-miR-181d | 8.944932 | 12.12964 | . | 13.03332 | 10.21073 | 12.15633 | 9.639542 | 8.298541 | 4.434524 | 5.433212 | 5.857043 | 5.606727 | 3.876179 | 5.766222 | 7.956472 | 3.703428 | **2.17E-05** |
| bta-miR-191 | 7.095452 | -3.59594 | 6.564763 | -3.71136 | 7.982953 | 10.52639 | 8.861451 | 5.974104 | 4.037101 | 4.181549 | 4.866939 | 4.853616 | 2.930382 | 5.771816 | 11.72113 | 1.714357 | 0.983223 |
| bta-miR-193a-5p | 12.34878 | . | 15.5044 | . | 9.215027 | 9.074168 | . | 6.21794 | 3.969068 | 4.78734 | 3.931789 | 4.571732 | 2.484171 | 4.253895 | 5.077896 | 1.675167 | **0.000421** |
| bta-miR-195 | 5.854579 | 10.72583 | 14.68337 | 14.03793 | 9.69439 | 9.691508 | 8.853671 | 4.906474 | 2.834575 | 2.426984 | 1.902732 | 2.495082 | 0.873251 | 3.327295 | 7.118233 | 0.012599 | **0.000185** |
| bta-miR-196a | 10.27676 | . | . | 12.95908 | 9.145028 | . | 8.905602 | 7.410037 | 4.825387 | 10.17948 | 9.037694 | 9.59137 | 9.037527 | 8.799249 | . | 7.403648 | 0.262127 |
| bta-miR-197 | 9.400731 | . | . | . | 5.665461 | 5.712979 | 7.058657 | 1.52106 | 3.777915 | 5.166643 | 4.293205 | 4.83055 | 1.935633 | 4.64139 | 8.514645 | 1.935298 | 0.30031 |
| bta-miR-20a | 7.088828 | . | . | 12.97966 | 9.760335 | . | 10.6504 | 6.669123 | 1.371223 | 1.107347 | 0.12717 | 0.849587 | -1.04604 | 2.169386 | 4.212929 | -2.50942 | **3.3E-05** |
| bta-miR-214 | 11.11059 | . | . | . | 10.523 | 11.11479 | 13.56348 | 10.8706 | 4.665731 | 5.117277 | 4.557758 | 5.996772 | 2.941776 | 4.953314 | 7.56877 | 2.662439 | **6.5E-06** |
| bta-miR-23a | 5.279618 | . | . | 12.39326 | 10.50482 | 9.984946 | 10.80466 | 7.294737 | 0.372029 | 0.13577 | 0.618196 | 0.915259 | -1.14219 | 1.282781 | 2.95772 | -1.27693 | **0.0024** |
| bta-miR-23b-3p | 5.757338 | . | . | . | 10.11889 | 9.634185 | 9.83989 | 6.672854 | 2.81582 | 3.39079 | 3.110194 | 4.133073 | 1.699387 | 3.650545 | 6.145268 | 2.007789 | **0.000231** |
| bta-miR-219 | 11.54266 | 13.33979 | 14.67208 | . | 11.71203 | . | 8.524886 | 9.907771 | 4.770383 | 8.177511 | 8.041068 | 8.333371 | 5.666271 | 8.346689 | 10.42165 | 5.705604 | **0.002464** |
| bta-miR-22-3p | -22.3943 | -20.9282 | -18.8748 | -19.378 | -22.2366 | -20.3166 | -20.4949 | -23.1439 | . | -20.4692 | -20.7923 | -20.978 | . | -21.179 | -20.6967 | -21.7863 | 0.984605 |
| bta-miR-26a | 3.878838 | . | . | 11.99011 | 8.557168 | 9.945662 | 9.16404 | 5.395619 | 2.747352 | 3.042995 | 2.719577 | 4.342561 | 0.960012 | 4.336471 | 8.24567 | 1.461111 | **0.005972** |
| bta-miR-27a-5p | -15.1022 | -13.9998 | -13.16 | -13.4379 | -15.577 | 12.79619 | . | -18.1111 | . | 13.1862 | 10.15561 | -15.7153 | 7.323133 | 10.3995 | -14.8481 | 8.354232 | 0.2013 |
| bta-miR-296-3p | 8.207261 | 10.75866 | . | 13.5464 | 9.846413 | 10.44479 | 12.22404 | 5.482996 | 1.795016 | 2.006643 | 1.71063 | 2.408902 | 0.239622 | 7.864483 | 3.869486 | -0.43479 | **7.46E-05** |
| bta-miR-296-5p | 11.14247 | . | 15.51521 | . | . | 12.35511 | 9.819813 | 8.488772 | 5.077575 | 7.664756 | 6.928815 | 7.849133 | 4.045095 | 7.566004 | . | 3.785406 | **0.001934** |
| bta-miR-30a-5p | 8.036607 | . | . | 12.45754 | . | 13.94876 | 11.87715 | 6.555602 | 5.47878 | 5.203781 | 4.354104 | 5.035621 | 2.076914 | 6.763138 | 9.33358 | 1.053235 | **0.004509** |
| bta-miR-30b-5p | 8.265313 | . | . | 12.98337 | . | 12.24184 | 9.751958 | 7.770228 | 6.362189 | 7.672992 | 6.29627 | 7.650136 | 4.904612 | 7.781374 | 10.25624 | 3.725513 | **0.017781** |
| bta-miR-30f | 7.485158 | . | . | 11.86089 | . | 10.53886 | 12.06656 | 7.722334 | 5.860526 | 7.337415 | 6.07225 | 7.44496 | 4.262798 | 7.81147 | 10.17448 | 3.716421 | **0.018339** |
| bta-miR-31 | 5.874842 | . | . | 14.86616 | 8.75589 | 8.908318 | . | 4.646374 | 4.768216 | 5.027551 | 4.060386 | 5.857175 | 2.924675 | 5.843519 | 7.993902 | 2.03808 | **0.036934** |
| bta-miR-320a | 7.322499 | 13.05963 | 13.38779 | . | 6.773951 | 8.9854 | 11.28365 | 2.983008 | -2.42618 | -1.99377 | -1.98459 | -1.25187 | -3.11454 | -1.2989 | -0.79841 | -4.03383 | **0.0015** |
| bta-miR-323 | -5.18944 | -6.11715 | -5.38978 | -6.20306 | -5.28123 | -5.59549 | -5.46139 | -4.83251 | -4.40998 | -4.33718 | -4.35565 | -4.8366 | -4.38625 | -4.32944 | -4.75513 | -4.17489 | **4.29E-05** |
| bta-miR-339b | 8.4637 | . | . | 11.56432 | 6.8221 | 12.27246 | 8.911553 | 5.739888 | 2.611194 | 2.634731 | 2.828641 | 4.867333 | 0.714785 | 3.740152 | 5.028696 | -0.03264 | **0.000186** |
| bta-miR-342 | 9.401555 | . | . | 13.04076 | . | 10.03609 | 10.51138 | 6.184248 | 5.997533 | 7.388938 | 6.139539 | 7.486733 | 4.63203 | 7.235466 | 10.44154 | 3.551801 | **0.027498** |
| bta-miR-370 | . | 12.51423 | . | 12.64842 | 9.221791 | 9.303839 | 11.81812 | 7.566905 | 8.4871 | 8.004922 | 7.36024 | 7.879529 | 4.316734 | 8.421587 | 8.706871 | 4.768024 | **0.007583** |
| bta-miR-361 | 7.342177 | . | . | . | 9.279233 | 9.544818 | . | 5.987904 | 4.072736 | 3.965442 | 2.315799 | 3.290706 | 1.927412 | 4.435362 | 5.200892 | 1.117671 | **0.00039** |
| bta-miR-375 | . | 11.53151 | 11.71266 | 8.648683 | 9.731667 | 8.885255 | 7.494181 | . | 5.279582 | 4.990886 | 6.331535 | 7.412812 | 5.227915 | 7.370493 | 8.994206 | 4.922 | **0.001897** |
| bta-miR-382 | 11.98994 | 11.69393 | 14.89399 | 10.75571 | 9.272305 | 11.59744 | 8.573325 | 7.601004 | 4.783656 | 6.072122 | 6.473855 | 6.76213 | 4.986256 | 6.805132 | 7.261768 | 4.147624 | **9.59E-05** |
| bta-miR-411a | . | 12.7237 | 15.52795 | 12.44741 | 9.937943 | 12.14102 | 9.117055 | 10.15451 | 4.829652 | 6.978671 | 8.587049 | 7.956605 | 6.214401 | 8.112974 | 9.126362 | 5.842596 | **0.00039** |
| bta-miR-411b | . | . | 13.23535 | 13.33188 | 10.82286 | 10.91266 | 11.02311 | . | 7.743793 | 10.50255 | 9.673749 | 11.1927 | . | 11.37013 | 10.79482 | 12.2342 | 0.12501 |
| bta-miR-421 | 9.170861 | 11.24145 | 11.37545 | 12.64928 | 7.199696 | . | 8.205014 | 9.701048 | 3.708911 | 4.616867 | 3.976026 | 5.132626 | 2.932148 | 5.717897 | 6.065451 | 2.141521 | **1.62E-05** |
| bta-miR-425-3p | 7.904435 | . | . | . | 11.21317 | 7.867654 | 10.17831 | 7.348769 | 2.798703 | 3.996406 | 1.623958 | 4.152711 | 0.929003 | 4.398025 | 3.996986 | -0.00152 | **5.07E-05** |
| bta-miR-429 | 11.39323 | 9.009559 | 12.35634 | 10.70446 | 7.483674 | 7.672282 | 7.183665 | 7.667635 | 5.257691 | 7.786155 | 7.765954 | 6.823909 | 7.036821 | 7.576517 | 7.219162 | 5.631647 | **0.0313** |
| bta-miR-433 | 7.325066 | 7.170512 | 7.541068 | 7.413553 | 8.129597 | 8.039977 | 7.088624 | 8.066107 | 3.869744 | 8.121217 | 8.039245 | 7.916556 | 4.92769 | 7.665172 | 9.215801 | 5.865701 | 0.352673 |
| bta-miR-486 | 9.060658 | . | . | . | 11.61433 | 9.9136 | 11.81209 | 6.797068 | 4.300363 | 5.475776 | 3.953422 | 5.979155 | 2.278978 | 4.920465 | 6.571297 | 2.073749 | **0.00027** |
| bta-miR-489 | . | 11.69538 | . | 13.26299 | 10.26278 | 11.364 | 11.07234 | . | 8.982335 | 11.47153 | 11.58797 | . | 10.70668 | 11.73388 | 11.72618 | 9.86341 | 0.321695 |
| bta-miR-503-3p | 9.837628 | . | . | 12.91956 | 9.735543 | 9.629145 | 8.446162 | 5.450907 | 3.435557 | 5.086625 | 4.726762 | 5.606315 | 2.444521 | 5.24382 | 8.161388 | 2.680282 | 0.001473 |
| bta-miR-494 | 3.777386 | 7.594819 | 8.55856 | 8.500681 | 4.0861 | 2.965115 | 6.821002 | 1.901948 | -0.2483 | 0.168199 | -1.11696 | 1.89335 | -2.18053 | 1.122631 | 1.966812 | -2.19289 | **0.000172** |
| bta-miR-541 | . | 10.59339 | 13.40863 | 11.92196 | 9.387696 | 9.454722 | 8.790703 | 8.786826 | 5.819163 | 8.977477 | 7.976114 | 7.931903 | 4.698671 | 7.176092 | 9.32465 | 5.298851 | **0.003557** |
| bta-miR-615 | -12.4242 | -11.7471 | -9.67773 | -10.6278 | -12.9855 | -11.0878 | -11.4055 | -14.1247 | -10.9934 | -10.8435 | -11.0549 | -11.1816 | -12.2088 | -11.254 | -10.8003 | -11.9627 | 0.385984 |
| bta-miR-631 | -2.26941 | -3.50954 | -2.29511 | -2.97419 | -3.38003 | -3.29589 | -3.76566 | -3.04582 | -3.34991 | -3.28768 | -3.23133 | -3.53924 | -3.17679 | -3.56914 | -3.58642 | -3.49204 | 0.115493 |
| bta-miR-574 | 6.507865 | 9.833259 | 11.85414 | 10.87736 | 3.815402 | 4.638098 | 8.299063 | 1.05069 | -0.92311 | -0.31558 | -1.23611 | -1.1116 | -4.23913 | -1.34568 | 2.86277 | -3.08695 | **8.68E-05** |
| bta-miR-656 | . | 10.64125 | 11.39116 | 10.7835 | 9.566304 | 10.78281 | 9.521733 | 10.22771 | 7.369395 | 12.98711 | 10.4122 | 11.81509 | 10.87819 | 11.45726 | 11.44767 | 11.02843 | 0.457325 |
| bta-miR-664b | 7.382029 | . | . | . | 9.581193 | 11.07551 | 11.83138 | 8.720412 | 7.124858 | 6.445125 | 7.391444 | 8.637925 | 5.002111 | 8.282869 | 10.14012 | 5.371532 | **0.033071** |
| bta-miR-760-5p | 7.270047 | . | . | 12.1605 | 8.748971 | 10.04024 | 7.589881 | 5.905694 | 3.877118 | 6.044126 | 5.29326 | 5.7771 | 2.629333 | 5.37866 | 8.082407 | 3.234371 | **0.005499** |
| bta-miR-669 | . | . | . | 13.62011 | 7.070479 | 8.899525 | 8.378209 | 5.404095 | 1.754928 | 2.860008 | 1.552438 | 1.878007 | -1.89141 | 1.458511 | 6.242317 | -0.87491 | **0.000761** |
| bta-miR-767 | 12.20366 | 10.7186 | 11.55729 | 11.12455 | 10.24837 | 9.196742 | 9.453023 | 8.463755 | 8.68641 | 10.18311 | 9.902524 | 9.933469 | 7.147308 | 10.18529 | 9.573742 | 8.098186 | **0.074135** |
| bta-miR-744 | 9.385419 | 10.55524 | 14.59561 | . | 5.995921 | 7.785836 | 11.8831 | 4.808747 | 3.375123 | 4.050882 | 3.733791 | 5.021052 | 2.418705 | 4.071747 | 5.069756 | 1.900695 | **0.000734** |
| bta-miR-92a | 4.735291 | . | . | 11.5883 | . | 9.754355 | 10.67486 | 4.063365 | -1.46642 | -1.529 | -1.82673 | -1.30758 | -3.13645 | -1.19257 | 1.025005 | -4.02321 | **0.0043** |
| bta-miR-92b | 8.573891 | 9.73891 | 11.63017 | 10.92478 | 7.875081 | 7.745425 | 7.948674 | 5.834479 | 2.867364 | 3.043954 | 2.338641 | 3.300725 | 0.544138 | 3.464012 | 5.056294 | 0.123144 | **5.57E-06** |
| bta-miR-940 | 7.838727 | . | . | 14.92041 | 10.10513 | 9.030413 | . | 6.511315 | 1.369868 | 0.038812 | 0.219827 | 0.871463 | -1.02252 | 1.721397 | 3.139744 | -1.29735 | **2.26E-05** |
| bta-miR-1224 | 9.713971 | . | . | 12.50093 | 8.747124 | 12.04413 | 10.46045 | 6.203114 | -4.73316 | -5.04153 | -5.25307 | -3.73504 | -5.63507 | -3.5647 | -2.86183 | -6.76774 | **0.0024** |
| bta-miR-1225-3p | 9.292535 | . | . | . | 9.17725 | 9.087702 | 12.38294 | 7.557841 | 2.72219 | 3.834461 | 2.430978 | 3.427966 | -0.01476 | 2.840507 | 6.570076 | 0.669569 | **7.72E-05** |
| bta-miR-1246 | 2.807593 | 6.842181 | 7.515875 | 6.950517 | 1.879206 | 2.675608 | 7.424275 | -1.65139 | -8.74468 | -8.99259 | -10.0009 | -9.79199 | -10.1175 | -9.85181 | -6.77818 | -10.8613 | **0.0009** |
| bta-miR-1247-3p | . | . | 12.40905 | 9.753433 | 9.182152 | 12.12637 | 8.512841 | 8.228522 | 4.171949 | 6.700197 | 4.906282 | 6.504491 | 2.977133 | 5.145967 | 8.119667 | 3.35868 | **0.000324** |
| bta-miR-1306 | 9.367711 | . | . | . | 9.732987 | 11.81713 | 8.982804 | 7.141501 | 4.378354 | 4.526006 | 4.112977 | 4.833623 | 2.646875 | 5.165622 | 6.985487 | 1.886833 | **0.000164** |
| bta-miR-1260b | 2.622735 | 13.1488 | 10.80618 | 13.16521 | 5.491093 | 5.377198 | 9.715718 | 0.980614 | -2.23093 | -1.80295 | -2.32592 | -0.61509 | -4.02855 | -1.18863 | 0.338402 | -3.23851 | **7.69E-05** |
| bta-miR-1307 | 9.742581 | 12.71238 | . | 12.95624 | 11.43699 | 10.05981 | 12.27567 | 7.590617 | 3.460687 | 4.004888 | 3.047805 | 5.19447 | 1.803564 | 4.689272 | 5.505934 | 1.87824 | **1.36E-06** |
| bta-miR-1343-5p | 8.258378 | . | . | 13.61593 | 9.282803 | 10.29994 | . | 7.232092 | 0.465129 | 1.035032 | -0.41497 | 1.374661 | -2.03604 | 1.017648 | 3.301182 | -1.64427 | **5.72E-06** |
| Hm/Ms/Rt T1 snRNA | -1.90941 | 5.861194 | 8.170708 | 5.453375 | 3.009621 | 4.20506 | 7.079884 | 0.150317 | -10.1272 | -10.1631 | -10.5341 | -10.2031 | -11.4772 | -9.95522 | -7.79816 | -11.974 | **2.74E-08** |
| bta-miR-99b | -0.03187 | -0.06523 | 0.103253 | -0.2149 | -0.02333 | -0.00564 | -0.09056 | -0.14842 | -0.12772 | -0.01831 | 0.010028 | -0.20575 | -0.07401 | -0.29346 | -0.06565 | -0.05059 | 0.392802 |
| ^1^MBER: Cows with moderated body energy reserve. ^2^CC: Cumulus cells. ^3^EV FF: Follicular fluid extracellular vesicles. | | | | | | | | | | | | | | | | | |
