# Supplementary material for: High body energy reserve influences extracellular vesicles miRNA contents within the ovarian follicle
Source: PLoS One. 2023 Jan 10;18(1):e0280195. doi: 10.1371/journal.pone.0280195 (PMC9831338; doi:10.1371/journal.pone.0280195)
Supplement: S7 Table — (DOCX) [file pone.0280195.s010.docx]

| **Supplementary table 7.** Normalized data of the 42 exclusives miRNAs detected in follicular fluid extracellular vesicles (EV FF) compared to cumulus cells (CC) from ipsi and contralateral ovarian follicles (3-6 mm in diameter) from cows with high body energy reserve (HBER). | | | | | | | | |
| --- | --- | --- | --- | --- | --- | --- | --- | --- |
| **miRNA** | **HBER^1^** | | | | | | | |
|  | **CC^2^** | | | | **EV FF^3^** | | | |
|  | **1** | **2** | **3** | **4** | **1** | **2** | **3** | **4** |
|  |  |  |  |  |  |  |  |  |
| bta-let-7a-3p | . | . | . | . | 8.840998 | 9.681344 | 8.980979 | 10.08494 |
| bta-miR-103 | . | . | . | . | 6.730314 | 8.033536 | 6.926074 | 6.826751 |
| bta-miR-124b | . | . | . | . | 9.831537 | 12.44323 | 12.84489 | 10.04279 |
| bta-miR-135a | . | . | . | . | 9.775423 | 15.06291 | 11.45315 | 10.10634 |
| bta-miR-129 | . | . | . | . | 7.736664 | 11.30417 | 11.11477 | 6.042321 |
| bta-miR-129-5p | . | . | . | . | 8.539625 | 10.07807 | 11.06834 | 6.772817 |
| bta-miR-139 | . | . | . | . | 8.921545 | 10.82945 | 7.902148 | 8.970344 |
| bta-miR-151-3p | . | . | . | . | 3.858425 | 6.132859 | 4.871276 | 4.346385 |
| bta-miR-15a | . | . | . | . | 1.153528 | 4.683518 | 3.199073 | 1.549346 |
| bta-miR-18a | . | . | . | . | 5.172365 | 9.349324 | 7.858508 | 5.529571 |
| bta-miR-192 | . | . | . | . | 7.721663 | 10.44714 | 7.999454 | 6.931186 |
| bta-miR-185 | . | . | . | . | 6.574846 | 8.272486 | 6.327161 | 5.822673 |
| bta-miR-199a-3p | . | . | . | . | 1.957819 | 5.021876 | 4.350421 | 2.790271 |
| bta-miR-199b | . | . | . | . | 9.117686 | 11.54005 | 10.1803 | 9.093337 |
| bta-miR-21-3p | . | . | . | . | 8.794981 | 13.80871 | 8.973876 | 8.268748 |
| bta-miR-22-5p | . | . | . | . | 7.086943 | 10.85125 | 7.956489 | 8.329776 |
| bta-miR-221 | . | . | . | . | 3.345238 | 5.67771 | 3.547368 | 3.888532 |
| bta-miR-224 | . | . | . | . | 5.889757 | 7.51636 | 6.166968 | 5.845554 |
| bta-miR-23b-5p | . | . | . | . | 10.84016 | 12.71617 | 8.324142 | 10.01335 |
| bta-miR-29d-3p | . | . | . | . | 4.650437 | 7.606301 | 6.319762 | 4.728004 |
| bta-miR-28 | . | . | . | . | 7.611969 | 10.6689 | 8.432037 | 7.701196 |
| bta-miR-326 | . | . | . | . | 4.664486 | 6.811711 | 5.941442 | 3.507463 |
| bta-miR-365-3p | . | . | . | . | 4.623163 | 8.718238 | 6.797076 | 4.432094 |
| bta-miR-345-3p | . | . | . | . | 5.114012 | 6.7555 | 5.791398 | 4.928218 |
| bta-miR-423-3p | . | . | . | . | 2.025847 | 4.772738 | 2.644385 | 2.695981 |
| bta-miR-424-3p | . | . | . | . | 5.098479 | 7.869809 | 4.458875 | 5.506824 |
| bta-miR-424-5p | . | . | . | . | -1.41587 | 2.270188 | -0.15801 | -0.56691 |
| bta-miR-450b | . | . | . | . | 8.386999 | 11.41592 | 10.8639 | 8.0279 |
| bta-miR-425-5p | . | . | . | . | 3.843693 | 5.81767 | 4.557492 | 4.793695 |
| bta-miR-451 | . | . | . | . | 7.866064 | 11.63917 | 10.95129 | 9.485839 |
| bta-miR-452 | . | . | . | . | 12.45491 | 10.55729 | 9.665935 | 11.0182 |
| bta-miR-431 | . | . | . | . | 12.24971 | 11.64742 | 10.05683 | 6.898252 |
| bta-miR-500 | . | . | . | . | 6.411852 | 8.785857 | 6.134647 | 6.553189 |
| bta-miR-502b | . | . | . | . | 5.276325 | 7.787417 | 6.242675 | 6.046933 |
| bta-miR-490 | . | . | . | . | 8.803171 | 12.01421 | 10.28654 | 6.924419 |
| bta-miR-504 | . | . | . | . | 10.66615 | 10.58048 | 6.664054 | 6.689752 |
| bta-miR-542-5p | . | . | . | . | 7.629019 | 12.1712 | 9.278518 | 10.94751 |
| bta-miR-584 | . | . | . | . | 6.584555 | 8.074359 | 8.534057 | 5.227552 |
| bta-miR-652 | . | . | . | . | 5.207606 | 7.443011 | 5.225867 | 6.125333 |
| bta-miR-671 | . | . | . | . | 6.472719 | 8.509576 | 6.736825 | 6.971238 |
| bta-miR-708 | . | . | . | . | 8.646452 | 10.79121 | 8.088996 | 8.008893 |
| bta-miR-874 | . | . | . | . | 5.621453 | 7.232259 | 6.782098 | 4.615428 |
| ^1^HBER: Cows with high body energy reserve. ^2^CC: Cumulus cells. ^3^EV FF: Follicular fluid extracellular vesicles. | | | | | | | | |
