# Supplementary material for: High body energy reserve influences extracellular vesicles miRNA contents within the ovarian follicle
Source: PLoS One. 2023 Jan 10;18(1):e0280195. doi: 10.1371/journal.pone.0280195 (PMC9831338; doi:10.1371/journal.pone.0280195)
Supplement: S8 Table — (DOCX) [file pone.0280195.s011.docx]

| **Supplementary table 8**. Normalized data of the 46 miRNAs commonly detected in cumulus cells (CC) and follicular fluid extracellular vesicles (EV FF) from ipsi and contralateral ovarian follicles (3-6 mm in diameter) from cows with high body energy reserve (HBER). | | | | | | | | | |
| --- | --- | --- | --- | --- | --- | --- | --- | --- | --- |
| **miRNA** | **HBER^1^** | | | | | | | | ***P* - value** |
|  | **CC^2^** | | | | **EV FF^3^** | | | |  |
|  | **1** | **2** | **3** | **4** | **1** | **2** | **3** | **4** |  |
| bta-let-7a-5p | 13.60788 | 10.1573 | 5.371198 | . | 3.017429564 | 4.058363635 | 3.677334923 | 2.787291723 | 0.0518 |
| bta-let-7c | 13.06554 | 10.65505 | 4.890073 | 11.22076 | 1.322870467 | 3.161875445 | 1.495160241 | 1.74738999 | 0.004460785 |
| bta-miR-125b | 15.65969 | 9.812956 | 10.45917 | . | 1.815378401 | 4.580431659 | 3.867631096 | 2.901833731 | 0.003773581 |
| bta-miR-127 | 13.01234 | . | 10.42745 | 9.776014 | 8.785116408 | 9.975571353 | 7.951077599 | 6.861024099 | 0.064900645 |
| bta-miR-130a | 10.55417 | . | 9.090299 | 9.240465 | 5.125269149 | 7.141703326 | 4.825574059 | 5.188235479 | 0.002713422 |
| bta-miR-130b | 10.57661 | 11.48177 | 8.127817 | 7.995895 | 5.539304946 | 7.472420413 | 4.95099749 | 5.466145712 | 0.011980864 |
| bta-miR-133a | . | -1.60033 | 8.079141 | 10.22241 | 7.284696791 | . | 9.61900248 | 6.348824697 | 0.592955417 |
| bta-miR-154b | 11.59966 | . | 7.494424 | 6.596182 | 9.802572463 | 9.713112552 | 7.893300194 | 8.160681921 | 0.825885231 |
| bta-miR-16b | . | 9.53496 | 8.387182 | 9.216547 | -0.341666963 | 2.543822009 | 1.131016869 | 0.251124578 | 0.000151921 |
| bta-miR-191 | 6.564763 | -4.38082 | 8.387235 | -1.90635 | 4.70690367 | 7.455040652 | 6.608908791 | 4.76382752 | 0.289921261 |
| bta-miR-195 | 14.68337 | . | 9.871355 | 10.25303 | 1.6695101 | 4.44974013 | 3.148009438 | 1.9552414 | 0.002010797 |
| bta-miR-20a | . | 11.06374 | 9.069215 | 11.04984 | -0.012710359 | 2.844031054 | 1.344552178 | 0.576401907 | 0.00016824 |
| bta-miR-219 | 14.67208 | . | 6.938932 | 9.929029 | 8.078176711 | 11.34668799 | 8.807860512 | 6.976947027 | 0.468133315 |
| bta-miR-22-3p | -18.8748 | -21.7255 | -21.4024 | -21.2204 | . | -19.9657381 | -21.06183062 | -20.8403335 | 0.832247831 |
| bta-miR-26a | . | 8.387812 | 8.198869 | 9.142145 | 3.614025328 | 5.06586333 | 3.899377007 | 3.506255346 | 0.000236052 |
| bta-miR-27a-5p | -13.16 | -15.7191 | -14.8889 | -13.5949 | 13.50524733 | 5.518896777 | -13.08026109 | 7.995661932 | 0.0304 |
| bta-miR-296-5p | 15.51521 | . | 7.373966 | 9.116755 | 6.622432921 | 8.860003192 | 8.011187691 | 5.937955099 | 0.194959024 |
| bta-miR-30c | . | 9.139177 | 8.439081 | 10.6929 | 5.634832595 | 7.700289447 | 5.222193856 | 5.976788717 | 0.011850998 |
| bta-miR-320a | 13.38779 | . | 6.6058 | 10.17063 | -2.172078874 | -0.125914853 | -2.157404762 | -1.686190409 | 0.001137708 |
| bta-miR-323 | -5.38978 | -2.80919 | -5.73866 | -5.97797 | -4.669976976 | -4.776913922 | -4.826300483 | -4.585221094 | 0.73155422 |
| bta-miR-339b | . | -7.25706 | 7.492998 | 9.5287 | 3.254015181 | 5.363964755 | 2.263535637 | 3.435103613 | 0.945235508 |
| bta-miR-375 | 11.71266 | . | 6.326641 | 6.026993 | . | 8.880825717 | 6.857951302 | 5.879986959 | 0.710679355 |
| bta-miR-382 | 14.89399 | 11.88123 | 8.55459 | 9.474821 | 5.566110823 | 8.819950778 | 6.583375115 | 7.031323629 | 0.03684902 |
| bta-miR-411a | 15.52795 | . | 8.725126 | 9.133043 | 7.959509108 | 9.765904147 | 7.970257897 | 8.951308447 | 0.253754703 |
| bta-miR-411b | 13.23535 | -0.78591 | 10.73742 | 10.60206 | 11.9232733 | 9.576386172 | 10.01599421 | 10.10902329 | 0.560427126 |
| bta-miR-380-5p | . | 9.790529 | 6.994192 | 8.67872 | 8.737337325 | . | 11.52760233 | 7.701348501 | 0.583888098 |
| bta-miR-421 | 11.37545 | 10.3644 | 7.375608 | 10.15359 | 4.7224066 | 5.917767245 | 4.055590767 | 5.480668264 | 0.00239729 |
| bta-miR-429 | 12.35634 | 11.97043 | 7.617639 | 7.360996 | 7.036277983 | 8.200892169 | 6.738423485 | 7.413148123 | 0.124555599 |
| bta-miR-4523 | 14.61351 | . | 10.68509 | 11.80449 | 8.729601119 | 11.63163443 | 12.09932246 | 7.280131536 | 0.207806617 |
| bta-miR-433 | 7.541068 | 7.518377 | 6.114437 | 6.627138 | 7.349058364 | 8.498524386 | 8.875804085 | 5.923842626 | 0.37994595 |
| bta-miR-494 | 8.55856 | 6.387634 | 5.271242 | 7.027458 | 0.415170882 | 1.933998782 | -0.379731041 | -0.305174631 | 0.000326941 |
| bta-miR-532 | . | 9.610541 | 10.12644 | 10.17028 | 6.239338134 | 9.363980014 | 7.091916222 | 6.589635092 | 0.025888173 |
| bta-miR-541 | 13.40863 | . | 7.920755 | 8.268494 | 6.993602965 | 9.692154605 | 8.84834208 | 5.760682302 | 0.312636197 |
| bta-miR-615 | -9.67773 | -12.5695 | -12.8695 | -12.3829 | -11.41256502 | -10.52007608 | -10.68553148 | -11.07478408 | 0.260378394 |
| bta-miR-631 | -2.29511 | -0.4771 | -3.74077 | -3.79976 | -3.439851548 | -3.159705442 | -3.597781044 | -3.284222406 | 0.35340923 |
| bta-miR-574 | 11.85414 | 12.15933 | 4.437944 | 7.754669 | -1.326600816 | 1.144066531 | 0.809407372 | -3.049711985 | 0.003549646 |
| bta-miR-656 | 11.39116 | . | 8.968389 | 8.783125 | 9.776033614 | 13.46832724 | 11.32543101 | 10.34963532 | 0.258881244 |
| bta-miR-767 | 11.55729 | . | 10.46121 | 8.912027 | 8.744245742 | 11.29809256 | 8.341426033 | 8.667781073 | 0.357199391 |
| bta-miR-92b | 11.63017 | . | 5.349378 | 6.517881 | 2.637703154 | 4.78318854 | 3.379721779 | 1.833565908 | 0.046425672 |
| bta-miR-1224 | . | 11.03741 | 8.346044 | 11.79233 | -4.671678331 | -2.476556104 | -4.244444709 | -3.649300379 | 0.0518 |
| bta-miR-1246 | 7.515875 | 5.672995 | 3.160711 | 8.895163 | -10.12315443 | -8.762016576 | -8.593123298 | -10.07455043 | 0.0304 |
| bta-miR-1247-3p | 12.40905 | . | 7.525156 | 9.318415 | 5.555956099 | 7.85905524 | 5.827456612 | 4.784997183 | 0.046512931 |
| bta-miR-1260b | 10.80618 | 4.89451 | 5.671657 | 10.00764 | -0.485650804 | -0.400582593 | -2.985649925 | -1.102085871 | 0.001337021 |
| bta-miR-1307 | . | 12.33522 | 8.716535 | 10.06846 | 4.214714263 | 5.822113711 | 3.855122704 | 3.910992819 | 0.002328034 |
| Hm/Ms/Rt T1 snRNA | 8.170708 | 3.373505 | 1.700271 | 6.983095 | -10.7356489 | -8.603463452 | -9.650225502 | -10.11936168 | 8.27384E-05 |
| bta-miR-99b | 0.103253 | -0.17155 | -0.06163 | -0.05894 | -0.079404619 | -0.097962659 | -0.117640358 | 0.063430029 | 0.88376036 |
| ^1^HBER: Cows with high body energy reserve. ^2^CC: Cumulus cells. ^3^EV FF: Follicular fluid extracellular vesicles. | | | | | | | | | |
