# Supplementary material for: High body energy reserve influences extracellular vesicles miRNA contents within the ovarian follicle
Source: PLoS One. 2023 Jan 10;18(1):e0280195. doi: 10.1371/journal.pone.0280195 (PMC9831338; doi:10.1371/journal.pone.0280195)
Supplement: S9 Table — (DOCX) [file pone.0280195.s012.docx]

| **Supplementary table 9.** Biological patwhays predicted as modulated by exclusives miRNAs detected in follicular fluid extracellular vesicles (EV FF) compared to cumulus cells (CC) from ipsi and contralateral ovarian follicles (3-6 mm in diameter) from cows with moderated body energy reserve (MBER). | | |
| --- | --- | --- |
| Pathway | %^1^ | BH^2^ |
| bta04144 Endocytosis | 28.57143 | 0.0 |
| bta05200 Pathways in cancer | 23.24723 | 0.0092 |
| bta05205 Proteoglycans in cancer | 28.78049 | 0.0092 |
| bta04010 MAPK signaling pathway | 25.68493 | 0.0138 |
| bta04014 Ras signaling pathway | 26.44628 | 0.0138 |
| bta04022 cGMP-PKG signaling pathway | 28.40237 | 0.0138 |
| bta04530 Tight junction | 28.24859 | 0.0138 |
| bta05202 Transcriptional misregulation in cancer | 27.74869 | 0.0138 |
| bta04150 mTOR signaling pathway | 28.66242 | 0.0153 |
| bta01522 Endocrine resistance | 32.97872 | 0.0165 |
| bta04310 Wnt signaling pathway | 27.16049 | 0.0275 |
| bta04360 Axon guidance | 26.96629 | 0.0275 |
| bta04520 Adherens junction | 34.28571 | 0.0275 |
| bta04921 Oxytocin signaling pathway | 27.63158 | 0.0275 |
| bta05224 Breast cancer | 28 | 0.0275 |
| bta05223 Non-small cell lung cancer | 34.32836 | 0.0309 |
| bta04670 Leukocyte transendothelial migration | 29.20354 | 0.034 |
| bta04066 HIF-1 signaling pathway | 29.09091 | 0.0382 |
| bta04371 Apelin signaling pathway | 27.14286 | 0.04 |
| bta04660 T cell receptor signaling pathway | 28.97196 | 0.04 |
| bta05215 Prostate cancer | 29.59184 | 0.04 |
| bta05225 Hepatocellular carcinoma | 25.86207 | 0.04 |
| bta04390 Hippo signaling pathway | 26.28205 | 0.0401 |
| bta05412 Arrhythmogenic right ventricular cardiomyopathy (ARVC) | 31.57895 | 0.0401 |
| bta05226 Gastric cancer | 26.14379 | 0.0451 |
| bta04710 Circadian rhythm | 41.93548 | 0.0476 |
| bta04068 FoxO signaling pathway | 26.71756 | 0.0511 |
| bta04810 Regulation of actin cytoskeleton | 24.17062 | 0.0511 |
| bta04510 Focal adhesion | 24.24242 | 0.0588 |
| bta04380 Osteoclast differentiation | 26.1194 | 0.0632 |
| bta05220 Chronic myeloid leukemia | 29.87013 | 0.0639 |
| bta04012 ErbB signaling pathway | 28.57143 | 0.0778 |
| bta04514 Cell adhesion molecules (CAMs) | 24.68354 | 0.0778 |
| bta04550 Signaling pathways regulating pluripotency of stem cells | 25.35211 | 0.0778 |
| bta04919 Thyroid hormone signaling pathway | 26.27119 | 0.0778 |
| bta00564 Glycerophospholipid metabolism | 26.92308 | 0.078 |
| bta01521 EGFR tyrosine kinase inhibitor resistance | 28.75 | 0.078 |
| bta04211 Longevity regulating pathway | 27.77778 | 0.0796 |
| bta04370 VEGF signaling pathway | 31.03448 | 0.0839 |
| bta04934 Cushing syndrome | 24.35897 | 0.0866 |
| bta04072 Phospholipase D signaling pathway | 24.34211 | 0.0875 |
| bta04960 Aldosterone-regulated sodium reabsorption | 35.13514 | 0.0875 |
| bta05213 Endometrial cancer | 30.50847 | 0.0875 |
| bta05218 Melanoma | 28.76712 | 0.0875 |
| bta04152 AMPK signaling pathway | 25.20325 | 0.0947 |
| bta05210 Colorectal cancer | 26.96629 | 0.0986 |
| bta00310 Lysine degradation | 28.78788 | 0.1023 |
| bta04910 Insulin signaling pathway | 24.28571 | 0.1023 |
| bta04931 Insulin resistance | 25.45455 | 0.1023 |
| bta05230 Central carbon metabolism in cancer | 28.78788 | 0.1023 |
| bta04213 Longevity regulating pathway | 29.03226 | 0.1073 |
| bta04925 Aldosterone synthesis and secretion | 26.04167 | 0.1074 |
| bta04015 Rap1 signaling pathway | 22.22222 | 0.11 |
| bta05214 Glioma | 27.27273 | 0.11 |
| bta05217 Basal cell carcinoma | 28.57143 | 0.11 |
| bta05410 Hypertrophic cardiomyopathy (HCM) | 26.08696 | 0.11 |
| bta05221 Acute myeloid leukemia | 27.94118 | 0.1114 |
| bta04666 Fc gamma R-mediated phagocytosis | 25.80645 | 0.1157 |
| bta04978 Mineral absorption | 29.09091 | 0.1208 |
| bta05231 Choline metabolism in cancer | 25.25253 | 0.1208 |
| bta05414 Dilated cardiomyopathy (DCM) | 25.25253 | 0.1208 |
| bta04340 Hedgehog signaling pathway | 29.41176 | 0.1283 |
| bta04911 Insulin secretion | 25.88235 | 0.1283 |
| bta04130 SNARE interactions in vesicular transport | 33.33333 | 0.1289 |
| bta01100 Metabolic pathways | 17.94368 | 0.1338 |
| bta05212 Pancreatic cancer | 26.31579 | 0.1338 |
| bta04151 PI3K-Akt signaling pathway | 20.10724 | 0.1383 |
| bta00410 beta-Alanine metabolism | 32.35294 | 0.1411 |
| bta04662 B cell receptor signaling pathway | 25.28736 | 0.1411 |
| bta04261 Adrenergic signaling in cardiomyocytes | 22.66667 | 0.1477 |
| bta05235 PD-L1 expression and PD-1 checkpoint pathway in cancer | 24.73118 | 0.1491 |
| bta04070 Phosphatidylinositol signaling system | 24.24242 | 0.1585 |
| bta00512 Mucin type O-glycan biosynthesis | 32.25806 | 0.1632 |
| bta00514 Other types of O-glycan biosynthesis | 28.88889 | 0.1632 |
| bta00533 Glycosaminoglycan biosynthesis | 42.85714 | 0.1632 |
| bta04611 Platelet activation | 23.1405 | 0.1646 |
| bta04664 Fc epsilon RI signaling pathway | 25.71429 | 0.1693 |
| bta03430 Mismatch repair | 34.78261 | 0.1751 |
| bta04930 Type II diabetes mellitus | 28.26087 | 0.1751 |
| bta00770 Pantothenate and CoA biosynthesis | 36.84211 | 0.1759 |
| bta04218 Cellular senescence | 21.68675 | 0.1759 |
| bta04916 Melanogenesis | 23.52941 | 0.1759 |
| bta05211 Renal cell carcinoma | 25.35211 | 0.1759 |
| bta04120 Ubiquitin mediated proteolysis | 22.14286 | 0.1837 |
| bta00561 Glycerolipid metabolism | 25.37313 | 0.186 |
| bta04024 cAMP signaling pathway | 20.52402 | 0.1897 |
| bta04914 Progesterone-mediated oocyte maturation | 23.86364 | 0.1897 |
| bta00562 Inositol phosphate metabolism | 24.65753 | 0.191 |
| bta04020 Calcium signaling pathway | 20.79208 | 0.191 |
| bta05100 Bacterial invasion of epithelial cells | 24.65753 | 0.191 |
| bta04140 Autophagy | 21.83099 | 0.1922 |
| bta01040 Biosynthesis of unsaturated fatty acids | 30 | 0.2191 |
| bta05166 Human T-cell leukemia virus 1 infection | 20.08547 | 0.2253 |
| bta04668 TNF signaling pathway | 22.0339 | 0.2261 |
| bta04961 Endocrine and other factor-regulated calcium reabsorption | 26 | 0.2261 |
| bta04114 Oocyte meiosis | 21.84874 | 0.2276 |
| bta04137 Mitophagy | 24.24242 | 0.2276 |
| bta04750 Inflammatory mediator regulation of TRP channels | 22.3301 | 0.2276 |
| bta04926 Relaxin signaling pathway | 21.53846 | 0.2276 |
| bta04971 Gastric acid secretion | 23.68421 | 0.2276 |
| bta05135 Yersinia infection | 21.53846 | 0.2276 |
| bta00790 Folate biosynthesis | 27.77778 | 0.2279 |
| bta03030 DNA replication | 27.77778 | 0.2279 |
| bta04350 TGF-beta signaling pathway | 22.58065 | 0.2279 |
| bta04924 Renin secretion | 23.61111 | 0.2279 |
| bta04928 Parathyroid hormone synthesis. secretion and action | 22.11538 | 0.2279 |
| bta04964 Proximal tubule bicarbonate reclamation | 31.81818 | 0.2279 |
| bta05160 Hepatitis C | 20.73171 | 0.2279 |
| bta04976 Bile secretion | 22.89157 | 0.2331 |
| bta04062 Chemokine signaling pathway | 20.21277 | 0.2335 |
| bta04142 Lysosome | 21.21212 | 0.2335 |
| bta05020 Prion diseases | 28.125 | 0.2335 |
| bta05219 Bladder cancer | 26.19048 | 0.2344 |
| bta04141 Protein processing in endoplasmic reticulum | 20.48193 | 0.2347 |
| bta04270 Vascular smooth muscle contraction | 21.05263 | 0.2356 |
| bta04722 Neurotrophin signaling pathway | 21.31148 | 0.2356 |
| bta00360 Phenylalanine metabolism | 30.43478 | 0.2373 |
| bta00515 Mannose type O-glycan biosynthesis | 30.43478 | 0.2373 |
| bta04923 Regulation of lipolysis in adipocytes | 24.13793 | 0.2373 |
| bta05165 Human papillomavirus infection | 18.84058 | 0.2379 |
| bta04935 Growth hormone synthesis. secretion and action | 21.18644 | 0.2473 |
| bta04659 Th17 cell differentiation | 21.23894 | 0.2509 |
| bta04972 Pancreatic secretion | 21.56863 | 0.2509 |
| bta04933 AGE-RAGE signaling pathway in diabetic complications | 21.35922 | 0.2627 |
| bta05032 Morphine addiction | 21.73913 | 0.2627 |
| bta04071 Sphingolipid signaling pathway | 20.83333 | 0.2669 |
| bta00532 Glycosaminoglycan biosynthesis | 30 | 0.2761 |
| bta03420 Nucleotide excision repair | 24.44444 | 0.2761 |
| bta04216 Ferroptosis | 24.44444 | 0.2761 |
| bta05167 Kaposi sarcoma-associated herpesvirus infection | 19.41748 | 0.2761 |
| bta00230 Purine metabolism | 20.14925 | 0.2983 |
| bta00510 N-Glycan biosynthesis | 23.07692 | 0.3146 |
| bta00250 Alanine. aspartate and glutamate metabolism | 24.32432 | 0.3347 |
| bta04725 Cholinergic synapse | 20.17544 | 0.3347 |
| bta04658 Th1 and Th2 cell differentiation | 20.40816 | 0.3502 |
| bta00620 Pyruvate metabolism | 23.68421 | 0.3537 |
| bta04912 GnRH signaling pathway | 20.43011 | 0.3537 |
| bta04927 Cortisol synthesis and secretion | 21.53846 | 0.3537 |
| bta04929 GnRH secretion | 21.53846 | 0.3537 |
| bta00600 Sphingolipid metabolism | 22.44898 | 0.3581 |
| bta03410 Base excision repair | 24.24242 | 0.3581 |
| bta00340 Histidine metabolism | 26.08696 | 0.3703 |
| bta05162 Measles | 19.07895 | 0.3713 |
| bta01524 Platinum drug resistance | 20.51282 | 0.3808 |
| bta04146 Peroxisome | 20.2381 | 0.3858 |
| bta01230 Biosynthesis of amino acids | 20.54795 | 0.3905 |
| bta00220 Arginine biosynthesis | 26.31579 | 0.4044 |
| bta04727 GABAergic synapse | 19.78022 | 0.4044 |
| bta04913 Ovarian steroidogenesis | 21.05263 | 0.4044 |
| bta04720 Long-term potentiation | 20.28986 | 0.4202 |
| bta05418 Fluid shear stress and atherosclerosis | 18.62069 | 0.4278 |
| bta00010 Glycolysis Gluconeogenesis | 20.3125 | 0.4324 |
| bta04110 Cell cycle | 18.69919 | 0.4504 |
| bta04975 Fat digestion and absorption | 20.83333 | 0.4542 |
| bta05161 Hepatitis B | 18.12865 | 0.4542 |
| bta04210 Apoptosis | 18.30986 | 0.4612 |
| bta04512 ECM-receptor interaction | 19.10112 | 0.4612 |
| bta04917 Prolactin signaling pathway | 19.27711 | 0.4612 |
| bta00513 Various types of N-glycan biosynthesis | 20.93023 | 0.4618 |
| bta05163 Human cytomegalovirus infection | 17.55102 | 0.4666 |
| bta04721 Synaptic vesicle cycle | 19.23077 | 0.4685 |
| bta04540 Gap junction | 18.88889 | 0.47 |
| bta05017 Spinocerebellar ataxia | 18.75 | 0.47 |
| bta05170 Human immunodeficiency virus 1 infection | 17.52137 | 0.47 |
| bta04915 Estrogen signaling pathway | 18.11594 | 0.4735 |
| bta04973 Carbohydrate digestion and absorption | 20.45455 | 0.474 |
| bta03018 RNA degradation | 18.98734 | 0.4751 |
| bta00240 Pyrimidine metabolism | 19.64286 | 0.4822 |
| bta00350 Tyrosine metabolism | 20.51282 | 0.4891 |
| bta04215 Apoptosis | 20.58824 | 0.5123 |
| bta00062 Fatty acid elongation | 20.68966 | 0.5144 |
| bta00565 Ether lipid metabolism | 19.23077 | 0.5144 |
| bta00592 alpha-Linolenic acid metabolism | 20.68966 | 0.5144 |
| bta00601 Glycosphingolipid biosynthesis | 20.68966 | 0.5144 |
| bta01212 Fatty acid metabolism | 18.96552 | 0.5144 |
| bta04625 C-type lectin receptor signaling pathway | 17.92453 | 0.5144 |
| bta05169 Epstein-Barr virus infection | 17.10526 | 0.5144 |
| bta05216 Thyroid cancer | 20 | 0.5144 |
| bta05222 Small cell lung cancer | 18.08511 | 0.5144 |
| bta01200 Carbon metabolism | 17.69912 | 0.5211 |
| bta03015 mRNA surveillance pathway | 17.89474 | 0.5211 |
| bta04260 Cardiac muscle contraction | 17.97753 | 0.5211 |
| bta04330 Notch signaling pathway | 18.86792 | 0.5211 |
| bta05206 MicroRNAs in cancer | 16.78082 | 0.5265 |
| bta00630 Glyoxylate and dicarboxylate metabolism | 20 | 0.5354 |
| bta04064 NF-kappa B signaling pathway | 17.43119 | 0.546 |
| bta05142 Chagas disease (American trypanosomiasis) | 17.3913 | 0.546 |
| bta04970 Salivary secretion | 17.2043 | 0.5933 |
| bta00563 Glycosylphosphatidylinositol (GPI)-anchor biosynthesis | 19.23077 | 0.5999 |
| bta04650 Natural killer cell mediated cytotoxicity | 16.79389 | 0.6024 |
| bta00983 Drug metabolism | 17.10526 | 0.6195 |
| bta04061 Viral protein interaction with cytokine and cytokine receptor | 16.84211 | 0.6221 |
| bta04136 Autophagy | 18.18182 | 0.6221 |
| bta04744 Phototransduction | 18.51852 | 0.6244 |
| bta04115 p53 signaling pathway | 16.88312 | 0.6302 |
| bta04728 Dopaminergic synapse | 16.41791 | 0.6367 |
| bta00051 Fructose and mannose metabolism | 17.64706 | 0.6414 |
| bta04922 Glucagon signaling pathway | 16.50485 | 0.6414 |
| bta00380 Tryptophan metabolism | 17.02128 | 0.643 |
| bta04640 Hematopoietic cell lineage | 16.36364 | 0.643 |
| bta04920 Adipocytokine signaling pathway | 16.66667 | 0.643 |
| bta04966 Collecting duct acid secretion | 17.85714 | 0.643 |
| bta00071 Fatty acid degradation | 16.66667 | 0.6665 |
| bta03020 RNA polymerase | 17.24138 | 0.6665 |
| bta04060 Cytokine-cytokine receptor interaction | 15.78947 | 0.6665 |
| bta04145 Phagosome | 15.88235 | 0.6665 |
| bta04392 Hippo signaling pathway | 17.24138 | 0.6665 |
| bta04918 Thyroid hormone synthesis | 16.21622 | 0.6665 |
| bta04713 Circadian entrainment | 16 | 0.6696 |
| bta00270 Cysteine and methionine metabolism | 16.32653 | 0.6736 |
| bta00260 Glycine. serine and threonine metabolism | 16.27907 | 0.6829 |
| bta05132 Salmonella infection | 15.625 | 0.6898 |
| bta03022 Basal transcription factors | 15.90909 | 0.7015 |
| bta05321 Inflammatory bowel disease (IBD) | 15.71429 | 0.7015 |
| bta05146 Amoebiasis | 15.38462 | 0.7232 |
| bta00760 Nicotinate and nicotinamide metabolism | 15.38462 | 0.7378 |
| bta04657 IL-17 signaling pathway | 15.21739 | 0.7378 |
| bta00640 Propanoate metabolism | 15.15152 | 0.7552 |
| bta00140 Steroid hormone biosynthesis | 14.92537 | 0.7611 |
| bta04217 Necroptosis | 14.94253 | 0.7726 |
| bta00330 Arginine and proline metabolism | 14.58333 | 0.7826 |
| bta04630 JAK-STAT signaling pathway | 14.85149 | 0.7852 |
| bta05031 Amphetamine addiction | 14.49275 | 0.7885 |
| bta04962 Vasopressin-regulated water reabsorption | 14.28571 | 0.7958 |
| bta03013 RNA transport | 14.60674 | 0.8021 |
| bta04932 Non-alcoholic fatty liver disease (NAFLD) | 14.55696 | 0.8021 |
| bta00280 Valine. leucine and isoleucine degradation | 13.72549 | 0.8262 |
| bta04724 Glutamatergic synapse | 14.15929 | 0.8262 |
| bta00980 Metabolism of xenobiotics by cytochrome P450 | 13.43284 | 0.85 |
| bta03320 PPAR signaling pathway | 13.58025 | 0.85 |
| bta04730 Long-term depression | 13.33333 | 0.85 |
| bta05014 Amyotrophic lateral sclerosis (ALS) | 13.33333 | 0.85 |
| bta04620 Toll-like receptor signaling pathway | 13.63636 | 0.8606 |
| bta00860 Porphyrin and chlorophyll metabolism | 12.5 | 0.8772 |
| bta03040 Spliceosome | 13.60544 | 0.8812 |
| bta00982 Drug metabolism | 12.69841 | 0.8839 |
| bta05145 Toxoplasmosis | 13.27434 | 0.8839 |
| bta03440 Homologous recombination | 11.90476 | 0.9016 |
| bta05010 Alzheimer disease | 13.33333 | 0.9123 |
| bta05134 Legionellosis | 12.06897 | 0.9123 |
| bta00970 Aminoacyl-tRNA biosynthesis | 12.12121 | 0.9131 |
| bta00480 Glutathione metabolism | 11.66667 | 0.9263 |
| bta04714 Thermogenesis | 13.38912 | 0.9263 |
| bta04974 Protein digestion and absorption | 12.39669 | 0.9412 |
| bta00830 Retinol metabolism | 10.9375 | 0.967 |
| bta05168 Herpes simplex virus 1 infection | 13.3995 | 0.9687 |
| bta05203 Viral carcinogenesis | 12.86307 | 0.9687 |
| bta05323 Rheumatoid arthritis | 11.53846 | 0.9698 |
| bta04623 Cytosolic DNA-sensing pathway | 10.44776 | 0.9807 |
| bta04726 Serotonergic synapse | 11.2069 | 0.9851 |
| bta05152 Tuberculosis | 12.12121 | 0.9851 |
| bta05204 Chemical carcinogenesis | 10.38961 | 0.9851 |
| bta05140 Leishmaniasis | 10.25641 | 0.9884 |
| bta03008 Ribosome biogenesis in eukaryotes | 9.638554 | 0.9942 |
| bta04610 Complement and coagulation cascades | 9.782609 | 0.9942 |
| bta04621 NOD-like receptor signaling pathway | 11.41304 | 0.9942 |
| bta04622 RIG-I-like receptor signaling pathway | 9.803922 | 0.9942 |
| bta04672 Intestinal immune network for IgA production | 8.928571 | 0.9942 |
| bta04940 Type I diabetes mellitus | 8.474576 | 0.9942 |
| bta05012 Parkinson disease | 11.33333 | 0.9942 |
| bta05144 Malaria | 8.474576 | 0.9942 |
| bta05164 Influenza A | 11.60221 | 0.9942 |
| bta04723 Retrograde endocannabinoid signaling | 10.52632 | 0.995 |
| bta04742 Taste transduction | 8.860759 | 0.995 |
| bta00190 Oxidative phosphorylation | 7.857143 | 1.0 |
| bta00590 Arachidonic acid metabolism | 7.317073 | 1.0 |
| bta03010 Ribosome | 4.375 | 1.0 |
| bta04080 Neuroactive ligand-receptor interaction | 11.29477 | 1.0 |
| bta04612 Antigen processing and presentation | 5.882353 | 1.0 |
| bta04740 Olfactory transduction | 2.09607 | 1.0 |
| bta05016 Huntington disease | 10.94891 | 1.0 |
| bta05034 Alcoholism | 10.04367 | 1.0 |
| bta05133 Pertussis | 6.493506 | 1.0 |
| bta05150 Staphylococcus aureus infection | 6.666667 | 1.0 |
| bta05322 Systemic lupus erythematosus | 3.296703 | 1.0 |
| \| ^1^%: Percent of genes predicted to be modulated. ^2^BH: Benjamini – Hochberg \| \| --- \| | | |
