# Supplementary material for: High body energy reserve influences extracellular vesicles miRNA contents within the ovarian follicle
Source: PLoS One. 2023 Jan 10;18(1):e0280195. doi: 10.1371/journal.pone.0280195 (PMC9831338; doi:10.1371/journal.pone.0280195)
Supplement: S10 Table — (DOCX) [file pone.0280195.s013.docx]

| **Supplementary table 10.** Biological patwhays predicted as modulated by miRNAs up regulated in cumulus cells (CC) compared to follicular fluid extracellular vesicles (EV FF) from ipsi and contralateral ovarian follicles (3-6 mm in diameter) from cows with moderated body energy reserve (MBER). | | |
| --- | --- | --- |
| **Pathway** | **%^1^** | **BH^2^** |
| bta04360 Axon guidance | 21.91011 | 0.0 |
| bta04520 Adherens junction | 30 | 0.0 |
| bta05200 Pathways in cancer | 16.23616 | 0.0 |
| bta04722 Neurotrophin signaling pathway | 21.31148 | 0.028 |
| bta04014 Ras signaling pathway | 16.52893 | 0.0576 |
| bta04022 cGMP-PKG signaling pathway | 18.3432 | 0.0576 |
| bta04924 Renin secretion | 23.61111 | 0.0576 |
| bta04921 Oxytocin signaling pathway | 18.42105 | 0.0582 |
| bta04931 Insulin resistance | 20 | 0.0582 |
| bta05031 Amphetamine addiction | 23.18841 | 0.0582 |
| bta04150 mTOR signaling pathway | 17.83439 | 0.0631 |
| bta04010 MAPK signaling pathway | 14.72603 | 0.0784 |
| bta04020 Calcium signaling pathway | 15.84158 | 0.0784 |
| bta04024 cAMP signaling pathway | 15.72052 | 0.0784 |
| bta04130 SNARE interactions in vesicular transport | 27.27273 | 0.0784 |
| bta04216 Ferroptosis | 24.44444 | 0.0784 |
| bta04261 Adrenergic signaling in cardiomyocytes | 17.33333 | 0.0784 |
| bta04270 Vascular smooth muscle contraction | 18.04511 | 0.0784 |
| bta04713 Circadian entrainment | 19 | 0.0784 |
| bta04728 Dopaminergic synapse | 17.91045 | 0.0784 |
| bta04744 Phototransduction | 29.62963 | 0.0784 |
| bta04910 Insulin signaling pathway | 17.14286 | 0.0784 |
| bta04916 Melanogenesis | 18.62745 | 0.0784 |
| bta04925 Aldosterone synthesis and secretion | 19.79167 | 0.0784 |
| bta04934 Cushing syndrome | 16.66667 | 0.0784 |
| bta04961 Endocrine and other factor-regulated calcium reabsorption | 24 | 0.0784 |
| bta04510 Focal adhesion | 15.65657 | 0.0838 |
| bta00270 Cysteine and methionine metabolism | 22.44898 | 0.099 |
| bta04310 Wnt signaling pathway | 16.04938 | 0.099 |
| bta05032 Morphine addiction | 18.47826 | 0.099 |
| bta05214 Glioma | 19.48052 | 0.099 |
| bta04142 Lysosome | 16.66667 | 0.1018 |
| bta05211 Renal cell carcinoma | 19.71831 | 0.1018 |
| bta05152 Tuberculosis | 15.15152 | 0.1061 |
| bta05224 Breast cancer | 16 | 0.1101 |
| bta04928 Parathyroid hormone synthesis. secretion and action | 17.30769 | 0.1188 |
| bta04810 Regulation of actin cytoskeleton | 14.69194 | 0.1189 |
| bta05163 Human cytomegalovirus infection | 14.28571 | 0.1189 |
| bta05226 Gastric cancer | 15.68627 | 0.1189 |
| bta05215 Prostate cancer | 17.34694 | 0.1226 |
| bta04012 ErbB signaling pathway | 17.85714 | 0.1263 |
| bta04720 Long-term potentiation | 18.84058 | 0.1263 |
| bta04971 Gastric acid secretion | 18.42105 | 0.1263 |
| bta05212 Pancreatic cancer | 18.42105 | 0.1263 |
| bta04151 PI3K-Akt signaling pathway | 13.13673 | 0.1292 |
| bta04970 Salivary secretion | 17.2043 | 0.1292 |
| bta04978 Mineral absorption | 20 | 0.1292 |
| bta01522 Endocrine resistance | 17.02128 | 0.1363 |
| bta05100 Bacterial invasion of epithelial cells | 17.80822 | 0.1605 |
| bta04340 Hedgehog signaling pathway | 19.60784 | 0.1634 |
| bta05167 Kaposi sarcoma-associated herpesvirus infection | 14.07767 | 0.1634 |
| bta04625 C-type lectin receptor signaling pathway | 16.03774 | 0.1667 |
| bta04015 Rap1 signaling pathway | 13.88889 | 0.1668 |
| bta04371 Apelin signaling pathway | 15 | 0.1668 |
| bta05231 Choline metabolism in cancer | 16.16162 | 0.1668 |
| bta05414 Dilated cardiomyopathy (DCM) | 16.16162 | 0.1668 |
| bta05412 Arrhythmogenic right ventricular cardiomyopathy (ARVC) | 17.10526 | 0.1749 |
| bta04911 Insulin secretion | 16.47059 | 0.183 |
| bta04912 GnRH signaling pathway | 16.12903 | 0.183 |
| bta04072 Phospholipase D signaling pathway | 14.47368 | 0.1836 |
| bta04919 Thyroid hormone signaling pathway | 15.25424 | 0.1836 |
| bta04114 Oocyte meiosis | 15.12605 | 0.1874 |
| bta05205 Proteoglycans in cancer | 13.65854 | 0.1874 |
| bta05217 Basal cell carcinoma | 17.46032 | 0.1925 |
| bta00532 Glycosaminoglycan biosynthesis | 25 | 0.2006 |
| bta04915 Estrogen signaling pathway | 14.49275 | 0.2006 |
| bta04920 Adipocytokine signaling pathway | 16.66667 | 0.2006 |
| bta04926 Relaxin signaling pathway | 14.61538 | 0.2006 |
| bta05210 Colorectal cancer | 15.73034 | 0.2055 |
| bta04923 Regulation of lipolysis in adipocytes | 17.24138 | 0.2214 |
| bta00230 Purine metabolism | 14.1791 | 0.2327 |
| bta05223 Non-small cell lung cancer | 16.41791 | 0.2327 |
| bta00514 Other types of O-glycan biosynthesis | 17.77778 | 0.2513 |
| bta04972 Pancreatic secretion | 14.70588 | 0.2513 |
| bta05220 Chronic myeloid leukemia | 15.58442 | 0.2513 |
| bta05222 Small cell lung cancer | 14.89362 | 0.2513 |
| bta05418 Fluid shear stress and atherosclerosis | 13.7931 | 0.2513 |
| bta04071 Sphingolipid signaling pathway | 14.16667 | 0.2519 |
| bta04721 Synaptic vesicle cycle | 15.38462 | 0.2566 |
| bta05135 Yersinia infection | 13.84615 | 0.2618 |
| bta04218 Cellular senescence | 13.25301 | 0.2628 |
| bta04659 Th17 cell differentiation | 14.15929 | 0.2628 |
| bta04710 Circadian rhythm | 19.35484 | 0.2642 |
| bta01230 Biosynthesis of amino acids | 15.06849 | 0.2802 |
| bta04062 Chemokine signaling pathway | 12.76596 | 0.2802 |
| bta04140 Autophagy | 13.38028 | 0.2802 |
| bta04658 Th1 and Th2 cell differentiation | 14.28571 | 0.2802 |
| bta04660 T cell receptor signaling pathway | 14.01869 | 0.2802 |
| bta05216 Thyroid cancer | 17.5 | 0.2802 |
| bta05218 Melanoma | 15.06849 | 0.2802 |
| bta04913 Ovarian steroidogenesis | 15.78947 | 0.289 |
| bta04144 Endocytosis | 12.2449 | 0.2922 |
| bta04657 IL-17 signaling pathway | 14.13043 | 0.2964 |
| bta05410 Hypertrophic cardiomyopathy (HCM) | 14.13043 | 0.2964 |
| bta04977 Vitamin digestion and absorption | 19.23077 | 0.298 |
| bta05160 Hepatitis C | 12.80488 | 0.298 |
| bta05225 Hepatocellular carcinoma | 12.64368 | 0.3048 |
| bta05213 Endometrial cancer | 15.25424 | 0.3072 |
| bta04750 Inflammatory mediator regulation of TRP channels | 13.59223 | 0.3116 |
| bta04922 Glucagon signaling pathway | 13.59223 | 0.3116 |
| bta05014 Amyotrophic lateral sclerosis (ALS) | 15 | 0.3178 |
| bta04110 Cell cycle | 13.00813 | 0.3325 |
| bta04725 Cholinergic synapse | 13.15789 | 0.3325 |
| bta05321 Inflammatory bowel disease (IBD) | 14.28571 | 0.3354 |
| bta01521 EGFR tyrosine kinase inhibitor resistance | 13.75 | 0.3514 |
| bta05161 Hepatitis B | 12.2807 | 0.3514 |
| bta05202 Transcriptional misregulation in cancer | 12.04188 | 0.3611 |
| bta04727 GABAergic synapse | 13.18681 | 0.3812 |
| bta04390 Hippo signaling pathway | 12.17949 | 0.3851 |
| bta00350 Tyrosine metabolism | 15.38462 | 0.3868 |
| bta04927 Cortisol synthesis and secretion | 13.84615 | 0.3868 |
| bta04976 Bile secretion | 13.25301 | 0.3868 |
| bta05030 Cocaine addiction | 14.58333 | 0.39 |
| bta05166 Human T-cell leukemia virus 1 infection | 11.53846 | 0.39 |
| bta05170 Human immunodeficiency virus 1 infection | 11.53846 | 0.39 |
| bta04611 Platelet activation | 12.39669 | 0.3937 |
| bta04962 Vasopressin-regulated water reabsorption | 14.28571 | 0.4043 |
| bta04080 Neuroactive ligand-receptor interaction | 11.01928 | 0.4081 |
| bta05165 Human papillomavirus infection | 11.01449 | 0.4183 |
| bta00480 Glutathione metabolism | 13.33333 | 0.4422 |
| bta03018 RNA degradation | 12.65823 | 0.4521 |
| bta04211 Longevity regulating pathway | 12.22222 | 0.4803 |
| bta04330 Notch signaling pathway | 13.20755 | 0.481 |
| bta04514 Cell adhesion molecules (CAMs) | 11.39241 | 0.4885 |
| bta03013 RNA transport | 11.23596 | 0.4923 |
| bta04120 Ubiquitin mediated proteolysis | 11.42857 | 0.5008 |
| bta04917 Prolactin signaling pathway | 12.04819 | 0.5085 |
| bta04350 TGF-beta signaling pathway | 11.82796 | 0.5093 |
| bta05235 PD-L1 expression and PD-1 checkpoint pathway in cancer | 11.82796 | 0.5093 |
| bta01100 Metabolic pathways | 10.01965 | 0.51 |
| bta00250 Alanine. aspartate and glutamate metabolism | 13.51351 | 0.5103 |
| bta04724 Glutamatergic synapse | 11.50442 | 0.5103 |
| bta04152 AMPK signaling pathway | 11.38211 | 0.511 |
| bta03015 mRNA surveillance pathway | 11.57895 | 0.5264 |
| bta00140 Steroid hormone biosynthesis | 11.9403 | 0.5394 |
| bta04975 Fat digestion and absorption | 12.5 | 0.5394 |
| bta05133 Pertussis | 11.68831 | 0.5409 |
| bta00760 Nicotinate and nicotinamide metabolism | 12.82051 | 0.5434 |
| bta00600 Sphingolipid metabolism | 12.2449 | 0.5467 |
| bta04530 Tight junction | 10.73446 | 0.5467 |
| bta05221 Acute myeloid leukemia | 11.76471 | 0.5467 |
| bta04668 TNF signaling pathway | 11.01695 | 0.5493 |
| bta05169 Epstein-Barr virus infection | 10.52632 | 0.5493 |
| bta04070 Phosphatidylinositol signaling system | 11.11111 | 0.5588 |
| bta04664 Fc epsilon RI signaling pathway | 11.42857 | 0.566 |
| bta04066 HIF-1 signaling pathway | 10.90909 | 0.5689 |
| bta00510 N-Glycan biosynthesis | 11.53846 | 0.5761 |
| bta04550 Signaling pathways regulating pluripotency of stem cells | 10.56338 | 0.5761 |
| bta04650 Natural killer cell mediated cytotoxicity | 10.68702 | 0.5761 |
| bta04723 Retrograde endocannabinoid signaling | 10.52632 | 0.5761 |
| bta05162 Measles | 10.52632 | 0.5761 |
| bta05219 Bladder cancer | 11.90476 | 0.5761 |
| bta04621 NOD-like receptor signaling pathway | 10.32609 | 0.5888 |
| bta04380 Osteoclast differentiation | 10.44776 | 0.5971 |
| bta00564 Glycerophospholipid metabolism | 10.57692 | 0.5997 |
| bta04146 Peroxisome | 10.71429 | 0.5997 |
| bta04918 Thyroid hormone synthesis | 10.81081 | 0.5997 |
| bta04726 Serotonergic synapse | 10.34483 | 0.6134 |
| bta05034 Alcoholism | 10.04367 | 0.6134 |
| bta00310 Lysine degradation | 10.60606 | 0.6307 |
| bta00561 Glycerolipid metabolism | 10.44776 | 0.635 |
| bta00980 Metabolism of xenobiotics by cytochrome P450 | 10.44776 | 0.635 |
| bta04115 p53 signaling pathway | 10.38961 | 0.635 |
| bta05204 Chemical carcinogenesis | 10.38961 | 0.635 |
| bta04914 Progesterone-mediated oocyte maturation | 10.22727 | 0.6367 |
| bta05132 Salmonella infection | 9.821429 | 0.6414 |
| bta04068 FoxO signaling pathway | 9.923664 | 0.6416 |
| bta04260 Cardiac muscle contraction | 10.11236 | 0.6416 |
| bta04512 ECM-receptor interaction | 10.11236 | 0.6416 |
| bta04217 Necroptosis | 9.770115 | 0.6478 |
| bta04540 Gap junction | 10 | 0.6478 |
| bta03320 PPAR signaling pathway | 9.876543 | 0.6607 |
| bta04670 Leukocyte transendothelial migration | 9.734513 | 0.6607 |
| bta05145 Toxoplasmosis | 9.734513 | 0.6607 |
| bta05206 MicroRNAs in cancer | 9.589041 | 0.6607 |
| bta04213 Longevity regulating pathway | 9.677419 | 0.6941 |
| bta04061 Viral protein interaction with cytokine and cytokine receptor | 9.473684 | 0.6975 |
| bta04630 JAK-STAT signaling pathway | 9.405941 | 0.6975 |
| bta00982 Drug metabolism | 9.52381 | 0.7005 |
| bta04612 Antigen processing and presentation | 9.411765 | 0.7005 |
| bta05017 Spinocerebellar ataxia | 9.375 | 0.7005 |
| bta04935 Growth hormone synthesis. secretion and action | 9.322034 | 0.7014 |
| bta00830 Retinol metabolism | 9.375 | 0.703 |
| bta04064 NF-kappa B signaling pathway | 9.174312 | 0.7103 |
| bta04662 B cell receptor signaling pathway | 9.195402 | 0.7103 |
| bta04929 GnRH secretion | 9.230769 | 0.7103 |
| bta04137 Mitophagy | 9.090909 | 0.7215 |
| bta01524 Platinum drug resistance | 8.974359 | 0.7312 |
| bta00240 Pyrimidine metabolism | 8.928571 | 0.734 |
| bta04742 Taste transduction | 8.860759 | 0.7381 |
| bta04622 RIG-I-like receptor signaling pathway | 8.823529 | 0.7427 |
| bta04933 AGE-RAGE signaling pathway in diabetic complications | 8.737864 | 0.7481 |
| bta05134 Legionellosis | 8.62069 | 0.7481 |
| bta05164 Influenza A | 8.839779 | 0.7481 |
| bta05142 Chagas disease (American trypanosomiasis) | 8.695652 | 0.749 |
| bta04666 Fc gamma R-mediated phagocytosis | 8.602151 | 0.7517 |
| bta03008 Ribosome biogenesis in eukaryotes | 8.433735 | 0.7639 |
| bta04730 Long-term depression | 8.333333 | 0.7639 |
| bta04210 Apoptosis | 8.450704 | 0.7795 |
| bta04141 Protein processing in endoplasmic reticulum | 8.433735 | 0.7875 |
| bta00983 Drug metabolism | 7.894737 | 0.8006 |
| bta05010 Alzheimer disease | 8.333333 | 0.8006 |
| bta05416 Viral myocarditis | 7.894737 | 0.8006 |
| bta05230 Central carbon metabolism in cancer | 7.575758 | 0.8214 |
| bta04620 Toll-like receptor signaling pathway | 7.272727 | 0.8745 |
| bta04640 Hematopoietic cell lineage | 7.272727 | 0.8745 |
| bta04932 Non-alcoholic fatty liver disease (NAFLD) | 7.594937 | 0.8745 |
| bta05203 Viral carcinogenesis | 7.883817 | 0.8745 |
| bta00562 Inositol phosphate metabolism | 6.849315 | 0.8755 |
| bta04060 Cytokine-cytokine receptor interaction | 7.430341 | 0.9563 |
| bta01200 Carbon metabolism | 6.19469 | 0.959 |
| bta04145 Phagosome | 6.470588 | 0.9654 |
| bta05146 Amoebiasis | 5.982906 | 0.9654 |
| bta05323 Rheumatoid arthritis | 5.769231 | 0.9654 |
| bta04610 Complement and coagulation cascades | 5.434783 | 0.9691 |
| bta05012 Parkinson disease | 6 | 0.9736 |
| bta05016 Huntington disease | 6.569343 | 0.983 |
| bta03040 Spliceosome | 5.442177 | 0.9879 |
| bta04974 Protein digestion and absorption | 4.958678 | 0.9879 |
| bta05168 Herpes simplex virus 1 infection | 6.699752 | 0.9879 |
| bta00190 Oxidative phosphorylation | 4.285714 | 1.0 |
| bta04714 Thermogenesis | 5.020921 | 1.0 |
| bta04740 Olfactory transduction | 1.834061 | 1.0 |
| bta05322 Systemic lupus erythematosus | 2.747253 | 1.0 |
| ^1^%: Percent of genes predicted to be modulated. ^2^BH: Benjamini – Hochberg | | |
