# Supplementary material for: High body energy reserve influences extracellular vesicles miRNA contents within the ovarian follicle
Source: PLoS One. 2023 Jan 10;18(1):e0280195. doi: 10.1371/journal.pone.0280195 (PMC9831338; doi:10.1371/journal.pone.0280195)
Supplement: S11 Table — (DOCX) [file pone.0280195.s014.docx]

| **Supplementary table 11.** Biological patwhays predicted as modulated by exclusives miRNAs detected in follicular fluid extracellular vesicles (EV FF) compared to cumulus cells (CC) from ipsi and contralateral ovarian follicles (3-6 mm in diameter) from cows with high body energy reserve (HBER). | | |
| --- | --- | --- |
| Pathway | %^1^ | BH^2^ |
| bta01100 Metabolic pathways | 62.93386 | 0.0 |
| bta04010 MAPK signaling pathway | 72.26027 | 0.0128 |
| bta04014 Ras signaling pathway | 73.96694 | 0.0128 |
| bta04144 Endocytosis | 75.91837 | 0.0128 |
| bta05200 Pathways in cancer | 66.42066 | 0.0128 |
| bta04360 Axon guidance | 75.2809 | 0.0367 |
| bta05205 Proteoglycans in cancer | 73.17073 | 0.0367 |
| bta04810 Regulation of actin cytoskeleton | 72.03791 | 0.0482 |
| bta04071 Sphingolipid signaling pathway | 78.33333 | 0.0606 |
| bta04910 Insulin signaling pathway | 75.71429 | 0.0642 |
| bta04015 Rap1 signaling pathway | 69.90741 | 0.0688 |
| bta04072 Phospholipase D signaling pathway | 73.68421 | 0.0688 |
| bta04150 mTOR signaling pathway | 73.24841 | 0.0688 |
| bta04310 Wnt signaling pathway | 72.83951 | 0.0688 |
| bta04142 Lysosome | 75 | 0.0728 |
| bta00564 Glycerophospholipid metabolism | 75.96154 | 0.1009 |
| bta04070 Phosphatidylinositol signaling system | 76.76768 | 0.1009 |
| bta04270 Vascular smooth muscle contraction | 72.93233 | 0.1009 |
| bta04722 Neurotrophin signaling pathway | 74.59016 | 0.1009 |
| bta04921 Oxytocin signaling pathway | 71.71053 | 0.1009 |
| bta05165 Human papillomavirus infection | 64.34783 | 0.1009 |
| bta04934 Cushing syndrome | 70.51282 | 0.1117 |
| bta05231 Choline metabolism in cancer | 75.75758 | 0.1117 |
| bta04012 ErbB signaling pathway | 77.38095 | 0.1161 |
| bta04611 Platelet activation | 72.72727 | 0.1161 |
| bta05212 Pancreatic cancer | 78.94737 | 0.1161 |
| bta04928 Parathyroid hormone synthesis. secretion and action | 74.03846 | 0.126 |
| bta05220 Chronic myeloid leukemia | 77.92208 | 0.1273 |
| bta04750 Inflammatory mediator regulation of TRP channels | 73.78641 | 0.1295 |
| bta00230 Purine metabolism | 69.40299 | 0.1302 |
| bta00562 Inositol phosphate metabolism | 75.34247 | 0.1302 |
| bta01522 Endocrine resistance | 72.34043 | 0.1302 |
| bta04022 cGMP-PKG signaling pathway | 66.86391 | 0.1302 |
| bta04062 Chemokine signaling pathway | 67.02128 | 0.1302 |
| bta04068 FoxO signaling pathway | 69.46565 | 0.1302 |
| bta04120 Ubiquitin mediated proteolysis | 70 | 0.1302 |
| bta04140 Autophagy | 68.30986 | 0.1302 |
| bta04261 Adrenergic signaling in cardiomyocytes | 68 | 0.1302 |
| bta04390 Hippo signaling pathway | 67.30769 | 0.1302 |
| bta04510 Focal adhesion | 65.65657 | 0.1302 |
| bta04514 Cell adhesion molecules (CAMs) | 68.35443 | 0.1302 |
| bta04659 Th17 cell differentiation | 71.68142 | 0.1302 |
| bta04666 Fc gamma R-mediated phagocytosis | 73.11828 | 0.1302 |
| bta04668 TNF signaling pathway | 70.33898 | 0.1302 |
| bta04728 Dopaminergic synapse | 68.65672 | 0.1302 |
| bta04730 Long-term depression | 78.33333 | 0.1302 |
| bta04912 GnRH signaling pathway | 73.11828 | 0.1302 |
| bta04916 Melanogenesis | 73.52941 | 0.1302 |
| bta04919 Thyroid hormone signaling pathway | 72.0339 | 0.1302 |
| bta05132 Salmonella infection | 64.73214 | 0.1302 |
| bta05163 Human cytomegalovirus infection | 64.4898 | 0.1302 |
| bta05211 Renal cell carcinoma | 77.46479 | 0.1302 |
| bta05214 Glioma | 75.32468 | 0.1302 |
| bta05225 Hepatocellular carcinoma | 67.24138 | 0.1302 |
| bta04520 Adherens junction | 75.71429 | 0.1325 |
| bta04371 Apelin signaling pathway | 67.85714 | 0.1352 |
| bta05418 Fluid shear stress and atherosclerosis | 67.58621 | 0.1352 |
| bta04550 Signaling pathways regulating pluripotency of stem cells | 67.60563 | 0.1355 |
| bta04931 Insulin resistance | 70 | 0.1355 |
| bta04660 T cell receptor signaling pathway | 70.09346 | 0.1386 |
| bta04141 Protein processing in endoplasmic reticulum | 65.66265 | 0.1469 |
| bta04658 Th1 and Th2 cell differentiation | 70.40816 | 0.1469 |
| bta04933 AGE-RAGE signaling pathway in diabetic complications | 69.90291 | 0.1469 |
| bta05100 Bacterial invasion of epithelial cells | 73.9726 | 0.1469 |
| bta05135 Yersinia infection | 67.69231 | 0.1469 |
| bta05215 Prostate cancer | 70.40816 | 0.1469 |
| bta04370 VEGF signaling pathway | 75.86207 | 0.161 |
| bta05210 Colorectal cancer | 70.78652 | 0.161 |
| bta05224 Breast cancer | 66 | 0.161 |
| bta01200 Carbon metabolism | 68.14159 | 0.1637 |
| bta04926 Relaxin signaling pathway | 66.92308 | 0.1637 |
| bta04721 Synaptic vesicle cycle | 71.79487 | 0.1645 |
| bta04066 HIF-1 signaling pathway | 68.18182 | 0.1667 |
| bta00330 Arginine and proline metabolism | 77.08333 | 0.1732 |
| bta04151 PI3K-Akt signaling pathway | 60.05362 | 0.1732 |
| bta04218 Cellular senescence | 64.45783 | 0.1732 |
| bta04625 C-type lectin receptor signaling pathway | 67.92453 | 0.1732 |
| bta04720 Long-term potentiation | 72.46377 | 0.1732 |
| bta04935 Growth hormone synthesis. secretion and action | 66.94915 | 0.1732 |
| bta05161 Hepatitis B | 64.32749 | 0.1732 |
| bta05166 Human T-cell leukemia virus 1 infection | 62.39316 | 0.1732 |
| bta04530 Tight junction | 63.84181 | 0.1733 |
| bta04670 Leukocyte transendothelial migration | 67.25664 | 0.1733 |
| bta04020 Calcium signaling pathway | 62.87129 | 0.1743 |
| bta04914 Progesterone-mediated oocyte maturation | 69.31818 | 0.1743 |
| bta05235 PD-L1 expression and PD-1 checkpoint pathway in cancer | 68.8172 | 0.1743 |
| bta05170 Human immunodeficiency virus 1 infection | 61.96581 | 0.176 |
| bta05223 Non-small cell lung cancer | 71.64179 | 0.1864 |
| bta04925 Aldosterone synthesis and secretion | 67.70833 | 0.1958 |
| bta05226 Gastric cancer | 64.05229 | 0.1958 |
| bta04137 Mitophagy | 71.21212 | 0.1964 |
| bta05230 Central carbon metabolism in cancer | 71.21212 | 0.1964 |
| bta00514 Other types of O-glycan biosynthesis | 75.55556 | 0.199 |
| bta04114 Oocyte meiosis | 65.54622 | 0.199 |
| bta05217 Basal cell carcinoma | 71.42857 | 0.199 |
| bta05221 Acute myeloid leukemia | 70.58824 | 0.199 |
| bta01521 EGFR tyrosine kinase inhibitor resistance | 68.75 | 0.2022 |
| bta04152 AMPK signaling pathway | 65.04065 | 0.2023 |
| bta04726 Serotonergic synapse | 65.51724 | 0.2023 |
| bta04024 cAMP signaling pathway | 61.13537 | 0.2063 |
| bta05167 Kaposi sarcoma-associated herpesvirus infection | 61.65049 | 0.2063 |
| bta04146 Peroxisome | 67.85714 | 0.2077 |
| bta00600 Sphingolipid metabolism | 73.46939 | 0.208 |
| bta04922 Glucagon signaling pathway | 66.01942 | 0.208 |
| bta04130 SNARE interactions in vesicular transport | 78.78788 | 0.2106 |
| bta04350 TGF-beta signaling pathway | 66.66667 | 0.2106 |
| bta04971 Gastric acid secretion | 68.42105 | 0.2106 |
| bta00240 Pyrimidine metabolism | 71.42857 | 0.2128 |
| bta04064 NF-kappa B signaling pathway | 65.13761 | 0.217 |
| bta04911 Insulin secretion | 67.05882 | 0.2177 |
| bta05032 Morphine addiction | 66.30435 | 0.2192 |
| bta04216 Ferroptosis | 73.33333 | 0.2241 |
| bta04961 Endocrine and other factor-regulated calcium reabsorption | 72 | 0.2241 |
| bta04924 Renin secretion | 68.05556 | 0.2272 |
| bta00410 beta-Alanine metabolism | 76.47059 | 0.2347 |
| bta04710 Circadian rhythm | 77.41935 | 0.2427 |
| bta00533 Glycosaminoglycan biosynthesis | 92.85714 | 0.2455 |
| bta00591 Linoleic acid metabolism | 75 | 0.2455 |
| bta05160 Hepatitis C | 61.58537 | 0.2455 |
| bta01212 Fatty acid metabolism | 68.96552 | 0.2549 |
| bta04330 Notch signaling pathway | 69.81132 | 0.2549 |
| bta04927 Cortisol synthesis and secretion | 67.69231 | 0.2579 |
| bta04110 Cell cycle | 62.60163 | 0.2714 |
| bta01524 Platinum drug resistance | 65.38462 | 0.2845 |
| bta00310 Lysine degradation | 66.66667 | 0.285 |
| bta01230 Biosynthesis of amino acids | 65.75342 | 0.2864 |
| bta00670 One carbon pool by folate | 83.33333 | 0.2914 |
| bta04664 Fc epsilon RI signaling pathway | 65.71429 | 0.297 |
| bta04727 GABAergic synapse | 63.73626 | 0.297 |
| bta05202 Transcriptional misregulation in cancer | 59.68586 | 0.297 |
| bta00340 Histidine metabolism | 78.26087 | 0.2972 |
| bta04920 Adipocytokine signaling pathway | 65.27778 | 0.2974 |
| bta00561 Glycerolipid metabolism | 65.67164 | 0.3011 |
| bta04061 Viral protein interaction with cytokine and cytokine receptor | 63.15789 | 0.3011 |
| bta04540 Gap junction | 63.33333 | 0.3059 |
| bta05412 Arrhythmogenic right ventricular cardiomyopathy (ARVC) | 64.47368 | 0.3059 |
| bta00062 Fatty acid elongation | 72.41379 | 0.3087 |
| bta00220 Arginine biosynthesis | 78.94737 | 0.3087 |
| bta00270 Cysteine and methionine metabolism | 67.34694 | 0.3087 |
| bta00510 N-Glycan biosynthesis | 67.30769 | 0.3087 |
| bta00565 Ether lipid metabolism | 67.30769 | 0.3087 |
| bta00592 alpha-Linolenic acid metabolism | 72.41379 | 0.3087 |
| bta00760 Nicotinate and nicotinamide metabolism | 69.23077 | 0.3087 |
| bta00770 Pantothenate and CoA biosynthesis | 78.94737 | 0.3087 |
| bta03015 mRNA surveillance pathway | 62.10526 | 0.3087 |
| bta04115 p53 signaling pathway | 63.63636 | 0.3087 |
| bta04215 Apoptosis | 70.58824 | 0.3087 |
| bta04724 Glutamatergic synapse | 61.06195 | 0.3087 |
| bta04744 Phototransduction | 74.07407 | 0.3087 |
| bta04915 Estrogen signaling pathway | 60.14493 | 0.3087 |
| bta04930 Type II diabetes mellitus | 67.3913 | 0.3087 |
| bta04970 Salivary secretion | 62.36559 | 0.3087 |
| bta05017 Spinocerebellar ataxia | 62.5 | 0.3087 |
| bta05020 Prion diseases | 71.875 | 0.3087 |
| bta05133 Pertussis | 63.63636 | 0.3087 |
| bta05145 Toxoplasmosis | 61.06195 | 0.3087 |
| bta05321 Inflammatory bowel disease (IBD) | 64.28571 | 0.3087 |
| bta04210 Apoptosis | 59.85915 | 0.3103 |
| bta05142 Chagas disease (American trypanosomiasis) | 60.86957 | 0.3103 |
| bta04917 Prolactin signaling pathway | 62.6506 | 0.311 |
| bta04657 IL-17 signaling pathway | 61.95652 | 0.3126 |
| bta00515 Mannose type O-glycan biosynthesis | 73.91304 | 0.3197 |
| bta03420 Nucleotide excision repair | 66.66667 | 0.3197 |
| bta04380 Osteoclast differentiation | 59.70149 | 0.3197 |
| bta04966 Collecting duct acid secretion | 71.42857 | 0.3197 |
| bta05213 Endometrial cancer | 64.40678 | 0.3197 |
| bta05218 Melanoma | 63.0137 | 0.3197 |
| bta04713 Circadian entrainment | 61 | 0.3235 |
| bta00020 Citrate cycle (TCA cycle) | 70 | 0.3301 |
| bta05219 Bladder cancer | 66.66667 | 0.3301 |
| bta00500 Starch and sucrose metabolism | 68.75 | 0.3361 |
| bta04340 Hedgehog signaling pathway | 64.70588 | 0.3361 |
| bta04923 Regulation of lipolysis in adipocytes | 63.7931 | 0.3361 |
| bta05164 Influenza A | 58.01105 | 0.3361 |
| bta05410 Hypertrophic cardiomyopathy (HCM) | 60.86957 | 0.3361 |
| bta05414 Dilated cardiomyopathy (DCM) | 60.60606 | 0.3361 |
| bta00480 Glutathione metabolism | 63.33333 | 0.3371 |
| bta05031 Amphetamine addiction | 62.31884 | 0.3382 |
| bta05222 Small cell lung cancer | 60.6383 | 0.3382 |
| bta04978 Mineral absorption | 63.63636 | 0.3424 |
| bta00010 Glycolysis Gluconeogenesis | 62.5 | 0.3441 |
| bta05169 Epstein-Barr virus infection | 57.01754 | 0.3441 |
| bta00513 Various types of N-glycan biosynthesis | 65.11628 | 0.3451 |
| bta03030 DNA replication | 66.66667 | 0.3451 |
| bta04913 Ovarian steroidogenesis | 63.15789 | 0.3451 |
| bta00512 Mucin type O-glycan biosynthesis | 67.74194 | 0.3505 |
| bta04136 Autophagy | 66.66667 | 0.3616 |
| bta05216 Thyroid cancer | 65 | 0.3616 |
| bta00071 Fatty acid degradation | 64.28571 | 0.3669 |
| bta04725 Cholinergic synapse | 58.77193 | 0.3669 |
| bta04962 Vasopressin-regulated water reabsorption | 63.26531 | 0.3669 |
| bta01040 Biosynthesis of unsaturated fatty acids | 66.66667 | 0.37 |
| bta03430 Mismatch repair | 69.56522 | 0.37 |
| bta04662 B cell receptor signaling pathway | 59.77011 | 0.37 |
| bta04960 Aldosterone-regulated sodium reabsorption | 64.86486 | 0.37 |
| bta05014 Amyotrophic lateral sclerosis (ALS) | 61.66667 | 0.37 |
| bta05152 Tuberculosis | 56.56566 | 0.3788 |
| bta00100 Steroid biosynthesis | 70 | 0.3822 |
| bta00532 Glycosaminoglycan biosynthesis | 70 | 0.3822 |
| bta05162 Measles | 57.23684 | 0.3822 |
| bta05340 Primary immunodeficiency | 63.41463 | 0.3822 |
| bta04640 Hematopoietic cell lineage | 58.18182 | 0.3855 |
| bta00260 Glycine. serine and threonine metabolism | 62.7907 | 0.3863 |
| bta04211 Longevity regulating pathway | 58.88889 | 0.3863 |
| bta03460 Fanconi anemia pathway | 61.53846 | 0.3868 |
| bta03020 RNA polymerase | 65.51724 | 0.39 |
| bta04918 Thyroid hormone synthesis | 59.45946 | 0.3936 |
| bta00620 Pyruvate metabolism | 63.15789 | 0.3943 |
| bta00380 Tryptophan metabolism | 61.70213 | 0.395 |
| bta00052 Galactose metabolism | 64.51613 | 0.3951 |
| bta00280 Valine. leucine and isoleucine degradation | 60.78431 | 0.4038 |
| bta00563 Glycosylphosphatidylinositol (GPI)-anchor biosynthesis | 65.38462 | 0.4038 |
| bta00640 Propanoate metabolism | 63.63636 | 0.4038 |
| bta04621 NOD-like receptor signaling pathway | 55.97826 | 0.4038 |
| bta00900 Terpenoid backbone biosynthesis | 66.66667 | 0.4174 |
| bta05030 Cocaine addiction | 60.41667 | 0.4238 |
| bta04976 Bile secretion | 57.83133 | 0.4291 |
| bta04512 ECM-receptor interaction | 57.30337 | 0.4399 |
| bta04972 Pancreatic secretion | 56.86275 | 0.4399 |
| bta05146 Amoebiasis | 56.41026 | 0.44 |
| bta05323 Rheumatoid arthritis | 56.73077 | 0.44 |
| bta00590 Arachidonic acid metabolism | 57.31707 | 0.4468 |
| bta00601 Glycosphingolipid biosynthesis | 62.06897 | 0.4501 |
| bta04217 Necroptosis | 55.17241 | 0.4501 |
| bta04392 Hippo signaling pathway | 62.06897 | 0.4501 |
| bta00531 Glycosaminoglycan degradation | 63.63636 | 0.455 |
| bta04964 Proximal tubule bicarbonate reclamation | 63.63636 | 0.455 |
| bta05204 Chemical carcinogenesis | 57.14286 | 0.455 |
| bta00250 Alanine. aspartate and glutamate metabolism | 59.45946 | 0.471 |
| bta00910 Nitrogen metabolism | 64.70588 | 0.471 |
| bta04145 Phagosome | 54.70588 | 0.471 |
| bta04975 Fat digestion and absorption | 58.33333 | 0.471 |
| bta04977 Vitamin digestion and absorption | 61.53846 | 0.471 |
| bta00030 Pentose phosphate pathway | 60.71429 | 0.4712 |
| bta00650 Butanoate metabolism | 60.71429 | 0.4712 |
| bta04122 Sulfur relay system | 70 | 0.4712 |
| bta04929 GnRH secretion | 56.92308 | 0.4712 |
| bta00630 Glyoxylate and dicarboxylate metabolism | 60 | 0.4775 |
| bta04723 Retrograde endocannabinoid signaling | 54.60526 | 0.48 |
| bta00051 Fructose and mannose metabolism | 58.82353 | 0.4897 |
| bta00061 Fatty acid biosynthesis | 61.11111 | 0.5172 |
| bta00730 Thiamine metabolism | 61.11111 | 0.5172 |
| bta01210 2-Oxocarboxylic acid metabolism | 61.11111 | 0.5172 |
| bta04650 Natural killer cell mediated cytotoxicity | 54.19847 | 0.5172 |
| bta00520 Amino sugar and nucleotide sugar metabolism | 56 | 0.5268 |
| bta00511 Other glycan degradation | 59.09091 | 0.532 |
| bta00534 Glycosaminoglycan biosynthesis | 58.33333 | 0.538 |
| bta00430 Taurine and hypotaurine metabolism | 61.53846 | 0.5385 |
| bta03018 RNA degradation | 54.43038 | 0.5385 |
| bta03320 PPAR signaling pathway | 54.32099 | 0.5385 |
| bta03450 Non-homologous end-joining | 61.53846 | 0.5385 |
| bta04213 Longevity regulating pathway | 54.83871 | 0.5385 |
| bta04614 Renin-angiotensin system | 57.69231 | 0.5385 |
| bta00983 Drug metabolism | 53.94737 | 0.5529 |
| bta05416 Viral myocarditis | 53.94737 | 0.5529 |
| bta05140 Leishmaniasis | 53.84615 | 0.5535 |
| bta00360 Phenylalanine metabolism | 56.52174 | 0.5602 |
| bta00980 Metabolism of xenobiotics by cytochrome P450 | 53.73134 | 0.5602 |
| bta03022 Basal transcription factors | 54.54545 | 0.5602 |
| bta03060 Protein export | 56.52174 | 0.5602 |
| bta03440 Homologous recombination | 54.7619 | 0.5602 |
| bta04672 Intestinal immune network for IgA production | 53.57143 | 0.5766 |
| bta00920 Sulfur metabolism | 60 | 0.578 |
| bta03013 RNA transport | 52.24719 | 0.578 |
| bta03410 Base excision repair | 54.54545 | 0.578 |
| bta00350 Tyrosine metabolism | 53.84615 | 0.5823 |
| bta01523 Antifolate resistance | 53.48837 | 0.5859 |
| bta00604 Glycosphingolipid biosynthesis | 56.25 | 0.5873 |
| bta00140 Steroid hormone biosynthesis | 52.23881 | 0.6033 |
| bta00982 Drug metabolism | 52.38095 | 0.6033 |
| bta04623 Cytosolic DNA-sensing pathway | 52.23881 | 0.6033 |
| bta05033 Nicotine addiction | 52.5 | 0.6126 |
| bta04973 Carbohydrate digestion and absorption | 52.27273 | 0.6141 |
| bta04620 Toll-like receptor signaling pathway | 50.90909 | 0.6514 |
| bta04940 Type I diabetes mellitus | 50.84746 | 0.6555 |
| bta03008 Ribosome biogenesis in eukaryotes | 50.60241 | 0.6622 |
| bta04060 Cytokine-cytokine receptor interaction | 50.77399 | 0.6641 |
| bta03040 Spliceosome | 50.34014 | 0.6748 |
| bta04979 Cholesterol metabolism | 50 | 0.6748 |
| bta05134 Legionellosis | 50 | 0.6748 |
| bta05332 Graft-versus-host disease | 50 | 0.6748 |
| bta04950 Maturity onset diabetes of the young | 50 | 0.6758 |
| bta04612 Antigen processing and presentation | 49.41176 | 0.7012 |
| bta00040 Pentose and glucuronate interconversions | 48.3871 | 0.7142 |
| bta05203 Viral carcinogenesis | 49.79253 | 0.715 |
| bta00053 Ascorbate and aldarate metabolism | 48 | 0.7171 |
| bta00830 Retinol metabolism | 48.4375 | 0.723 |
| bta00603 Glycosphingolipid biosynthesis | 47.05882 | 0.7305 |
| bta00860 Porphyrin and chlorophyll metabolism | 47.5 | 0.7327 |
| bta04714 Thermogenesis | 49.37238 | 0.7327 |
| bta00790 Folate biosynthesis | 47.22222 | 0.7329 |
| bta00072 Synthesis and degradation of ketone bodies | 45.45455 | 0.7484 |
| bta03050 Proteasome | 45.65217 | 0.7857 |
| bta05143 African trypanosomiasis | 45.45455 | 0.7861 |
| bta05144 Malaria | 45.76271 | 0.7938 |
| bta04080 Neuroactive ligand-receptor interaction | 48.76033 | 0.7952 |
| bta04630 JAK-STAT signaling pathway | 47.52475 | 0.8171 |
| bta05330 Allograft rejection | 44.64286 | 0.8171 |
| bta05150 Staphylococcus aureus infection | 45.71429 | 0.8334 |
| bta00450 Selenocompound metabolism | 38.88889 | 0.8505 |
| bta02010 ABC transporters | 43.33333 | 0.8505 |
| bta04932 Non-alcoholic fatty liver disease (NAFLD) | 46.20253 | 0.8505 |
| bta04610 Complement and coagulation cascades | 44.56522 | 0.8511 |
| bta05010 Alzheimer disease | 45.55556 | 0.8818 |
| bta04742 Taste transduction | 41.77215 | 0.9116 |
| bta04974 Protein digestion and absorption | 42.97521 | 0.9216 |
| bta04260 Cardiac muscle contraction | 41.57303 | 0.923 |
| bta04622 RIG-I-like receptor signaling pathway | 41.17647 | 0.94 |
| bta05016 Huntington disease | 44.89051 | 0.94 |
| bta00120 Primary bile acid biosynthesis | 29.41176 | 0.9464 |
| bta05310 Asthma | 34.21053 | 0.9558 |
| bta05168 Herpes simplex virus 1 infection | 45.16129 | 0.9583 |
| bta05320 Autoimmune thyroid disease | 36.61972 | 0.9712 |
| bta05012 Parkinson disease | 40 | 0.9799 |
| bta00970 Aminoacyl-tRNA biosynthesis | 33.33333 | 0.9883 |
| bta05206 MicroRNAs in cancer | 42.12329 | 0.9883 |
| bta00190 Oxidative phosphorylation | 37.14286 | 0.9951 |
| bta05034 Alcoholism | 39.30131 | 0.9967 |
| bta03010 Ribosome | 25 | 1.0 |
| bta04740 Olfactory transduction | 6.550218 | 1.0 |
| bta05322 Systemic lupus erythematosus | 24.72527 | 1.0 |
| \| ^1^%: Percent of genes predicted to be modulated. ^2^BH: Benjamini – Hochberg \| \| --- \| | | |
