# Supplementary material for: High body energy reserve influences extracellular vesicles miRNA contents within the ovarian follicle
Source: PLoS One. 2023 Jan 10;18(1):e0280195. doi: 10.1371/journal.pone.0280195 (PMC9831338; doi:10.1371/journal.pone.0280195)
Supplement: S12 Table — (DOCX) [file pone.0280195.s015.docx]

| **Supplementary table 12**. Biological patwhays predicted as modulated by miRNAs up regulated in follicular fluid extracellular vesicles (EV FF) compared to cumulus cells (CC) from ipsi and contralateral ovarian follicles (3-6 mm in diameter) from cows with high body energy reserve (HBER). | | |
| --- | --- | --- |
| **Pathway** | **%^1^** | **BH^2^** |
| bta04010 MAPK signaling pathway | 57.87671 | 0.0 |
| bta04144 Endocytosis | 57.95918 | 0.0 |
| bta04360 Axon guidance | 62.92135 | 0.0 |
| bta05200 Pathways in cancer | 51.10701 | 0.0 |
| bta01100 Metabolic pathways | 44.53176 | 0.0053 |
| bta04014 Ras signaling pathway | 56.19835 | 0.0053 |
| bta04072 Phospholipase D signaling pathway | 60.52632 | 0.0071 |
| bta04910 Insulin signaling pathway | 62.14286 | 0.0071 |
| bta05205 Proteoglycans in cancer | 57.07317 | 0.0071 |
| bta04015 Rap1 signaling pathway | 56.01852 | 0.0087 |
| bta04310 Wnt signaling pathway | 59.25926 | 0.0087 |
| bta04390 Hippo signaling pathway | 58.97436 | 0.0098 |
| bta04810 Regulation of actin cytoskeleton | 55.45024 | 0.0098 |
| bta04012 ErbB signaling pathway | 66.66667 | 0.0137 |
| bta04660 T cell receptor signaling pathway | 62.61682 | 0.0149 |
| bta04068 FoxO signaling pathway | 59.54198 | 0.016 |
| bta04062 Chemokine signaling pathway | 54.78723 | 0.0192 |
| bta05211 Renal cell carcinoma | 67.60563 | 0.0192 |
| bta05220 Chronic myeloid leukemia | 66.23377 | 0.0192 |
| bta05225 Hepatocellular carcinoma | 55.74713 | 0.0192 |
| bta05212 Pancreatic cancer | 65.78947 | 0.0213 |
| bta01522 Endocrine resistance | 61.70213 | 0.0248 |
| bta04071 Sphingolipid signaling pathway | 58.33333 | 0.0248 |
| bta04120 Ubiquitin mediated proteolysis | 56.42857 | 0.0248 |
| bta04150 mTOR signaling pathway | 55.41401 | 0.0248 |
| bta04510 Focal adhesion | 53.0303 | 0.0248 |
| bta04550 Signaling pathways regulating pluripotency of stem cells | 56.33803 | 0.0248 |
| bta04611 Platelet activation | 57.85124 | 0.0248 |
| bta04659 Th17 cell differentiation | 59.29204 | 0.0248 |
| bta04722 Neurotrophin signaling pathway | 58.19672 | 0.0248 |
| bta05210 Colorectal cancer | 61.79775 | 0.0248 |
| bta00562 Inositol phosphate metabolism | 64.38356 | 0.0252 |
| bta05214 Glioma | 63.63636 | 0.0252 |
| bta04530 Tight junction | 53.67232 | 0.0254 |
| bta01212 Fatty acid metabolism | 67.24138 | 0.0272 |
| bta01521 EGFR tyrosine kinase inhibitor resistance | 62.5 | 0.0272 |
| bta04070 Phosphatidylinositol signaling system | 59.59596 | 0.0272 |
| bta04921 Oxytocin signaling pathway | 54.60526 | 0.0272 |
| bta04931 Insulin resistance | 58.18182 | 0.0272 |
| bta05132 Salmonella infection | 51.33929 | 0.0272 |
| bta05224 Breast cancer | 54.66667 | 0.0273 |
| bta04514 Cell adhesion molecules (CAMs) | 53.79747 | 0.0312 |
| bta04934 Cushing syndrome | 53.84615 | 0.0313 |
| bta05231 Choline metabolism in cancer | 58.58586 | 0.0313 |
| bta04666 Fc gamma R-mediated phagocytosis | 59.13978 | 0.032 |
| bta05235 PD-L1 expression and PD-1 checkpoint pathway in cancer | 59.13978 | 0.032 |
| bta04916 Melanogenesis | 57.84314 | 0.0334 |
| bta04020 Calcium signaling pathway | 50.9901 | 0.0378 |
| bta04933 AGE-RAGE signaling pathway in diabetic complications | 57.28155 | 0.0378 |
| bta05223 Non-small cell lung cancer | 62.68657 | 0.0378 |
| bta05135 Yersinia infection | 54.61538 | 0.0389 |
| bta04625 C-type lectin receptor signaling pathway | 56.60377 | 0.0394 |
| bta05163 Human cytomegalovirus infection | 49.38776 | 0.0397 |
| bta05226 Gastric cancer | 52.94118 | 0.0397 |
| bta04520 Adherens junction | 61.42857 | 0.0407 |
| bta05230 Central carbon metabolism in cancer | 62.12121 | 0.0411 |
| bta05165 Human papillomavirus infection | 46.95652 | 0.0488 |
| bta04928 Parathyroid hormone synthesis. secretion and action | 55.76923 | 0.0502 |
| bta04658 Th1 and Th2 cell differentiation | 56.12245 | 0.0519 |
| bta04919 Thyroid hormone signaling pathway | 54.23729 | 0.0519 |
| bta05215 Prostate cancer | 56.12245 | 0.0519 |
| bta04261 Adrenergic signaling in cardiomyocytes | 52 | 0.052 |
| bta04664 Fc epsilon RI signaling pathway | 60 | 0.052 |
| bta04728 Dopaminergic synapse | 52.98507 | 0.052 |
| bta04024 cAMP signaling pathway | 48.9083 | 0.0527 |
| bta05166 Human T-cell leukemia virus 1 infection | 48.71795 | 0.0533 |
| bta04370 VEGF signaling pathway | 62.06897 | 0.0544 |
| bta04140 Autophagy | 52.11268 | 0.056 |
| bta04371 Apelin signaling pathway | 52.14286 | 0.057 |
| bta05219 Bladder cancer | 66.66667 | 0.0576 |
| bta04022 cGMP-PKG signaling pathway | 50.29586 | 0.0603 |
| bta04151 PI3K-Akt signaling pathway | 45.8445 | 0.0603 |
| bta04152 AMPK signaling pathway | 52.84553 | 0.0603 |
| bta04750 Inflammatory mediator regulation of TRP channels | 54.36893 | 0.0603 |
| bta04920 Adipocytokine signaling pathway | 58.33333 | 0.0603 |
| bta04922 Glucagon signaling pathway | 54.36893 | 0.0603 |
| bta05160 Hepatitis C | 50.60976 | 0.0603 |
| bta04540 Gap junction | 55.55556 | 0.0619 |
| bta00533 Glycosaminoglycan biosynthesis | 92.85714 | 0.062 |
| bta04670 Leukocyte transendothelial migration | 53.09735 | 0.064 |
| bta04730 Long-term depression | 60 | 0.064 |
| bta05100 Bacterial invasion of epithelial cells | 57.53425 | 0.064 |
| bta05418 Fluid shear stress and atherosclerosis | 51.03448 | 0.064 |
| bta04912 GnRH signaling pathway | 54.83871 | 0.0644 |
| bta04935 Growth hormone synthesis. secretion and action | 52.54237 | 0.0651 |
| bta05161 Hepatitis B | 49.7076 | 0.0651 |
| bta04662 B cell receptor signaling pathway | 55.17241 | 0.068 |
| bta04926 Relaxin signaling pathway | 51.53846 | 0.068 |
| bta00410 beta-Alanine metabolism | 67.64706 | 0.0703 |
| bta04066 HIF-1 signaling pathway | 52.72727 | 0.0703 |
| bta05213 Endometrial cancer | 59.32203 | 0.0703 |
| bta05218 Melanoma | 56.16438 | 0.0797 |
| bta04721 Synaptic vesicle cycle | 55.12821 | 0.0853 |
| bta04392 Hippo signaling pathway | 68.96552 | 0.0861 |
| bta05167 Kaposi sarcoma-associated herpesvirus infection | 47.57282 | 0.0879 |
| bta04218 Cellular senescence | 48.79518 | 0.089 |
| bta00071 Fatty acid degradation | 61.90476 | 0.0934 |
| bta00230 Purine metabolism | 50 | 0.0934 |
| bta04380 Osteoclast differentiation | 50 | 0.0934 |
| bta04668 TNF signaling pathway | 50.84746 | 0.0944 |
| bta05170 Human immunodeficiency virus 1 infection | 46.5812 | 0.0944 |
| bta05020 Prion diseases | 65.625 | 0.0982 |
| bta04720 Long-term potentiation | 55.07246 | 0.1041 |
| bta04911 Insulin secretion | 52.94118 | 0.1061 |
| bta05412 Arrhythmogenic right ventricular cardiomyopathy (ARVC) | 53.94737 | 0.1061 |
| bta04917 Prolactin signaling pathway | 53.01205 | 0.1077 |
| bta05414 Dilated cardiomyopathy (DCM) | 51.51515 | 0.1077 |
| bta01200 Carbon metabolism | 50.44248 | 0.1084 |
| bta04130 SNARE interactions in vesicular transport | 63.63636 | 0.113 |
| bta04710 Circadian rhythm | 64.51613 | 0.1137 |
| bta05221 Acute myeloid leukemia | 54.41176 | 0.1147 |
| bta05216 Thyroid cancer | 60 | 0.1243 |
| bta05410 Hypertrophic cardiomyopathy (HCM) | 51.08696 | 0.1317 |
| bta04142 Lysosome | 48.48485 | 0.1336 |
| bta00514 Other types of O-glycan biosynthesis | 57.77778 | 0.1346 |
| bta01040 Biosynthesis of unsaturated fatty acids | 63.33333 | 0.1346 |
| bta00650 Butanoate metabolism | 64.28571 | 0.1359 |
| bta04061 Viral protein interaction with cytokine and cytokine receptor | 50.52632 | 0.1361 |
| bta05217 Basal cell carcinoma | 53.96825 | 0.1361 |
| bta04146 Peroxisome | 51.19048 | 0.1429 |
| bta00310 Lysine degradation | 53.0303 | 0.1471 |
| bta05017 Spinocerebellar ataxia | 50 | 0.1471 |
| bta05142 Chagas disease (American trypanosomiasis) | 48.69565 | 0.1491 |
| bta05145 Toxoplasmosis | 48.67257 | 0.1533 |
| bta00062 Fatty acid elongation | 62.06897 | 0.1556 |
| bta04141 Protein processing in endoplasmic reticulum | 46.38554 | 0.1557 |
| bta04211 Longevity regulating pathway | 50 | 0.1562 |
| bta04971 Gastric acid secretion | 51.31579 | 0.1562 |
| bta00515 Mannose type O-glycan biosynthesis | 65.21739 | 0.1571 |
| bta00600 Sphingolipid metabolism | 55.10204 | 0.1571 |
| bta04064 NF-kappa B signaling pathway | 48.62385 | 0.1571 |
| bta05202 Transcriptional misregulation in cancer | 45.54974 | 0.1571 |
| bta04929 GnRH secretion | 52.30769 | 0.1586 |
| bta04215 Apoptosis | 58.82353 | 0.1655 |
| bta04724 Glutamatergic synapse | 47.78761 | 0.1766 |
| bta00564 Glycerophospholipid metabolism | 48.07692 | 0.1818 |
| bta01524 Platinum drug resistance | 50 | 0.1818 |
| bta04657 IL-17 signaling pathway | 48.91304 | 0.1818 |
| bta04930 Type II diabetes mellitus | 54.34783 | 0.1846 |
| bta04725 Cholinergic synapse | 47.36842 | 0.1854 |
| bta05031 Amphetamine addiction | 50.72464 | 0.1854 |
| bta01210 2-Oxocarboxylic acid metabolism | 66.66667 | 0.1909 |
| bta00280 Valine. leucine and isoleucine degradation | 52.94118 | 0.1916 |
| bta04923 Regulation of lipolysis in adipocytes | 51.72414 | 0.1931 |
| bta04927 Cortisol synthesis and secretion | 50.76923 | 0.1931 |
| bta00270 Cysteine and methionine metabolism | 53.06122 | 0.1948 |
| bta05164 Influenza A | 44.75138 | 0.1963 |
| bta05169 Epstein-Barr virus infection | 43.85965 | 0.1963 |
| bta04270 Vascular smooth muscle contraction | 45.86466 | 0.2089 |
| bta05032 Morphine addiction | 47.82609 | 0.2089 |
| bta04961 Endocrine and other factor-regulated calcium reabsorption | 52 | 0.2153 |
| bta00900 Terpenoid backbone biosynthesis | 61.90476 | 0.216 |
| bta00770 Pantothenate and CoA biosynthesis | 63.15789 | 0.218 |
| bta04713 Circadian entrainment | 47 | 0.218 |
| bta04914 Progesterone-mediated oocyte maturation | 47.72727 | 0.218 |
| bta03320 PPAR signaling pathway | 48.14815 | 0.2195 |
| bta04350 TGF-beta signaling pathway | 47.31183 | 0.2195 |
| bta04330 Notch signaling pathway | 50.9434 | 0.2244 |
| bta05162 Measles | 44.73684 | 0.226 |
| bta05321 Inflammatory bowel disease (IBD) | 48.57143 | 0.2354 |
| bta04915 Estrogen signaling pathway | 44.92754 | 0.2361 |
| bta01230 Biosynthesis of amino acids | 47.94521 | 0.2462 |
| bta04114 Oocyte meiosis | 45.37815 | 0.2462 |
| bta04137 Mitophagy | 48.48485 | 0.2488 |
| bta04210 Apoptosis | 44.3662 | 0.2576 |
| bta04727 GABAergic synapse | 46.15385 | 0.2681 |
| bta04925 Aldosterone synthesis and secretion | 45.83333 | 0.2694 |
| bta05014 Amyotrophic lateral sclerosis (ALS) | 48.33333 | 0.2745 |
| bta05222 Small cell lung cancer | 45.74468 | 0.2764 |
| bta00440 Phosphonate and phosphinate metabolism | 83.33333 | 0.281 |
| bta04340 Hedgehog signaling pathway | 49.01961 | 0.2856 |
| bta00250 Alanine. aspartate and glutamate metabolism | 51.35135 | 0.2882 |
| bta04672 Intestinal immune network for IgA production | 48.21429 | 0.2897 |
| bta00030 Pentose phosphate pathway | 53.57143 | 0.292 |
| bta00220 Arginine biosynthesis | 57.89474 | 0.292 |
| bta04640 Hematopoietic cell lineage | 44.54545 | 0.292 |
| bta04966 Collecting duct acid secretion | 53.57143 | 0.292 |
| bta00910 Nitrogen metabolism | 58.82353 | 0.3024 |
| bta00640 Propanoate metabolism | 51.51515 | 0.3027 |
| bta00052 Galactose metabolism | 51.6129 | 0.3133 |
| bta00512 Mucin type O-glycan biosynthesis | 51.6129 | 0.3133 |
| bta04924 Renin secretion | 45.83333 | 0.3181 |
| bta03450 Non-homologous end-joining | 61.53846 | 0.3186 |
| bta04964 Proximal tubule bicarbonate reclamation | 54.54545 | 0.3193 |
| bta04972 Pancreatic secretion | 44.11765 | 0.3221 |
| bta05152 Tuberculosis | 41.91919 | 0.3241 |
| bta04110 Cell cycle | 43.08943 | 0.3408 |
| bta04970 Salivary secretion | 44.08602 | 0.3408 |
| bta04726 Serotonergic synapse | 43.10345 | 0.3481 |
| bta05416 Viral myocarditis | 44.73684 | 0.3481 |
| bta04960 Aldosterone-regulated sodium reabsorption | 48.64865 | 0.3493 |
| bta00020 Citrate cycle (TCA cycle) | 50 | 0.3542 |
| bta00380 Tryptophan metabolism | 46.80851 | 0.3586 |
| bta04213 Longevity regulating pathway | 45.16129 | 0.3664 |
| bta05133 Pertussis | 44.15584 | 0.3676 |
| bta03420 Nucleotide excision repair | 46.66667 | 0.3678 |
| bta04216 Ferroptosis | 46.66667 | 0.3678 |
| bta04620 Toll-like receptor signaling pathway | 42.72727 | 0.3706 |
| bta00513 Various types of N-glycan biosynthesis | 46.51163 | 0.3811 |
| bta05030 Cocaine addiction | 45.83333 | 0.3827 |
| bta05134 Legionellosis | 44.82759 | 0.3835 |
| bta05140 Leishmaniasis | 43.58974 | 0.3835 |
| bta00534 Glycosaminoglycan biosynthesis | 50 | 0.3896 |
| bta05340 Primary immunodeficiency | 46.34146 | 0.3896 |
| bta00240 Pyrimidine metabolism | 44.64286 | 0.3938 |
| bta00592 alpha-Linolenic acid metabolism | 48.27586 | 0.3939 |
| bta00051 Fructose and mannose metabolism | 47.05882 | 0.3968 |
| bta00350 Tyrosine metabolism | 46.15385 | 0.3968 |
| bta00760 Nicotinate and nicotinamide metabolism | 46.15385 | 0.3968 |
| bta05146 Amoebiasis | 41.88034 | 0.3968 |
| bta00010 Glycolysis Gluconeogenesis | 43.75 | 0.3973 |
| bta04962 Vasopressin-regulated water reabsorption | 44.89796 | 0.3973 |
| bta00510 N-Glycan biosynthesis | 44.23077 | 0.406 |
| bta00565 Ether lipid metabolism | 44.23077 | 0.406 |
| bta03015 mRNA surveillance pathway | 42.10526 | 0.406 |
| bta03440 Homologous recombination | 45.2381 | 0.406 |
| bta04115 p53 signaling pathway | 42.85714 | 0.406 |
| bta04976 Bile secretion | 42.16867 | 0.4242 |
| bta00061 Fatty acid biosynthesis | 50 | 0.4267 |
| bta00072 Synthesis and degradation of ketone bodies | 54.54545 | 0.4348 |
| bta03013 RNA transport | 39.88764 | 0.4572 |
| bta03030 DNA replication | 44.44444 | 0.4572 |
| bta04145 Phagosome | 40 | 0.4572 |
| bta00561 Glycerolipid metabolism | 41.79104 | 0.4689 |
| bta04623 Cytosolic DNA-sensing pathway | 41.79104 | 0.4689 |
| bta03020 RNA polymerase | 44.82759 | 0.4739 |
| bta04913 Ovarian steroidogenesis | 42.10526 | 0.4739 |
| bta05323 Rheumatoid arthritis | 40.38462 | 0.4845 |
| bta00500 Starch and sucrose metabolism | 43.75 | 0.4896 |
| bta04978 Mineral absorption | 41.81818 | 0.4896 |
| bta04744 Phototransduction | 44.44444 | 0.4909 |
| bta00531 Glycosaminoglycan degradation | 45.45455 | 0.4912 |
| bta00330 Arginine and proline metabolism | 41.66667 | 0.5074 |
| bta00630 Glyoxylate and dicarboxylate metabolism | 43.33333 | 0.5074 |
| bta00532 Glycosaminoglycan biosynthesis | 45 | 0.5154 |
| bta04060 Cytokine-cytokine receptor interaction | 38.39009 | 0.5166 |
| bta03410 Base excision repair | 42.42424 | 0.5215 |
| bta00340 Histidine metabolism | 43.47826 | 0.5346 |
| bta00591 Linoleic acid metabolism | 41.66667 | 0.5358 |
| bta00670 One carbon pool by folate | 44.44444 | 0.5366 |
| bta04621 NOD-like receptor signaling pathway | 38.58696 | 0.5366 |
| bta04650 Natural killer cell mediated cytotoxicity | 38.9313 | 0.5366 |
| bta04512 ECM-receptor interaction | 39.32584 | 0.5433 |
| bta04950 Maturity onset diabetes of the young | 42.30769 | 0.5435 |
| bta03018 RNA degradation | 39.24051 | 0.5559 |
| bta00601 Glycosphingolipid biosynthesis | 41.37931 | 0.5587 |
| bta04975 Fat digestion and absorption | 39.58333 | 0.5787 |
| bta01523 Antifolate resistance | 39.53488 | 0.5846 |
| bta04932 Non-alcoholic fatty liver disease (NAFLD) | 37.97468 | 0.5846 |
| bta05144 Malaria | 38.98305 | 0.5846 |
| bta00480 Glutathione metabolism | 38.33333 | 0.6162 |
| bta03460 Fanconi anemia pathway | 38.46154 | 0.6163 |
| bta04973 Carbohydrate digestion and absorption | 38.63636 | 0.6165 |
| bta04723 Retrograde endocannabinoid signaling | 37.5 | 0.618 |
| bta00100 Steroid biosynthesis | 40 | 0.6209 |
| bta04918 Thyroid hormone synthesis | 37.83784 | 0.6209 |
| bta00520 Amino sugar and nucleotide sugar metabolism | 38 | 0.6293 |
| bta00360 Phenylalanine metabolism | 39.13043 | 0.6321 |
| bta00563 Glycosylphosphatidylinositol (GPI)-anchor biosynthesis | 38.46154 | 0.6412 |
| bta04614 Renin-angiotensin system | 38.46154 | 0.6412 |
| bta05033 Nicotine addiction | 37.5 | 0.6531 |
| bta00260 Glycine. serine and threonine metabolism | 37.2093 | 0.6606 |
| bta04630 JAK-STAT signaling pathway | 36.63366 | 0.6691 |
| bta00430 Taurine and hypotaurine metabolism | 38.46154 | 0.6778 |
| bta00604 Glycosphingolipid biosynthesis | 37.5 | 0.6851 |
| bta03022 Basal transcription factors | 36.36364 | 0.6851 |
| bta05143 African trypanosomiasis | 36.36364 | 0.6851 |
| bta00511 Other glycan degradation | 36.36364 | 0.6986 |
| bta00983 Drug metabolism | 35.52632 | 0.72 |
| bta04714 Thermogenesis | 35.98326 | 0.72 |
| bta00603 Glycosphingolipid biosynthesis | 35.29412 | 0.7289 |
| bta03430 Mismatch repair | 34.78261 | 0.7368 |
| bta04977 Vitamin digestion and absorption | 34.61538 | 0.7393 |
| bta00620 Pyruvate metabolism | 34.21053 | 0.7531 |
| bta05310 Asthma | 34.21053 | 0.7531 |
| bta00140 Steroid hormone biosynthesis | 34.32836 | 0.7641 |
| bta00730 Thiamine metabolism | 33.33333 | 0.7652 |
| bta04136 Autophagy | 33.33333 | 0.7709 |
| bta04940 Type I diabetes mellitus | 33.89831 | 0.7709 |
| bta00790 Folate biosynthesis | 33.33333 | 0.771 |
| bta05332 Graft-versus-host disease | 33.33333 | 0.7803 |
| bta05204 Chemical carcinogenesis | 33.76623 | 0.7833 |
| bta03008 Ribosome biogenesis in eukaryotes | 33.73494 | 0.7869 |
| bta00040 Pentose and glucuronate interconversions | 32.25806 | 0.7903 |
| bta04610 Complement and coagulation cascades | 33.69565 | 0.7903 |
| bta04612 Antigen processing and presentation | 32.94118 | 0.8209 |
| bta00120 Primary bile acid biosynthesis | 29.41176 | 0.8342 |
| bta00982 Drug metabolism | 31.74603 | 0.836 |
| bta03040 Spliceosome | 33.33333 | 0.836 |
| bta04260 Cardiac muscle contraction | 32.58427 | 0.836 |
| bta05206 MicroRNAs in cancer | 34.24658 | 0.836 |
| bta04217 Necroptosis | 33.33333 | 0.8465 |
| bta00450 Selenocompound metabolism | 27.77778 | 0.8481 |
| bta00590 Arachidonic acid metabolism | 31.70732 | 0.8481 |
| bta00860 Porphyrin and chlorophyll metabolism | 30 | 0.8481 |
| bta00980 Metabolism of xenobiotics by cytochrome P450 | 31.34328 | 0.8481 |
| bta00053 Ascorbate and aldarate metabolism | 28 | 0.8567 |
| bta04979 Cholesterol metabolism | 30 | 0.8567 |
| bta05330 Allograft rejection | 30.35714 | 0.8567 |
| bta05168 Herpes simplex virus 1 infection | 33.99504 | 0.8643 |
| bta04080 Neuroactive ligand-receptor interaction | 33.60882 | 0.8801 |
| bta05203 Viral carcinogenesis | 32.78008 | 0.8865 |
| bta04742 Taste transduction | 29.11392 | 0.9183 |
| bta05010 Alzheimer disease | 31.11111 | 0.9287 |
| bta05150 Staphylococcus aureus infection | 29.52381 | 0.9287 |
| bta05016 Huntington disease | 31.75182 | 0.9422 |
| bta00830 Retinol metabolism | 26.5625 | 0.9453 |
| bta03060 Protein export | 21.73913 | 0.9453 |
| bta04622 RIG-I-like receptor signaling pathway | 28.43137 | 0.9453 |
| bta05320 Autoimmune thyroid disease | 26.76056 | 0.9475 |
| bta03050 Proteasome | 21.73913 | 0.9871 |
| bta00190 Oxidative phosphorylation | 20.71429 | 1.0 |
| bta00970 Aminoacyl-tRNA biosynthesis | 18.18182 | 1.0 |
| bta02010 ABC transporters | 20 | 1.0 |
| bta03010 Ribosome | 13.125 | 1.0 |
| bta04740 Olfactory transduction | 4.89083 | 1.0 |
| bta04974 Protein digestion and absorption | 20.66116 | 1.0 |
| bta05012 Parkinson disease | 22.66667 | 1.0 |
| bta05034 Alcoholism | 24.89083 | 1.0 |
| bta05322 Systemic lupus erythematosus | 15.93407 | 1.0 |
| \| ^1^%: Percent of genes predicted to be modulated. ^2^BH: Benjamini – Hochberg \| \| --- \| | | |
