# Supplementary material for: High body energy reserve influences extracellular vesicles miRNA contents within the ovarian follicle
Source: PLoS One. 2023 Jan 10;18(1):e0280195. doi: 10.1371/journal.pone.0280195 (PMC9831338; doi:10.1371/journal.pone.0280195)
Supplement: S1 Raw image — (PDF) [file pone.0280195.s016.pdf]

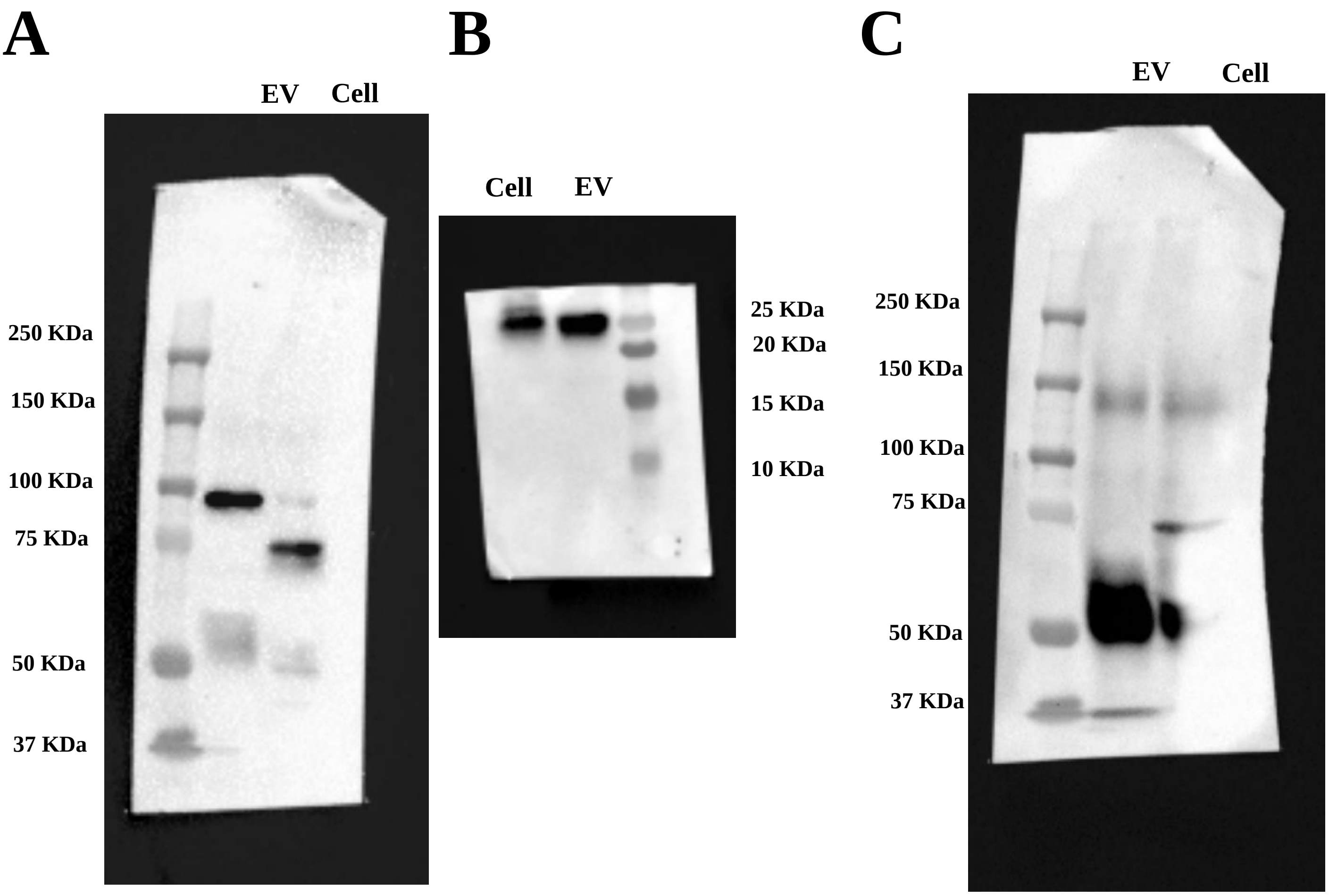

Raw image of Western Blot analysis. Original images of Western blot technique for follicular fluid extracellular vesicles characterization from cows with different body energy reserve. **A.** ALIX: ~95KDa. **B.** CD9: ~25KDa. **C.** GRP78: ~75KDa.

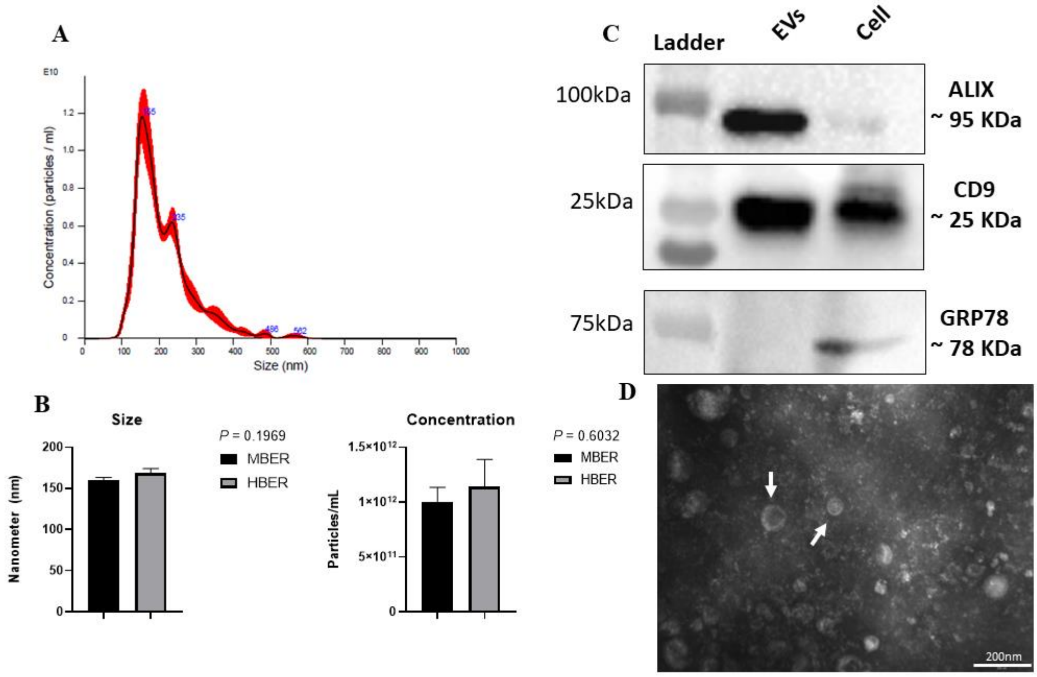

S3 Fig. Follicular fluid extracellular vesicles characterization. A. Follicular fluid extracellular vesicles from cows with different body energy reserve analyzed by nanoparticle tracking analysis (NTA). **B.** Extracellular vesicles size and concentration were analyzed by NTA**. C.** Western blotting analysis demonstrates the presence of characteristic vesicles proteins (ALIX and CD9) and absence of cell-specific proteins in follicular fluid vesicle samples (GRP78). The western blot images were cropped for the purpose of this Fig. **D.** Transmission electron microscopy images shows the presence of extracellular vesicles in follicular fluid.
